# Retrospective Observational Study on the Characteristics of Pain and Associated Factors of Breakthrough Pain in Advanced *Cancer* Patients

**DOI:** 10.1155/2022/8943292

**Published:** 2022-04-14

**Authors:** Rongrong Fan, Xuying Li, Siyu Yang, Xiaofan Bu, Yongyi Chen, Ying Wang, Cuiling Qiu

**Affiliations:** ^1^Department of Nursing Teaching and Research, Hunan Cancer Hospital, Affiliated Cancer Hospital of Xiangya Nursing School of Central South University, Changsha, China; ^2^Department of Nursing, Hunan Cancer Hospital, Affiliated Cancer Hospital of Xiangya Nursing School of Central South University, Changsha, China; ^3^Xiangya Nursing School of Central South University, Changsha, China; ^4^Department of Hospital Office, Hunan Cancer Hospital, Affiliated Cancer Hospital of Xiangya Nursing School of Central South University, Changsha, China; ^5^Department of Palliative Care Ward, Hunan Cancer Hospital, Affiliated Cancer Hospital of Xiangya Nursing School of Central South University, Changsha, China

## Abstract

**Objective:**

This study aimed to conduct a retrospective observational study to understand the status of characteristics of pain and identify potential variables influencing the clinical presentation of breakthrough cancer pain (BTP) in advanced cancer patients.

**Methods:**

Advanced cancer patients over 18 years of age; diagnosed with cancer of any type and stage III or IV in the palliative care ward with available data were enrolled between 2018 and 2020. Demographic data and pain-related information were collected by using structured electronic extraction form from Hospital Information System (HIS). Patients who had well-controlled background pain with an intensity ≤4 on a 0–10 numerical scale for >12 hours/day, the presence of transient exacerbations of pain with moderate-severe intensity (≧5), and clearly distinguish from background pain were regarded to have suffered BTP. Spearman correlation was conducted to explore the relationship between pain score and demographics characteristics. Factors significant in univariate analysis were included in the multiple regression model to explore independent predictive factors associated with the BTP.

**Results:**

Of 798 advanced cancer patients, the mean age was 56.7 (SD = 11.84) years. Lung cancer (29.95%) was the most common cancer, and pain (93%) was the most common symptom. More than half (*n* = 428, 53.6%) of the patients experienced BTP. The median number of BTP episodes was 4 (IQR = 2, 7, range: 1–42). The median intensity of BTP was 6 (IQR = 6, 7, range 5–10). Patients with severe background pain or BTP had longer hospital stay and more symptoms. Besides, more severe background pain was related to higher activity of daily living. Intramuscular injection of hydromorphone hydrochloride was the main medication for BTP onset. Younger age, background pain, anorexia, and constipation were independently associated with the presentation of BTP. BTP pain intensity was independently associated with bloating. Symptom numbers were an independent factor and positively associated with BTP episodes.

**Conclusions:**

BTP resulted in poor prognosis, which has a variable presentation depending on interdependent relationships among different characteristics. Good controlling of background pain and assessment of pain-related symptoms are essential for BTP management. BTP should be managed individually, especially the invisible pain among aged patients. Furthermore, BTP-related education and training were still needed.

## 1. Introduction

*Cancer* pain is one of the most frequent and disturbing of all cancer-related symptoms [1]. Unrelieved pain denies patients' comfort and greatly affects their activities, motivation, interactions with family and friends, and overall quality of life (QOL) [2]. Breakthrough pain (BTP) is a serious problem in cancer patients, with prolonged pain episodes and severe intensity despite analgesic [3], which was proved to be the independent risk factor of poor pain control [4]. BTP has not yet been clearly defined. Association for Palliative Medicine (APM) of Great Britain and Ireland defined BTP as a “transient exacerbation of pain that occurs either spontaneously, or in relation to a specific predictable or unpredictable trigger despite relatively stable and adequately controlled background pain” [5]. The prevalence of BTP is commonly high in cancer population. Meanwhile, the highest BTP prevalence was found in patients from palliative care or advanced cancer patients admitted to a hospice [6]. A prospective longitudinal study revealed that BTP episodes in terminally ill cancer patients reached an average of 7.2 episodes per patient over 7 days [7]. BTP is associated with a variety of physical, psychological, social complications, more disability, and decreased QOL [8–10]. However, managing BTP is challenging, for various factors like demographic data, diagnosis, psychological distress, sleep disturbances, cognitive function, addictive behavior, and even the performance status could lead to different BTP characteristics and response to treatment [6, 11]. Besides, BTP can have different causes, comorbidities, and pathophysiology, which make it complex to diagnose, assess, and manage [12]. For these heterogeneous natures, guidelines endorse the recommendation that management of BTP should be set on the individual patient's condition [8]. BTP can occur spontaneously or in relation to specific and predictable or unpredictable triggers [9]. About 30.5% of BTP were still predictable [13]. The predictability of BTP, no matter in occurrence or its characteristics, offers the chance of early identification and advanced intervention before an onset [14]. Meanwhile, it avoids undermanagement or overtreatment with opioids [15]. So far, some researches have been conducted to anticipate the BTP. Age, diagnosis, PS score, and background pain intensity were demonstrated to be associated with the BTP episodes and intensity in cancer patients [13]. The IQ-BTP is an 11-item questionnaire with satisfactory psychometric and validity properties, which enable potential BTP to be identified and differentiated into three likelihood classes (no BTP or high, intermediate, or low likelihood for BTP) [16]. Also, risk-prediction models for BTP were developed, and the accuracy between machine learning and regression techniques was compared, though in labour epidural analgesia [17]. Although BTP has been better characterized in recent years, failures to detect BTP remain common especially in advanced cancer patients. Effective management of BTP requires early prediction and reliable identification. Thus, this study aims to assess the status of BTP and identify potential variables influencing the presentation characteristics of BTP in advanced cancer patients, optimize the management of BTP, and provide enlightenment of BTP prediction model construction in further future.

## 2. Materials and Methods

### 2.1. Study Design and Participants

We performed a retrospective cross-sectional analysis of data obtained from the Hospital Information System (HIS) of a specialized tertiary cancer hospital located in Hunan, China. Convenience sampling was used, and patients who received palliative care from January 2018 to December 2020 were included.

Patients selected for this study met the following inclusion criteria: over 18 years of age and diagnosed with cancer of any type and stage III or IV according to the National *Cancer* Institute codes. Patients with ostensible cognitive deficits or serious psychiatric dysfunctions were excluded. Any case with missing data was excluded. Among those included populations, patients who had well-controlled and stable background pain with an intensity ≤4 on a 0–10 numerical scale for >12 hours/day, the presence of transient exacerbations of pain with moderate-severe intensity (≧5), and clearly distinguish from background pain were regarded to have suffered BTP.

The sample size was calculated based on the rate of BTP among cancer patients. The occurrence rate varies greatly in different studies, from 25.7% to 80% [18–21]. A multicenter, observational, cross-sectional study of 3,765 cancer patients showed that 48% suffered moderate BTP [6]. Considering the average value, the rate of 40% was used in the present study, and the sample size was calculated using the formula *N* = Z^2^PQ/(0.1P)^2^ where *Q* = 1 − P, *Z* = 1.96  (≈ 2.00) represents the chi-squared value [22]. The sample size calculated was *N* = 600. Given the possible 20% loss of data, the sample size of this study should be at least 750. This study was approved by the ethics committee of our hospital and conducted following the principles of the Helsinki Declaration.

### 2.2. Data Collection

Relevant data were obtained for analysis, which can be divided into two categories:

- General patient demographics, including age, gender, spouse (have or no), present residence (rural, city), education level (primary, middle, graduate, and above), body mass index [BMI, BMI < 18.4 (lower weight), 18.4 ≦ BMI ≦ 23.9 (normal weight), 24 ≦ BMI ≦ 27.9 (overweight), BMI ≧ 28 (obesity)], ECOG performance status score (PS), Barthel index, distress thermometer score (DT), the average length of stay, primary tumor, metastasis (site, number), and presence of symptoms (category, number). PS is a reliable indicator for a patient's general condition (0–5) [23], and we divided it into good PS group (0–2) and poor PS group (3–5). DT had been proved to be efficacious in screening for psychological distress in advanced cancer patients with pain (0–10) [24] with 4 as the cutoff value. Patients who scored greater than 4 were regarded to have psychological distress. Barthel index [25] is used to measure activities of daily living (ADL), ranging from 0–100, which can be divided into severely dependent (0–40), moderately dependent (41–60), slightly dependent (61–99), and independent (100). All the data were collected before the first BTP onset.
- For background pain, we collected mean pain score before the first BTP outset as basic background pain and pain score at discharge as an evaluation indicator of pain controlling during hospitalization. For BTP, median episodes, and intensity, major BTP interventions were recorded. The pain score was measured by the Numerical Rating Scale (NRS) ranging from 0–10, which has to be one of the most tools to measure pain intensity [26] and can be identified into three categories levels: mild (NRS 1–4), moderate (5–6), and severe (7–10) [27].

To improve the efficiency of data collection and avoid the bias, we designed a structured electronic extraction form, which was specifically designed for the study. All the information we need were listed, and collectors only need to import numbers we valued in advance, for example, binary variable “0” and “1” represented “no” and “yes”. All collectors had completed research courses (including medical statistics and nursing informatics) and received training on how to extract medical data. Details of extraction form and raw data of the participants were presented in Supplementary Material.

### 2.3. Statistical Analysis

Data were exported from Excel 2019 to SPSS Version 22.0 for analysis. Enumeration data were described as N (%) and median (interquartile range, IQR), while measurement data were described as mean ± standard deviation (SD). In order to find differences among demographic and clinical characteristics, chi-square was used for enumeration data, two-sample independent tests and variance analysis were used to evaluate measurement data of normal distribution, while Kruskal–Wallis Test and Mann–Whitney were used for measurement data of nonnormal distribution. Spearman correlation was conducted to explore the relationship between pain score and demographics characteristics. Factors significant in univariate analysis were included in multiple logistic regression model to explore independent predictive factors associated with the presence of BTP. Significant variables in univariate analysis then were selected in multiple linear regression for BTP intensity and episodes. The normal distribution of residuals was also tested to confirm the validation of the model. Variance inflation factor (VIF), tolerance, and factor analysis were used to judge multicollinearity. All statistical tests were two-sided, *P* values <0.05 were considered to be significant.

## 3. Results

### 3.1. Patient Characteristics

**Figure 1:**
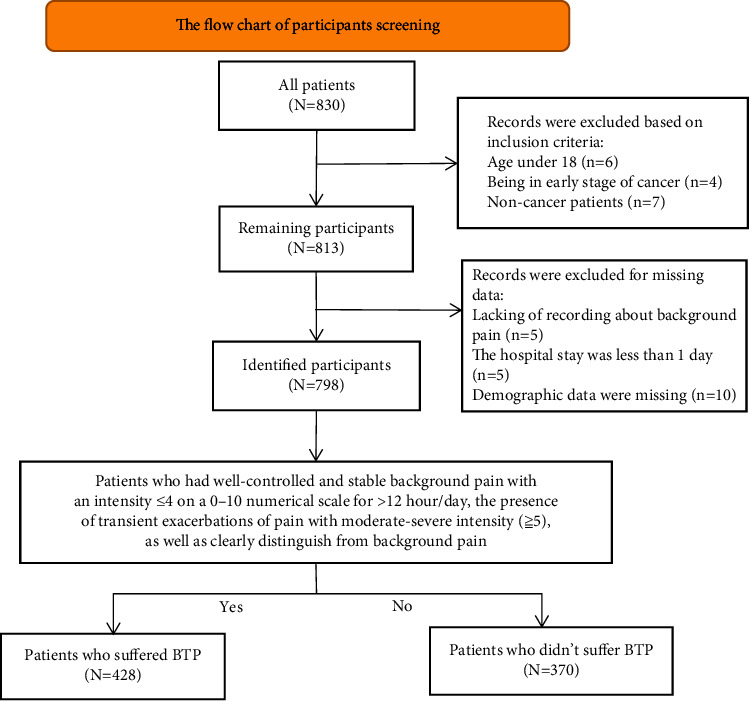
The flowchart of participants' screening.

The initial search retrieved 830 records. After meticulous inspection, 798 inpatients from 2018 to 2020 were enrolled in this study (Figure 1).  Table 1 summarizes the patients' characteristics. The age ranged from 16 to 85 years with the mean age being 56.7 (SD = 11.84) years. Most of them graduated from high school (53.3%). Majority of the samples (95.6%) have a spouse, and 55% lived in rural. Nearly half of the proportion (51%) had a normal BMI. Most samples had PS of 0–2 (69.8%), and 55.8% experienced distant metastasis with the most metastatic site in bone (31.1%). The most common cancer was lung cancer (29.95%), colorectal cancer (11.15%), and cervical cancer (7.14%). The most common symptom was pain (93%), followed by anorexia (36.5%) and sleep disorders (34.2%). More than half of the participants (60.7%) had symptoms no more than 4. The median distress score and Barthel index were 2 (2, 2) and 65 (50, 80), respectively.

### 3.2. Characteristics of Background Pain

The median background pain intensity on the day of assessment before the first BTP was 4 (3, 5). We divided patients into three levels based on background pain scores, and there were 69.3%, 26.2%, and 4.5% of patients in mild, moderate, and severe pain groups, respectively (Table 1). Sever background pain was related to longer hospital stay, higher Barthel index, and symptoms number (Table 2).

### 3.3. Characteristics of BTP

More than half (*n* = 428, 53.6%) of the patients experienced breakthrough. The median number of BTP episodes was 4 (IQR = 2, 7, range: 1–42). The median intensity of BTP was 6 (IQR = 6, 7, range 5–10). The majority of patients (*n* = 360, 84.1%) had an intensity of ≥7. Spearman correlation analysis showed BTP episodes and intensity were both negatively related to age (*r* = −0.081, −0.124, *P* < 0.05). Patients with BTP had longer hospital stay (*Z* = −7.134, *P* < 0.001), pain score at discharge (*Z* = −2.986, *P*=0.003), and more symptoms number (*Z* = −6.852, *P* < 0.001). Medications for BTP onset were intramuscular injection of hydromorphone hydrochloride (*n* = 116, 27.1%), subcutaneous morphine (*n* = 112, 26.16%), intramuscular injection of ketorolac tromethamine (*n* = 96, 22.43%), oral morphine (*n* = 36, 8.4%), intravenous analgesia (*n* = 24, 5.6%), PCIA (*n* = 20, 4.7%), and other analgesic ways (*n* = 24, 5.6%).

## 4. Factors Influencing BTP Clinical Presentation

### 4.1. Factors Influencing the Occurrence of Breakthrough Pain

Univariate analysis revealed age-group, education level, background pain level, symptoms number, metastasis number, bone metastasis, liver metastasis, the occurrence of anorexia, sleep disorder, constipation, and fatigue (Table 3) were significantly associated with higher BTP prevalence. There was no significant difference in BTP incidence among various cancer types (*χ*^2^ = 0.004–2.443, *P*=0.085–0.666).  Table 4 showed the results of multivariate analysis for BTP occurrence. Finally, older age, background pain level, the occurrence of anorexia, and constipation were confirmed. Factor analysis showed there existed no multicollinearity among independent variables.

### 4.2. Intensity of BTP

Mann–Whitney test and Kruskal–Wallis test showed that variables associated with a higher BTP intensity were BMI (*H* = 8.874, *P*=0.031) and bloating (*Z* = −1.973, *P*=0.049). There was no linear relation between BTP intensity and BMI, so dummy variables were set.  Table 5 shows the results of multivariate analysis and bloating was an independent influencing factor of BTP intensity. The residual was in normal distribution, and there existed no multiple collinearity.

### 4.3. Number of BTP Episodes during Hospitalization

Mann–Whitney test and Kruskal–Wallis test showed that variables associated with higher BTP episodes were colorectal cancer (*Z* = −2.058, *P*=0.04), distress score (*Z* = −2.222, *P*=0.026), bloating (*Z* = −3.01, *P*=0.03), anorexia (*Z* = −2.714, *P*=0.007), sleep disorder (*Z* = −2.599, *P*=0.009), fatigue (*Z* = −3.165, *P*=0.002), and symptoms number (*H* = 18.011, *P* < 0.001). Symptoms number was positively related with BTP episodes (*r* = 0.279, *P* < 0.001). Considered the multicollinearity among symptoms, we choose stepwise selection in multiple linear regression. More symptom numbers were independently associated with a higher number of BTP episodes (Table 6). The residual was in normal distribution.

## 5. Discussion

### 5.1. Characteristics of Background Pain and BTP

Cancer-related pain was the most popular symptom [28, 29]. A systematic review concluded in advanced cancer that 35% to 96% of cancer patients experienced pain [30], while more than 50% of cancer patients experienced moderate to severe pain [31]. Though, totally, patients had mild background pain score in our study, more than 50% then experienced BTP with high episodes and intensity, which was consistent with current studies that the BTP occurrence rate varies from 25.7% to 80% [18–21]. It seemed that there was an interaction between background pain and BTP. On the one hand, high background pain intensity was proved to be associated with frequent BTP [21]. We also revealed the slight positive correlation between BTP episodes and background level. Unfortunately, though patients with severe background pain score had stronger BTP intensity, significant statistical difference was not found. However, the importance of optimizing background pain management still needs to be emphasized, for optimization of background analgesia is vitally important to decrease BTP episodes, peak intensity, and duration [32, 33]. On the other hand, patients with BTP had higher pain score at discharge, indicating the controlling of BTP is helpful to improve treatment outcome.

Existing suffering of background pain or BTP aggravated the physical and symptoms burden. We found that the patients with BTP and those with more severe background pain score had longer hospital stays and more symptoms numbers, as the previous study showed [34]. Our study showed the occurrence of pain or BTP was related to poor prognosis, which was consistent with the finding that patients with BTP had more pain-related interference in function, worse physical health and mental health, and more disability [18]. Pain was considered to have strong influence on ADL difficulties [35]. Interestingly, we found the severe background pain was associated with better activity of daily life, which was contradictory to some conclusions that pain intensity was significantly negatively correlated with ADL [36, 37]. The possible interpretation was patients with better ADL more easily triggered the pain due to more activity. For example, patients may experience incident pain each time they get up from a chair or perform another specific activity [14]. Thus, NCCN guideline is recommended to optimize activities of daily living [2]. Activity movement was also proved to be the principal triggers for predictable BTP [13, 38], though there was no relationship between ADL and BTP in our study. No statistical difference was found between pain, BTP, and cancer types. We hypothesized that the participants were all in advanced stage. Thus, the cancer type played a less important role compared with disease condition.

Increased odds of persistent pain seemed to be related to younger age. Our study showed the elder (age ≧ 65) had a lower prevalence of BTP compared to younger groups. Meanwhile, age was negatively related to BTP intensity and episodes, and age was one of the independent influencing factors of BTP presentation. Sebastiano et al. found younger patients had higher background pain, a fast onset, and predictable BTP [13]. Pain is inherently subjective, and patient self-report is the current standard for assessment. However, less pain manifestation of elderly does not mean the less pain-related suffering, but some complications made self-reporting of pain more difficult. One of the biggest challenges is that most older adults and health-care practitioners perceive that pain is a normal part of aging, which impede history taking and pain expression [14]. Communication may be another challenge for memory failure, impaired cognition, sensory impairment (visual, hearing, and circulation problems), and stoicism with aging that can impact both assessment and management of pain older adults [39]. For elderly, prioritizing pain assessment so as to decrease the chance of inadequate analgesia is important. In addition to self-assessment pain, observation of pain-related behaviors and discomfort (like facial expression, body movements, changes in interpersonal interactions, and routine activity) are alternative strategies for assessing the presence of pain [40].

As for the intervention of BTP, opioids are the drug rescue of choice for BTP and guideline recommends offering oral immediate-release morphine for the first-line rescue medication of BTP [41, 42]. Recent data indicate that there were large disparities in the use of opioid analgesics to control BTP worldwide [43], due to cultural differences and overall awareness. We found intramuscular injection of hydromorphone hydrochloride and subcutaneous morphine were the most common analgesic form for BTP onset, while oral morphine accounted for only 8.4%. The reason for not adopting oral morphine as first-line treatment for BTP was as follows. Firstly, patients usually are reluctant to choose oral analgesics for they harbor a variety of fears and misconceptions such as opioid addiction, tolerance, and series of side effects [44]. Second is the unconsciousness or low compliance of medical members to guideline. Recent study showed despite oncologist's clinical practice on BTP treatment was increasingly guided by clinical guideline, it suffers from limited compliance [45]. Our study indicated improved dissemination and education were needed to enhance the awareness and guideline implementation in Chinese context.

### 5.2. Factors Influencing BTP Clinical Presentation

BTP was proved to be related to the presence of more than one pain, a vertebral pain syndrome, pain due to plexopathy, and English-speaking country [46]. This study indicates that BTP may have different characteristics and influence by many factors especially various concomitant symptoms. Patients aged <65 accompanied by moderate and severe background pain and the occurrence of symptoms including anorexia and constipation easily suffer BTP. There were some studies that proved compared to adults, the old-age group experienced less BTP [47]. Older patients might have multiple complex and serious complications which obstructed the presentation of BTP. A cross-sectional study reported subjects with older age had lower odds of reporting cancer alarm symptoms [48]. Our study showed patients with anorexia, fatigue, and constipation more easily suffer BTP. Symptoms like fatigue, sleep disorders, and anorexia were the common symptoms for cancer patients, which easily promoted the occurrence of the pain [42]. Anorexia and fatigue were often related to reduced energy intake, which lead to malnutrition and muscle strength. There were some researches that have proved the greater muscle strength was associated with less pain [37, 49, 50]. A randomized controlled trial revealed muscle strengthening and balancing exercises were effective in reducing chemotherapy-induced peripheral neuropathic pain and improving QOL among cancer patients [51]. Also, nutrition interventions had a significant effect on pain reduction [52]. The finding emphasized the importance to strengthen nutrition and recovery of myodynamia to reduce the possibility of BTP for patients with potential nutrition-related risk factors. Constipation is one of the most common possible side effects of analgesia (such as opioids) for moderate-to-severe pain (BTP) [53], which is not only a common factor contributing to pain but also a reversible cause of BTP. Thus, using laxatives is recommended during constipation to avoid BTP. Moderate and severe background pain was proved to be an influencing factor of BTP presentation. That's because higher background pain intensity may favor the development of BTP episodes, and severe background pain intensity was a powerful predictor of BTP scores [13]. Bloating was the only independent influencing factor of BTP intensity, which can be caused by constipation.

Symptoms number was proved to be the independent influencing factor of BTP episodes. The more the symptoms, the more the episodes. We hypothesize that less symptoms present a related good situation and thus less likely to suffer BTP onset. Symptoms and BTP onset interacted with each other. On one hand, symptoms promote the occurrence of BTP, and on the other hand, BTP results in significantly worse outcomes on functional and symptom [54].

### 5.3. Strengths and Limitations of the Study

There was limited literature to explore the characteristics of BTP and its influencing factors among advanced cancer patients. Firstly, our study confirmed the high prevalence of BTP among advanced cancer patients in the palliative care ward and the status characteristics of BTP episodes and intensity. Again, we emphasized the importance of the background pain controlling for better BTP management as well as optimized BTP scheme for better treatment outcome. Severe background pain and BTP were both related to poor prognosis, like longer hospital stay and more symptoms. Besides, more severe background pain was related to higher activity of daily living. Further research about the reason why oral opioid is not used as first-line therapy for BTP and related solutions should be considered. Several influencing factors of BTP presentation, such as older age, background pain level, the occurrence of anorexia, and constipation, were identified. Meanwhile, the bloating and symptoms number were independent factors of BTP intensity and episodes, respectively. For these heterogeneous clinical presentations, our finding indicated the problem should be solved individually. We should pay attention to the potential masked pain among elderly. This study has certain limitations. One of them is a retrospective study in which data were collected from the medical record and the fact that the data collection was conducted in a single hospital. The findings may not apply to all patients with advanced cancer. Though some influencing factors of BTP were confirmed, our retrospective design does not allow to establish a causal relationship for this association. As for the possibility of time rhythm of BTP, we failed to collect its onset characteristics including onset time, time to maximum pain intensity, mean duration of untreated episodes, and time to meaningful pain relief after intervention. Further research is needed in this regard for better BTP management.

## Figures and Tables

**Table 1:** Demographic, clinical, and pain data characteristics of patients (*n* = 798).

Characteristics		Patients (*N*%)
Age (years)		56.7 ± 11.84
	18–64	573 (71.8)
	≧65	225 (28.2)

Gender	Male	452 (56.6)
	Female	346 (43.4)

Education level	Primary	268 (33.6)
	Middle school	425 (53.3)
	Graduate and above	105 (13.2)

Having spouse	Yes	763 (95.6)
	No	35 (4.4)

Present residence	City	359 (45)
	Rural	439 (55)

BMI		21.1 ± 3.86
	Low weight	211 (26.4)
	Normal	407 (51.0)
	Overweight	130 (16.3)
	Obesity	50 (6.3)

PS score		2 (1, 3)
	Good (0–2)	557 (69.8)
	Poor (3–5)	241 (30.2)

Metastasis	Yes	445 (55.8)
	No	353 (44.2)

Metastasis site	Bone	248 (31.1)
	Lymph	147 (18.42)
	Liver	122 (15.29)

Metastasis number	0	353 (44.20)
	1	201 (25.2)
	2	150 (18.80)
	3	94 (11.8)

Symptom numbers		4 (2, 6)
	≦4	484 (60.7)
	5–9	284 (35.6)
	≧10	30 (3.8)

Background pain score		4 (3, 5)
	Mild (1–4)	553 (69.3)
	Moderate (5–6)	209 (26.2)
	Severe (7–10)	36 (4.5)
Pain score at discharge		2 (2, 2)

Breakthrough pain	Yes	428 (53.6)
	No	370 (46.4)
BTP episodes		4 (2, 7)
BTP intensity		6 (6, 7)
Average length of stay		9 (6.75, 15)

Distress score		2 (2, 2)
	Severe distress (≦4)	783 (98.1)
	Mild stress (>4)	15 (1.9)

Barthel index		65 (50, 80)
	Severely dependent (0–40)	128 (16)
	Moderately dependent (41–60)	263 (33)
	Slightly dependent (61–99)	345 (43.2)
	Independent (100)	62 (7.8)

**Table 2:** Comparison and correlation analysis in patients with different background pain level.

	Background pain level	Median (IQR)	Coefficient	*P*
BTP intensity	Mild	6 (6, 7)	5.954^*∗*^	0.051
	Moderate	6 (6, 7)		
	Severe	7 (6, 7)		
			0.086^a^	0.074

BTP episodes	Mild	3 (2, 6)	3.633^*∗*^	0.163
	Moderate	4 (2, 7)		
	Severe	5 (2, 7)		
			0.107^a^	0.027

Length of stay	Mild	9 (6, 15)	3.287^*∗*^	0.038
	Moderate	10 (7, 15.5)		
	Severe	11 (8.25, 18.75)		
			0.05^a^	0.154

PS	Mild	2 (1, 3)	1.689^*∗*^	0.430
	Moderate	2 (1, 3)		
	Severe	2 (1, 2.75)		
			0.116^a^	0.001

Distress score	Mild	2 (2, 2)	2.631^*∗*^	0.268
	Moderate	2 (2, 2.5)		
	Severe	2 (1, 2)		
			0.039^a^	0.272

Barthel index	Mild	65 (50, 80)	6.061^*∗*^	0.048
	Moderate	60 (50, 75)		
	Severe	70 (55, 80)		
			−0.008^a^	0.822

Symptom numbers	Mild	4 (2, 6)	11.347^*∗*^	0.003
	Moderate	4 (2.5, 6)		
	Severe	4 (2, 6.75)		
			0.135^a^	<0.001

^*∗*^Kruskal–Wallis test; IQR, interquartile range; ^a^Spearman correlation.

**Table 3:** Univariate analysis of BTP incidence among demographic data.

		BTP (%)	*χ* ^2^	*P*			BTP (%)	*χ* ^2^	*P*
Age	18–64	57.2	10.641	<0.001	Liver and cholecyst cancer	Yes	62.7	1.810	0.177
	≧65	44.4				No	53		

Gender	Male	56.5	3.781	0.052	Lung cancer	Yes	50.8	1.064	0.302
	Female	49.7				No	54.8		

Education level	Primary	47.2	7.4	0.025	Breast cancer	Yes	47.7	0.653	0.419
	Middle	57.8							
	Advanced	53.3				No	54		

Spouse	Yes	54	0.923	0.337	Colorectal cancer	Yes	57.3	0.542	0.461
	No	45.7				No	53.2		

Living place	City	52.1	0.626	0.429	Cervical cancer	Yes	42.1	3.281	0.07
	Country	54.9				No	54.5		

BMI	1	55	0.686	0.877	Head and neck cancer	Yes	55.8	0.167	0.682
	2	52.8				No	53.4		
	3	55.4			Fatigue	Yes	60.9	7.428	0.006
	4	50				No	50.5		

Metastasis number	0	47.9	9.45	0.024	Pain	Yes	56.6	37.497	<0.001
	1	56.7				No	14.3		
	2	57.3			Constipation	Yes	65.2	15.432	<0.001
	≧3	62.8				No	49.5		

Bone metastasis	Yes	59.3	4.6	0.032	Anorexia	Yes	66.3	29.665	<0.001
	No	51.1				No	46.4		

Lymph metastasis	Yes	57.8	1.27	0.259	Sleep disorder	Yes	65.6	23.764	<0.001
	No	52.7				No	47.4		

Liver metastasis	Yes	62.3	4.344	0.037	Bloating	Yes	57.5	0.753	0.386
	No	52.1				No	53		

PS	Good	52.4	1.087	0.297	Cough	Yes	50.6	0.853	0.356
	Poor	56.4				No	54.5		

Distress score	Severe	60	0.249	0.618	Nausea and vomiting	Yes	58.3	2.505	0.114
	Mild	53.5				No	52		

Barthel index	Severe	51.6	1.963	0.58	Dyspnea	Yes	58.8	1.938	0.164
	Moderate	57							
	Slight	52.5							
	Independent	50				No	52.5		

Background pain level	1	44.7	52.282	<0.001	Symptom number	≦4	45.5	33.764	<0.001
	2	73.7				5–9	65.5		
	3	75				≧10	73.3		

**Table 4:** Multivariate analysis for presence of BTP.

	B	SE	Wald	*P*	AOR	95% CI	
Age (≧65)	−0.470	0.177	7.037	0.008	0.625	0.441	0.884
Primary school (ref)			4.410	0.110			
Middle school	0.329	0.176	3.505	0.061	1.390	0.985	1.962
Graduate and above	−0.007	0.257	0.001	0.979	0.993	0.601	1.643
Bone metastasis	0.090	0.225	0.160	0.689	1.094	0.705	1.699
Liver metastasis	0.280	0.265	1.121	0.290	1.324	0.788	2.225
No metastasis (ref)			0.896	0.826			
Metastasis number (1)	0.207	0.223	0.861	0.354	1.230	0.794	1.907
Metastasis number (2)	0.094	0.270	0.121	0.728	1.098	0.647	1.862
Metastasis number (≧3)	0.119	0.362	0.109	0.742	1.127	0.555	2.289
Symptom numbers (≦4) (Ref)			3.280	0.194			
Symptom numbers (5–9)	0.394	0.220	3.228	0.072	1.484	0.965	2.281
Symptom numbers (≧10)	0.426	0.491	0.752	0.386	1.531	0.585	4.006
Mild background pain (ref)			48.998	<0.001			
Moderate background pain	1.251	0.189	43.935	<0.001	3.495	2.414	5.061
Severe background pain	1.222	0.410	8.902	0.003	3.395	1.521	7.579
Anorexia	0.639	0.201	10.168	0.001	1.895	1.279	2.808
Sleep disorder	0.221	0.198	1.248	0.264	1.247	0.846	1.838
Constipation	0.391	0.187	4.372	0.037	1.479	1.025	2.133
Fatigue	−0.307	0.209	2.154	0.142	0.736	0.489	1.108

Ref, reference; B, regression coefficient; SE, standard deviation; Wald, Wald coefficient; AOR, adjusted odds ratio; CI, confidence interval; Hosmer–Lemeshow test, *P*=0.881; adjusted Nagelkerke, *R*^2^ = 19.5%; overall predictive ability, 66.4%; −2 log likelihood = 976.037.

**Table 5:** Multiple linear regression of BTP intensity.

	B	SE	Standard beta	*t*	*P*	95% CI	Tolerance	VIF
Normal (ref)								
Low weight	0.127	0.096	0.067	1.323	0.186	−0.062, 0.316	0.891	1.122
Overweight	−0.188	0.113	−0.083	−1.655	0.099	−0.411, 0.035	0.900	1.111
Obesity	0.323	0.176	0.090	1.835	0.067	−0.023, 0.670	0.950	1.053
Bloating	0.238	0.115	0.099	2.060	0.040	0.011, 0.464	0.998	1.002

*F* = 3.649, *P*=0.006; Ref, reference; B, standardized coefficients; SE, standard deviation; Durbin–Watson, 1.745.

**Table 6:** Multiple linear regression of BTP episodes during hospitalization.

	B	SE	Standard beta	*t*	*P*	95% CI	Tolerance	VIF
Symptoms number	0.302	0.093	0.156	3.255	0.001	0.120, 0.484	1.000	1.000

*F* = 10.593, *P*=0.001; B, standardized coefficients; SE, standard deviation; Durbin–Watson, 1.505.

## Data Availability

The data are provided in the Supplementary Information files.

## Supplementary Materials

Supplementary Materials Details of extraction form and raw data of the participants were presented in Supplementary Material.
