# Supplementary material for: Retrospective Observational Study on the Characteristics of Pain and Associated Factors of Breakthrough Pain in Advanced Cancer Patients
Source: Pain Res Manag. 2022 Apr 14;2022:8943292. doi: 10.1155/2022/8943292 (PMC9023204; doi:10.1155/2022/8943292)
Supplement: Supplementary Materials — Details of extraction form and raw data of the participants were presented in Supplementary Material. [file 8943292.f1.pdf]

Supplementary Material:extraction form and raw data of the participants

Number	Age	Gender（1male；2female）	Education level(1Primary；2Middle school；3Graduate and above)	Spouse(1yes；2no)	Present residence(1city；2rural)	BMI	Bone metastasis(1yes; 0no)	Lung metastasis(1yes; 0no)	Lymph metastasis(1yes; 0no)
1	41	1	2	1	2	1	0	0	0
2	72	1	2	1	1	2	0	0	0
3	57	2	2	1	2	1	1	0	0
4	44	1	2	1	2	1	1	1	1
5	55	2	2	1	1	2	0	0	0
6	62	1	2	1	1	4	0	0	0
7	68	1	3	1	1	2	1	0	0
8	69	1	2	1	2	1	0	0	0
9	67	1	3	1	1	2	1	0	0
10	57	1	3	1	1	2	0	0	1
11	55	1	3	1	1	3	1	1	0
12	49	2	2	1	1	1	1	0	0
13	47	2	2	1	1	2	1	0	1
14	61	1	2	1	2	2	1	0	0
15	57	1	3	1	2	1	0	0	0
16	61	1	2	1	1	1	0	0	0
17	85	1	2	1	1	2	0	0	0

18	77	2	1	1	2	1	0	0	0
19	52	2	2	1	2	2	0	0	1
20	44	2	1	1	2	2	0	0	1
21	58	2	1	1	1	4	0	1	0
22	36	2	3	1	1	1	0	0	1
23	48	1	2	1	2	2	1	0	0
24	62	1	1	1	2	2	0	0	0
25	69	1	3	1	2	2	1	0	1
26	65	1	2	1	1	2	1	1	0
27	63	1	1	1	2	2	1	0	0
28	52	2	2	1	2	2	0	0	0
29	50	1	2	1	1	1	1	1	0
30	55	2	3	1	1	3	1	0	1
31	47	2	2	1	1	1	0	0	0
32	32	2	3	1	1	4	0	0	1
33	57	2	2	1	2	3	1	0	1
34	69	2	1	1	1	2	1	0	0
35	61	2	2	1	1	2	1	0	1
36	53	2	2	1	1	2	1	0	0
37	40	2	3	2	1	2	0	1	0
38	61	2	2	1	1	2	0	0	1
39	78	2	1	1	1	1	0	0	1
40	70	2	2	1	1	3	1	1	1
41	68	2	2	1	2	2	0	0	0
42	51	2	1	1	2	2	1	0	0

43	45	2	2	1	1	2	0	0	1
44	57	1	1	1	1	1	0	0	0
45	58	1	2	1	2	2	1	0	1
46	69	2	2	1	1	2	0	0	0
47	68	2	2	1	1	1	0	0	0
48	66	1	2	1	1	1	0	1	0
49	60	1	2	1	2	2	0	0	0
50	48	2	2	1	2	4	1	1	0
51	61	1	3	1	2	1	0	0	0
52	34	1	2	1	2	2	1	0	1
53	69	1	2	1	1	2	1	1	0
54	61	1	2	1	1	3	1	0	0
55	66	1	2	1	2	2	0	0	0
56	68	1	2	1	1	2	0	1	1
57	50	2	2	1	2	3	0	1	1
58	60	2	2	1	1	1	1	0	1
59	69	1	1	1	1	1	0	0	0
60	56	2	2	1	1	2	1	1	1
61	54	2	3	1	1	2	0	0	0
62	70	2	3	1	1	2	1	1	0
63	57	1	2	1	2	1	1	0	0
64	67	2	2	1	2	1	0	0	0
65	52	1	2	1	1	1	1	0	1
66	81	2	1	1	2	3	0	0	0
67	57	2	2	1	2	2	0	0	0

68	63	2	2	1	2	3	1	1	1
69	54	1	1	1	2	2	0	0	0
70	79	1	2	1	1	4	1	0	0
71	57	1	2	1	2	3	1	0	0
72	50	2	2	1	1	2	1	0	1
73	54	1	1	1	2	1	1	1	0
74	51	2	1	1	2	3	0	0	0
75	52	2	2	1	2	2	0	0	1
76	53	2	2	1	2	2	0	0	1
77	69	1	2	1	1	2	1	0	0
78	51	2	2	1	1	1	0	0	0
79	74	1	2	1	1	2	0	0	0
80	63	1	2	1	2	2	0	0	1
81	52	1	2	1	1	3	1	0	0
82	53	1	2	2	1	2	0	0	1
83	64	2	1	1	1	2	0	0	1
84	64	1	1	1	2	2	0	0	0
85	36	2	2	1	2	1	0	0	1
86	43	1	3	1	1	3	0	1	0
87	47	1	1	1	2	2	1	0	0
88	62	1	2	1	1	1	1	0	0
89	60	1	3	1	1	3	0	0	1
90	49	2	3	1	1	1	1	0	1
91	56	1	2	1	2	2	0	1	1
92	43	1	1	1	2	1	0	0	1

93	72	2	2	1	1	3	0	1	0
94	71	1	2	1	1	2	1	0	0
95	57	1	2	1	1	2	1	0	0
96	67	1	1	1	2	1	1	0	0
97	62	1	2	1	1	1	0	0	1
98	66	1	3	1	1	1	1	0	0
99	56	1	2	1	1	1	1	0	1
100	54	2	2	2	2	2	1	0	0
101	62	2	2	2	1	4	1	0	0
102	56	1	3	2	1	3	0	1	0
103	56	2	2	2	2	1	1	0	0
104	55	2	2	1	1	2	1	1	0
105	48	1	2	1	2	4	1	0	0
106	40	1	1	1	2	2	0	0	0
107	65	1	2	1	1	2	1	0	0
108	40	2	3	1	1	2	1	0	0
109	66	2	1	1	1	1	1	0	0
110	53	1	2	1	1	2	1	0	0
111	55	1	2	1	2	3	1	1	0
112	28	2	3	2	1	2	0	1	0
113	53	1	2	1	2	1	0	0	1
114	52	1	2	1	2	1	1	0	0
115	38	1	2	1	2	2	1	1	0
116	36	2	2	1	2	3	1	0	0
117	50	2	2	1	2	1	1	1	1

118	53	2	2	1	2	2	1	0	0
119	57	2	1	1	2	4	1	0	1
120	80	2	1	1	1	2	0	0	1
121	53	2	3	1	1	2	1	1	0
122	65	2	2	1	2	2	1	1	1
123	56	2	1	1	1	1	1	1	0
124	48	2	2	1	1	4	0	0	0
125	59	2	1	2	1	3	0	1	0
126	61	1	2	1	2	3	1	0	0
127	65	1	3	1	1	2	1	0	0
128	61	2	2	1	1	1	0	0	1
129	41	2	2	1	2	2	0	0	0
130	48	2	1	1	2	3	0	0	0
131	79	1	3	1	1	4	0	0	0
132	71	1	2	1	1	1	0	0	0
133	56	1	1	1	2	2	0	0	1
134	45	2	1	1	2	3	1	0	0
135	68	2	2	1	1	4	0	0	0
136	45	1	1	1	1	1	0	0	1
137	68	2	1	1	2	2	0	0	0
138	72	1	2	1	1	3	0	0	0
139	51	1	2	1	1	1	1	0	0
140	75	1	2	1	2	3	1	0	1
141	47	2	2	1	2	1	0	0	0
142	60	2	1	1	1	2	0	0	0

143	45	1	3	1	1	1	0	0	1
144	31	1	2	1	2	1	0	0	0
145	26	2	3	2	2	1	1	0	0
146	66	2	1	1	1	1	0	0	1
147	52	1	1	1	2	1	0	1	0
148	54	1	2	1	1	2	0	1	0
149	59	1	3	1	1	2	0	0	0
150	37	2	2	1	2	1	0	0	0
151	61	1	2	1	2	3	0	0	0
152	77	1	2	1	2	2	0	0	0
153	42	1	3	1	1	2	1	0	0
154	69	1	1	1	1	2	0	0	0
155	54	1	3	2	1	3	1	0	0
156	48	2	2	1	1	2	0	0	1
157	63	1	2	1	2	2	0	0	0
158	46	2	2	1	1	1	0	0	0
159	69	1	1	1	2	2	1	1	0
160	42	1	3	1	1	1	0	0	1
161	63	1	1	1	1	2	0	0	0
162	49	1	2	1	2	1	1	0	1
163	49	2	2	1	1	2	0	0	0
164	44	1	2	1	1	1	0	0	1
165	50	1	2	1	1	3	1	0	0
166	32	1	2	1	1	2	0	0	0
167	33	1	3	1	1	2	0	0	0

168	73	1	2	1	1	3	1	0	0
169	78	1	1	1	1	2	1	0	0
170	65	1	1	1	1	2	0	0	0
171	33	1	3	1	2	1	0	1	1
172	34	1	1	1	1	1	0	0	0
173	69	1	2	1	2	3	1	0	0
174	29	2	3	1	2	1	0	0	0
175	51	2	2	1	1	2	1	1	1
176	62	1	2	1	2	2	0	0	0
177	47	1	2	1	1	3	0	0	0
178	54	2	2	1	1	2	1	1	0
179	56	2	1	1	2	2	1	0	0
180	47	1	1	1	1	2	0	0	0
181	51	1	2	1	2	3	1	0	1
182	42	2	2	1	2	1	0	0	0
183	72	2	1	1	1	2	1	0	0
184	55	2	2	1	2	2	0	0	0
185	65	2	2	1	1	2	0	0	0
186	26	2	3	1	1	1	0	0	0
187	80	2	1	1	2	1	0	0	0
188	60	2	1	1	1	3	0	0	1
189	72	2	2	1	1	2	0	0	0
190	51	1	2	1	2	4	1	0	0
191	59	1	3	1	2	3	1	0	0
192	49	1	1	1	1	2	1	0	0

193	49	2	1	1	2	3	0	0	1
194	60	2	2	1	1	2	1	0	1
195	45	2	2	1	1	3	1	0	0
196	62	1	2	1	1	2	1	0	0
197	66	1	1	1	2	2	1	0	0
198	57	1	2	1	1	2	0	0	0
199	67	2	2	1	1	2	0	0	0
200	75	1	2	1	1	2	1	0	0
201	65	2	1	1	1	1	0	0	0
202	48	1	2	1	2	3	1	0	1
203	60	1	2	1	2	2	0	0	0
204	53	1	2	1	2	2	1	0	1
205	69	2	1	1	2	2	0	0	0
206	84	2	1	1	2	2	0	0	0
207	53	2	3	1	1	2	0	0	0
208	52	1	1	1	2	2	0	0	0
209	70	2	2	1	1	3	0	0	0
210	72	1	1	1	2	1	0	0	0
211	54	2	2	1	1	1	1	1	1
212	50	1	2	1	1	2	1	0	0
213	45	1	3	1	1	2	0	0	0
214	69	2	2	1	1	2	0	0	0
215	65	2	1	1	1	3	0	0	0
216	72	1	3	1	1	3	1	0	0
217	55	1	2	1	1	3	0	0	0

218	69	1	1	1	2	4	1	0	0
219	65	2	1	1	1	2	1	0	0
220	51	1	1	1	1	1	0	0	0
221	54	1	1	1	1	1	1	0	0
222	73	1	2	1	1	2	1	0	1
223	61	1	2	1	1	2	0	0	0
224	43	1	3	1	2	2	1	1	0
225	67	2	1	1	2	4	0	0	1
226	64	1	2	1	1	1	0	0	0
227	54	2	2	1	2	2	0	0	0
228	69	1	1	1	2	1	1	0	0
229	66	1	2	1	2	1	0	0	0
230	62	1	1	1	2	1	0	0	1
231	49	2	1	1	2	3	1	0	0
232	66	1	1	1	2	2	1	0	0
233	52	1	2	1	2	2	0	0	0
234	50	2	1	1	2	2	0	1	1
235	66	2	1	1	2	4	1	0	0
236	62	1	3	1	1	2	1	0	0
237	66	1	2	1	1	2	0	1	0
238	58	2	1	1	2	2	1	0	0
239	48	1	2	1	1	1	1	0	0
240	70	2	3	1	1	3	0	0	0
241	55	2	1	1	2	2	1	0	0
242	50	2	2	1	1	2	0	0	1

243	39	1	2	1	2	2	0	0	0
244	49	1	1	1	1	1	0	0	0
245	51	1	2	1	2	2	0	0	0
246	64	1	3	1	1	2	0	0	0
247	35	1	3	1	2	2	0	0	0
248	72	2	1	2	1	3	0	0	0
249	47	1	1	1	2	1	0	0	0
250	43	2	1	1	2	2	0	1	0
251	50	1	1	1	2	2	1	0	0
252	39	1	2	1	2	2	1	0	0
253	69	2	1	1	2	2	0	1	1
254	70	2	1	1	2	3	1	0	1
255	55	1	2	1	1	2	0	0	0
256	56	2	2	1	1	3	1	0	1
257	60	2	1	1	2	2	0	0	0
258	47	2	1	1	2	1	0	0	0
259	69	1	1	1	2	2	0	0	1
260	32	1	3	1	1	1	0	1	0
261	71	1	2	1	1	1	0	0	0
262	57	2	3	2	1	2	0	0	0
263	70	2	1	1	1	1	0	0	0
264	51	2	2	1	2	1	1	0	1
265	72	1	2	1	1	4	0	0	0
266	54	1	2	1	2	2	0	1	1
267	62	1	1	1	2	2	0	0	0

268	51	1	2	1	2	2	0	0	0
269	53	1	2	1	1	1	0	0	0
270	64	1	2	1	1	2	0	0	0
271	52	1	2	1	1	3	1	0	0
272	66	2	1	1	2	4	1	0	1
273	46	1	1	1	2	4	0	0	0
274	77	2	2	1	1	3	1	0	0
275	50	1	1	1	2	4	0	0	0
276	45	1	1	1	2	3	0	1	1
277	56	2	1	1	2	2	0	0	0
278	66	1	2	1	1	2	1	0	0
279	51	1	3	2	1	4	0	0	0
280	62	1	2	1	1	2	0	0	0
281	60	1	2	1	1	2	0	0	0
282	61	1	1	1	1	1	0	0	0
283	83	2	1	2	1	3	0	0	0
284	65	1	1	1	1	2	0	0	0
285	72	1	1	1	2	2	1	0	0
286	16	1	2	1	2	4	0	0	0
287	66	1	2	1	1	1	1	0	0
288	66	1	2	1	1	1	1	0	0
289	67	2	1	1	2	2	1	0	1
290	61	1	2	1	2	2	1	0	0
291	42	1	3	1	1	2	1	0	0
292	39	1	3	1	1	2	1	1	0

293	59	2	1	1	2	3	1	0	0
294	44	2	2	1	1	2	1	0	0
295	81	2	2	1	1	1	0	0	0
296	63	1	1	1	1	1	1	0	1
297	55	1	2	1	1	3	1	1	0
298	46	1	2	1	2	2	1	0	1
299	31	1	3	1	2	3	1	1	0
300	42	2	1	1	2	3	0	0	0
301	67	2	1	1	1	1	0	0	0
302	56	1	2	1	1	1	1	1	0
303	68	1	2	1	2	1	0	0	0
304	41	1	2	1	2	4	0	1	0
305	82	1	2	1	1	3	0	0	0
306	54	1	2	1	1	1	0	0	0
307	65	2	2	1	1	1	0	0	0
308	68	1	2	1	2	2	0	0	0
309	47	2	3	1	1	2	0	0	0
310	55	2	2	1	2	2	0	0	0
311	55	1	1	1	2	2	0	0	0
312	63	2	1	1	2	3	1	1	1
313	72	2	3	1	1	4	1	1	0
314	26	2	2	1	1	1	0	0	0
315	52	1	2	1	2	2	1	0	1
316	42	2	2	1	2	2	0	1	1
317	70	1	2	1	2	3	1	0	0

318	50	2	2	1	2	2	1	1	1
319	57	1	2	1	2	2	0	0	0
320	41	1	3	1	1	2	0	0	0
321	62	2	2	1	1	2	0	0	0
322	60	2	2	1	1	1	0	0	0
323	56	2	1	1	2	4	0	0	0
324	53	1	2	1	2	2	1	0	1
325	66	1	1	1	2	2	0	0	0
326	73	1	2	1	1	1	1	0	0
327	53	1	2	1	1	2	0	0	0
328	33	1	2	1	1	2	1	0	0
329	55	1	2	1	1	1	0	0	0
330	73	1	1	1	2	1	0	0	0
331	61	1	2	1	2	1	1	1	0
332	58	1	2	1	2	2	0	0	0
333	55	1	2	1	2	2	0	0	0
334	74	1	1	1	2	3	0	0	0
335	70	1	2	1	2	4	0	0	0
336	50	1	2	1	2	3	0	0	0
337	47	1	2	1	1	2	0	0	0
338	68	1	1	1	1	3	0	0	0
339	62	1	2	1	2	3	0	0	0
340	26	2	2	1	2	1	0	1	0
341	33	2	2	1	2	2	0	0	0
342	60	1	2	1	2	3	0	0	0

343	66	1	1	1	2	2	0	0	0
344	40	1	2	1	1	2	1	0	0
345	44	2	3	1	1	1	0	0	0
346	59	1	2	1	1	3	0	0	0
347	62	1	2	1	1	1	0	0	0
348	55	1	2	1	2	2	0	0	0
349	31	1	2	2	1	2	0	0	0
350	33	2	2	1	2	2	0	0	0
351	68	1	3	1	1	1	1	1	0
352	50	2	2	1	1	2	0	0	0
353	37	2	2	1	2	2	0	0	0
354	68	2	1	2	2	1	0	1	0
355	72	2	1	1	2	2	0	0	0
356	45	1	2	1	1	2	0	0	0
357	50	2	1	1	2	3	0	0	0
358	82	2	1	2	1	1	0	0	0
359	63	2	1	1	2	2	0	0	0
360	42	2	2	1	2	3	0	0	0
361	62	2	1	1	2	1	0	0	0
362	58	2	2	1	1	2	0	0	0
363	49	1	2	1	2	2	0	0	0
364	70	1	2	1	2	2	0	0	0
365	71	2	1	1	2	2	0	0	0
366	67	1	1	1	2	2	0	0	0
367	51	2	2	1	2	3	0	0	0

368	68	2	1	1	2	3	0	0	0
369	50	2	2	1	2	2	1	0	0
370	55	1	2	1	1	1	1	0	0
371	45	1	1	2	2	3	0	0	0
372	55	2	1	1	1	1	0	0	0
373	38	1	2	1	2	2	0	0	0
374	64	2	1	1	1	4	0	0	0
375	54	1	2	1	2	1	1	0	0
376	49	1	2	1	2	4	0	0	0
377	65	2	2	1	1	1	1	1	0
378	61	1	1	1	2	2	0	0	0
379	37	1	2	1	2	2	0	0	0
380	45	1	3	1	2	3	0	0	0
381	45	2	1	1	1	3	0	0	1
382	57	1	2	1	1	2	1	0	0
383	53	1	1	1	2	3	1	0	1
384	66	1	3	1	1	2	1	0	0
385	68	2	1	1	2	2	1	0	0
386	50	1	2	1	2	2	0	0	0
387	54	2	2	1	2	2	0	0	0
388	62	1	2	1	2	2	0	0	0
389	62	1	1	1	2	1	0	0	0
390	61	2	2	1	1	2	1	0	0
391	71	1	1	1	2	1	0	0	0
392	71	1	1	1	2	4	0	0	0

393	53	1	2	1	1	1	0	0	0
394	48	1	1	1	2	1	0	0	0
395	72	1	2	1	1	2	1	0	0
396	48	1	2	1	2	3	0	1	0
397	55	1	3	1	1	2	0	0	0
398	55	2	1	1	1	1	1	0	0
399	65	1	1	1	2	2	0	0	0
400	71	1	3	1	2	2	1	0	0
401	45	1	2	1	2	2	0	0	0
402	54	2	2	1	2	3	1	0	0
403	45	1	2	1	2	2	0	0	0
404	54	1	2	1	1	2	0	0	0
405	76	1	1	1	1	1	0	0	0
406	71	1	2	1	1	2	0	0	0
407	57	1	2	1	1	2	0	0	0
408	62	2	1	1	1	2	0	0	0
409	53	2	1	1	2	2	0	0	0
410	67	2	2	1	1	2	0	0	0
411	65	1	1	1	1	2	0	0	0
412	61	2	1	1	1	2	0	0	0
413	64	1	3	1	1	2	0	0	0
414	57	2	1	1	2	3	0	0	0
415	42	1	2	1	1	3	0	0	0
416	61	1	2	1	1	2	0	0	0
417	55	1	2	1	2	2	0	0	0

418	41	1	1	1	2	2	0	0	0
419	67	1	1	1	2	2	0	0	0
420	55	2	1	1	2	2	1	0	1
421	60	1	2	1	1	2	0	0	0
422	79	1	1	1	1	1	1	0	0
423	49	2	1	2	1	2	0	0	0
424	73	1	1	1	2	2	0	0	0
425	65	2	2	1	1	1	0	0	0
426	65	1	1	1	1	2	0	0	0
427	61	2	1	1	2	2	0	0	0
428	57	1	2	1	1	2	0	0	0
429	48	2	1	1	1	2	0	0	0
430	70	1	2	1	1	2	0	0	0
431	44	2	1	1	2	2	0	0	0
432	58	2	2	1	1	2	0	0	0
433	43	1	1	1	2	2	0	0	1
434	69	1	3	1	1	1	0	0	0
435	44	1	2	1	2	2	0	0	0
436	53	1	2	2	1	3	0	0	0
437	63	1	1	1	2	2	0	0	0
438	63	2	1	1	2	2	0	0	0
439	69	1	2	1	2	4	0	0	0
440	55	2	2	1	2	2	0	0	0
441	64	1	1	1	2	2	0	0	0
442	59	2	1	1	2	2	0	0	0

443	51	2	1	1	2	2	0	0	0
444	62	1	2	1	2	1	1	0	1
445	64	2	2	1	2	2	0	0	0
446	30	1	2	1	2	2	0	0	0
447	75	1	1	1	2	3	0	0	0
448	48	2	2	1	2	3	0	0	0
449	45	2	2	1	2	3	1	0	0
450	76	2	2	1	1	1	0	0	0
451	63	2	1	1	1	2	0	0	0
452	63	2	1	1	2	1	0	0	0
453	45	2	2	1	2	2	0	0	0
454	60	2	2	1	2	2	0	0	0
455	59	1	2	1	1	2	1	1	0
456	37	2	1	1	1	2	0	0	0
457	63	2	1	1	2	3	0	0	0
458	63	2	1	2	2	2	0	0	0
459	56	1	2	1	2	3	1	0	0
460	46	2	2	1	1	2	1	0	0
461	63	1	2	1	1	1	0	0	0
462	45	1	1	1	1	2	0	0	0
463	67	2	2	1	1	4	1	0	1
464	40	2	3	1	1	1	0	0	0
465	62	2	1	1	2	2	0	0	0
466	30	2	2	1	2	1	0	0	0
467	57	1	1	2	1	3	0	0	0

468	58	2	2	1	1	4	0	0	0
469	36	1	2	1	2	1	1	1	1
470	52	2	2	1	2	1	0	0	0
471	54	1	2	1	1	2	1	0	1
472	60	1	2	1	2	2	0	0	0
473	61	1	2	1	2	2	0	0	1
474	72	1	2	1	2	1	0	0	0
475	71	1	2	1	1	1	0	0	0
476	56	2	1	1	1	2	0	0	0
477	66	2	1	1	2	3	0	0	1
478	69	2	2	1	2	3	0	0	0
479	50	2	2	1	2	1	1	0	0
480	63	1	2	1	2	2	1	1	0
481	55	1	2	1	2	3	0	0	1
482	46	1	2	1	1	2	1	0	0
483	54	2	2	2	2	1	0	1	1
484	45	2	1	1	2	2	0	0	0
485	62	1	3	1	1	3	0	0	0
486	18	2	2	2	1	1	0	0	0
487	33	2	1	1	2	4	0	0	0
488	70	1	1	1	2	2	0	1	0
489	52	1	1	2	2	3	0	0	0
490	65	1	2	1	1	2	1	0	1
491	71	1	2	2	1	1	1	0	1
492	61	1	2	1	1	2	1	0	0

493	55	1	2	1	2	3	0	0	0
494	57	1	3	1	1	4	1	0	0
495	57	1	2	1	2	2	0	0	0
496	72	2	1	1	2	2	0	0	0
497	58	1	2	1	2	2	1	0	0
498	46	2	2	1	2	4	0	0	0
499	59	2	1	1	2	3	0	0	0
500	52	1	2	1	2	2	0	0	0
501	65	1	3	1	1	3	0	0	1
502	34	1	3	1	2	2	0	0	1
503	49	1	2	1	1	1	0	0	0
504	45	2	2	1	2	2	0	0	0
505	79	1	1	1	1	3	0	0	0
506	61	2	2	1	1	2	0	0	1
507	61	2	1	1	1	2	0	1	0
508	55	1	1	1	1	2	1	0	0
509	52	2	2	2	1	1	0	1	0
510	54	1	2	1	1	2	0	0	0
511	41	2	2	1	2	1	0	0	0
512	55	1	1	1	1	2	0	0	0
513	61	1	2	1	2	2	0	0	0
514	72	1	1	1	2	2	0	0	0
515	57	2	2	1	2	1	1	1	0
516	62	1	2	1	2	2	0	0	0
517	75	1	1	1	2	2	0	0	0

518	59	1	2	1	2	3	0	0	0
519	50	2	1	1	2	2	0	0	0
520	51	2	2	1	1	4	0	0	0
521	64	2	2	1	1	2	0	0	0
522	70	1	1	1	2	2	1	0	0
523	54	2	2	1	2	2	0	0	0
524	60	1	2	1	1	2	0	1	0
525	62	1	2	1	2	2	0	0	0
526	50	2	2	1	1	2	1	0	0
527	69	1	1	1	1	1	0	0	0
528	69	1	2	1	1	2	0	0	0
529	70	2	1	1	2	2	0	0	0
530	74	2	2	1	1	3	0	0	0
531	42	2	2	1	2	2	0	1	0
532	56	1	2	1	1	2	0	0	0
533	71	1	1	1	2	1	0	0	0
534	62	1	2	1	2	2	1	0	0
535	67	1	2	1	1	2	0	0	0
536	24	2	3	2	2	3	0	0	0
537	54	2	1	1	2	3	0	0	0
538	67	2	1	1	2	2	0	1	1
539	37	1	3	1	2	1	1	0	0
540	73	2	1	1	2	1	0	0	0
541	55	1	3	1	1	2	1	1	0
542	50	1	3	1	1	2	0	0	0

543	53	2	1	1	2	2	0	0	0
544	44	1	3	1	1	2	0	0	0
545	57	1	1	1	2	2	0	0	0
546	54	1	1	1	2	2	0	0	0
547	56	1	1	1	2	3	0	0	1
548	51	2	2	1	2	2	0	0	0
549	51	2	2	1	2	2	0	0	0
550	49	2	2	1	1	2	0	0	1
551	75	1	1	1	2	1	0	0	0
552	61	1	2	1	1	2	1	0	1
553	56	1	2	1	2	2	1	0	1
554	56	1	2	1	1	1	0	0	0
555	46	1	2	1	2	1	1	0	1
556	80	2	1	1	2	1	0	0	0
557	51	1	2	1	1	4	0	0	0
558	46	1	3	1	1	1	0	1	0
559	45	1	3	1	2	2	1	1	0
560	42	2	1	1	2	2	0	0	1
561	49	1	2	1	2	1	0	1	0
562	60	1	2	1	1	4	0	0	0
563	46	1	2	1	1	2	0	0	0
564	53	1	2	1	2	2	0	0	0
565	76	1	1	1	2	1	1	0	0
566	68	1	3	1	1	2	1	0	0
567	63	2	1	1	2	2	1	0	0

568	48	2	3	1	1	1	1	0	0
569	59	1	3	1	1	2	1	1	0
570	50	1	1	1	1	2	0	0	0
571	65	2	2	1	1	2	0	0	0
572	72	2	1	1	1	1	1	0	0
573	68	2	1	1	1	1	0	0	0
574	75	1	1	1	1	1	0	0	0
575	37	2	2	1	1	3	1	0	1
576	47	2	2	1	2	1	0	0	1
577	65	1	2	1	2	2	0	0	0
578	52	1	2	1	2	1	0	0	1
579	30	2	2	1	2	2	1	0	0
580	50	1	2	1	2	2	0	1	1
581	71	1	1	1	1	3	1	0	1
582	79	2	3	1	1	1	1	0	0
583	70	1	2	1	2	4	1	0	0
584	48	2	2	1	2	3	0	0	1
585	75	1	1	1	1	1	1	0	0
586	62	1	1	1	2	1	0	0	0
587	55	1	2	1	2	2	1	0	0
588	53	1	2	1	1	3	0	0	0
589	46	1	2	1	2	1	0	0	1
590	78	2	2	1	1	4	0	0	0
591	67	2	2	1	1	2	0	0	0
592	50	1	1	1	2	3	0	0	0

593	66	2	2	1	2	2	0	0	0
594	55	2	1	1	1	2	0	0	1
595	54	2	1	1	2	3	1	0	1
596	63	1	1	1	1	3	1	0	0
597	32	2	3	1	1	1	0	0	1
598	63	1	2	1	1	1	0	0	0
599	68	2	1	1	2	2	0	0	0
600	38	2	2	1	2	2	0	0	0
601	77	1	1	1	2	2	0	0	0
602	73	2	1	1	1	2	0	0	0
603	64	1	2	1	1	2	0	0	0
604	34	1	2	1	1	2	0	0	0
605	45	2	1	1	1	2	0	0	0
606	73	1	1	1	2	1	1	0	0
607	62	1	2	1	2	2	0	1	0
608	69	1	2	1	1	1	0	0	1
609	36	2	3	2	1	1	0	0	0
610	67	2	1	1	1	2	1	0	0
611	75	1	1	1	2	2	1	0	0
612	30	1	2	1	2	1	0	1	0
613	46	2	1	1	2	1	0	0	0
614	30	2	2	1	2	2	0	0	0
615	54	1	1	1	2	1	0	0	0
616	59	1	2	1	2	1	0	0	1
617	66	1	2	1	2	1	0	0	0

618	56	1	3	1	1	3	1	0	0
619	51	1	2	1	1	3	1	0	0
620	30	2	2	1	2	3	0	0	0
621	59	2	2	1	2	4	0	0	0
622	52	2	1	1	2	2	1	0	0
623	48	1	2	1	2	2	0	0	0
624	49	2	2	1	2	4	0	0	0
625	55	1	3	1	1	2	1	0	0
626	66	1	1	1	1	2	0	0	0
627	80	2	1	1	2	1	0	0	0
628	43	2	2	1	2	2	0	0	0
629	54	1	2	1	1	1	0	0	0
630	31	1	3	1	2	1	0	0	0
631	62	1	1	1	2	1	0	0	0
632	55	1	2	1	2	1	0	0	0
633	65	1	1	1	2	2	0	0	0
634	68	1	1	1	2	2	0	0	0
635	52	1	1	1	2	3	0	0	0
636	48	2	2	1	2	4	0	0	0
637	63	1	2	1	2	2	0	1	0
638	48	2	1	1	2	4	0	1	0
639	65	2	1	1	2	1	1	0	0
640	40	1	3	1	1	3	0	0	0
641	57	1	3	1	2	1	0	1	1
642	56	1	2	1	2	2	0	0	0

643	53	2	3	1	1	1	1	0	0
644	50	2	1	1	2	3	0	0	0
645	32	2	3	1	2	3	1	1	0
646	58	1	2	1	2	1	0	0	0
647	43	2	2	1	2	2	1	0	0
648	72	1	3	1	1	2	1	0	1
649	55	1	2	1	2	2	1	0	1
650	53	2	1	1	1	2	0	0	0
651	62	1	3	1	1	2	0	0	0
652	54	1	3	1	2	1	0	0	0
653	54	1	2	1	2	2	0	0	1
654	31	2	2	1	2	1	0	0	0
655	55	2	2	1	2	2	0	0	0
656	64	2	1	1	2	3	0	0	0
657	36	1	2	1	2	3	0	0	0
658	49	1	2	1	2	2	1	0	1
659	61	1	1	1	2	2	0	0	0
660	24	2	3	2	1	1	0	0	1
661	34	1	2	1	2	1	0	0	1
662	59	1	2	1	2	2	0	0	0
663	73	1	2	1	2	2	0	0	0
664	75	1	1	1	2	2	1	0	0
665	50	2	2	1	2	1	0	0	0
666	59	1	1	1	2	1	0	0	0
667	71	1	1	1	2	2	0	0	1

668	61	1	3	1	1	2	1	0	0
669	67	2	2	1	2	2	0	0	0
670	68	1	2	1	1	1	0	1	0
671	45	2	2	1	2	2	0	0	0
672	68	2	1	1	2	1	0	0	0
673	47	1	1	1	2	1	0	0	0
674	55	2	2	1	2	4	0	0	0
675	56	1	1	1	2	2	0	0	0
676	48	2	1	1	2	1	0	0	0
677	59	1	2	1	1	2	0	1	0
678	76	2	2	1	2	3	0	0	0
679	63	2	2	1	2	3	1	0	0
680	55	1	2	1	2	2	0	0	0
681	58	1	2	1	2	2	0	0	0
682	53	2	2	1	2	1	0	0	0
683	63	2	2	1	2	1	0	0	0
684	80	1	1	1	2	2	1	0	0
685	56	1	2	1	1	2	0	0	0
686	65	1	2	1	2	2	0	0	0
687	44	2	1	1	2	2	0	0	0
688	57	2	1	1	2	2	1	1	0
689	67	1	1	1	1	2	0	0	0
690	46	2	1	1	2	1	0	0	0
691	61	2	2	1	2	1	0	1	0
692	56	2	2	1	1	1	0	0	0

693	72	1	1	1	2	1	0	0	0
694	31	1	3	1	1	1	0	0	0
695	57	2	2	1	1	1	1	1	1
696	41	1	3	1	1	1	0	0	0
697	61	1	2	1	2	3	0	0	0
698	60	1	2	1	2	1	0	1	0
699	57	2	2	1	2	1	1	1	0
700	46	1	1	1	2	2	1	1	1
701	69	1	1	1	2	2	0	0	0
702	42	2	2	1	2	2	0	0	0
703	64	1	2	1	2	2	0	1	0
704	56	2	1	1	2	1	0	0	0
705	71	1	1	1	2	2	0	0	0
706	74	1	2	1	1	2	0	0	0
707	57	1	3	1	2	1	0	0	0
708	55	1	2	1	2	2	0	0	0
709	67	1	1	1	1	3	0	0	0
710	50	2	1	1	2	2	0	0	0
711	51	2	1	1	2	3	0	0	0
712	53	1	1	1	2	1	1	1	0
713	52	1	2	1	2	4	0	0	0
714	61	2	1	1	2	2	0	0	0
715	61	1	2	1	1	3	0	0	0
716	50	1	1	1	2	1	0	0	1
717	65	2	1	1	2	2	0	0	0

718	55	1	1	1	1	2	0	0	1
719	46	1	2	1	2	4	0	0	0
720	62	1	2	1	1	2	0	0	0
721	47	2	2	1	2	2	0	0	0
722	49	2	2	1	2	2	0	0	1
723	56	1	3	1	2	2	0	0	0
724	46	2	3	1	1	2	0	0	0
725	52	2	2	1	2	2	0	0	0
726	68	1	3	1	1	1	0	0	1
727	66	1	1	1	2	2	0	0	0
728	51	2	2	1	2	2	0	0	0
729	65	2	1	1	2	3	0	0	0
730	18	1	2	1	2	1	1	1	0
731	66	1	1	1	2	2	0	0	1
732	59	1	2	1	2	3	0	0	1
733	52	2	1	1	2	1	0	0	0
734	83	1	2	1	1	2	1	0	0
735	57	2	2	1	2	2	0	0	0
736	60	2	2	1	2	2	0	0	0
737	66	1	2	1	1	1	0	0	0
738	72	2	1	1	2	2	0	0	0
739	49	1	2	1	2	2	0	0	0
740	83	1	1	1	1	3	1	0	1
741	58	1	2	1	2	3	1	0	1
742	68	1	2	1	2	2	1	0	0

743	55	1	3	2	2	3	0	0	1
744	73	1	2	1	2	1	0	0	0
745	53	2	1	1	2	2	0	0	0
746	74	2	2	1	1	1	1	1	0
747	40	1	3	1	2	2	0	0	0
748	66	2	2	1	2	2	0	0	0
749	62	1	2	1	1	1	0	0	0
750	61	2	1	1	2	2	1	0	0
751	33	2	2	1	2	2	0	0	0
752	49	1	1	2	2	3	0	0	0
753	43	2	2	1	2	2	0	1	0
754	69	1	2	2	2	3	0	0	0
755	50	2	3	1	1	2	1	1	0
756	54	2	3	1	1	2	1	0	0
757	50	2	2	1	2	2	0	0	0
758	51	2	3	1	2	3	0	0	1
759	49	2	2	1	2	3	1	0	0
760	53	1	2	1	2	2	1	1	1
761	70	2	1	1	1	3	0	0	0
762	73	1	1	1	1	2	0	1	1
763	65	2	3	1	1	4	0	1	0
764	65	1	1	1	1	2	1	0	0
765	48	1	3	1	1	2	0	0	0
766	63	1	2	2	2	4	1	0	0
767	53	1	2	1	2	3	0	0	0

768	64	2	1	1	2	1	0	0	0
769	52	2	2	1	1	2	1	0	0
770	61	2	1	1	2	2	1	0	0
771	69	2	2	1	2	2	0	0	1
772	40	1	3	1	1	3	0	0	0
773	48	2	2	2	1	2	0	0	0
774	55	2	1	1	2	4	0	0	0
775	46	1	1	1	1	2	1	0	1
776	34	2	2	1	2	2	0	0	0
777	53	2	2	1	1	2	1	0	1
778	51	1	3	1	1	2	0	0	0
779	70	2	1	1	1	2	1	0	0
780	59	2	2	1	1	2	0	1	1
781	45	2	1	1	2	4	0	0	0
782	49	2	1	1	2	3	1	0	1
783	53	2	1	1	1	3	0	1	0
784	45	2	1	1	2	2	0	0	0
785	82	1	1	1	2	2	0	0	0
786	60	1	1	1	1	1	1	0	0
787	67	1	1	1	1	2	1	0	0
788	63	2	2	1	1	2	1	0	1
789	42	1	2	1	1	1	1	0	0
790	47	2	2	1	1	1	0	1	0
791	66	2	1	1	1	2	0	0	0
792	53	1	2	1	1	1	1	0	0

793	45	2	1	1	1	2	0	0	0
794	65	1	1	1	1	1	1	0	1
795	36	2	2	1	2	2	0	0	1
796	37	2	3	1	1	1	0	0	0
797	64	1	2	1	2	1	0	0	0
798	31	2	2	1	2	1	0	0	0

number	Liver metastasis	Metastasis(1yes; 0no)	Metastasis number（0 none；1 one；2 two；3 three and above）	head and neck cancer	Liver and Cholecyst cancer	gastric cancer	pancreatic cancer	lung cancer	breast cancer	colorectal cancer
1	0	1	1	1	0	0	0	0	0	0
2	0	1	1	0	0	0	1	0	0	0
3	0	1	1	0	0	0	0	0	0	0
4	0	1	3	0	0	1	0	0	0	0
5	1	1	1	0	0	0	0	0	0	1
6	0	1	2	0	0	0	0	1	0	0
7	1	1	2	0	0	0	0	1	0	0
8	1	1	2	0	1	0	0	0	0	0
9	0	1	1	0	0	0	0	1	0	0
10	0	1	1	0	0	0	0	0	0	0
11	1	1	3	1	0	0	0	0	0	0
12	0	1	2	0	0	0	0	1	0	0
13	0	1	2	0	0	0	0	1	0	0

14	1	1	3	0	0	0	0	1	0	0
15	0	0	0	0	0	0	0	1	0	0
16	0	0	0	1	0	0	0	0	0	0
17	1	1	1	0	0	0	0	1	0	0
18	0	0	0	1	0	0	0	0	0	0
19	1	1	2	0	0	0	0	0	0	0
20	0	1	1	0	0	0	0	0	1	0
21	1	1	2	0	0	0	0	1	0	0
22	0	1	1	0	0	0	0	0	0	0
23	0	1	1	1	0	0	0	0	0	0
24	0	0	0	0	0	0	0	0	0	0
25	1	1	3	0	0	0	0	1	0	0
26	0	1	3	0	0	0	0	0	0	0
27	0	1	1	0	0	0	0	1	0	0
28	1	1	1	0	0	0	0	0	1	0
29	0	1	2	1	0	0	0	0	0	0
30	1	1	3	0	0	0	0	0	1	0
31	0	0	0	0	0	0	0	0	0	0
32	1	1	2	0	0	0	0	0	0	1
33	1	1	3	0	0	0	0	0	0	1
34	0	1	2	0	0	1	0	0	0	0
35	0	1	2	1	0	0	0	0	0	0
36	0	1	1	0	0	0	0	0	0	0
37	1	1	3	0	0	0	0	0	1	0
38	1	1	2	0	0	0	0	0	0	1

39	0	1	1	0	1	0	0	0	0	0
40	0	1	3	0	0	0	0	0	0	1
41	0	0	0	0	0	0	0	0	0	0
42	0	1	1	0	0	0	0	0	0	0
43	0	1	1	0	0	1	0	0	0	0
44	0	0	0	0	1	0	0	0	0	0
45	0	1	2	0	0	0	0	0	0	1
46	0	0	0	0	0	0	0	1	0	0
47	0	0	0	0	0	0	1	0	0	0
48	0	1	1	0	0	0	0	0	0	0
49	0	0	0	0	0	0	0	1	0	0
50	1	1	3	0	0	0	0	0	0	0
51	0	1	1	0	0	0	0	1	0	0
52	0	1	2	1	0	0	0	0	0	0
53	1	1	3	0	0	0	0	1	0	0
54	0	1	1	0	0	0	0	0	0	0
55	0	0	0	0	0	0	0	1	0	0
56	0	1	2	0	0	0	0	1	0	0
57	0	1	3	0	0	0	0	0	0	0
58	1	1	3	0	0	0	0	0	0	0
59	1	1	1	0	0	0	0	0	0	1
60	0	1	3	0	0	0	0	0	0	1
61	0	0	0	0	0	0	0	0	0	0
62	1	1	3	0	0	0	0	0	0	0
63	1	1	3	0	0	0	0	0	0	0

64	0	0	0	1	0	0	0	0	0	0
65	0	1	2	1	0	0	0	0	0	0
66	0	0	0	0	0	0	0	0	0	0
67	0	1	1	0	0	0	0	0	0	1
68	1	1	3	0	0	0	0	0	0	0
69	0	0	0	1	0	0	0	0	0	0
70	0	1	1	0	0	0	0	0	0	0
71	0	1	1	0	0	0	0	1	0	0
72	0	1	2	0	0	0	0	0	0	0
73	0	1	2	0	0	0	0	1	0	0
74	0	1	1	0	1	0	0	0	0	0
75	1	1	2	0	0	0	0	0	0	0
76	0	1	1	0	0	0	0	0	0	0
77	0	1	1	0	0	0	0	0	0	0
78	0	0	0	0	0	0	0	0	0	0
79	0	1	1	0	0	0	0	1	0	0
80	1	1	2	0	0	0	0	0	0	0
81	0	1	1	0	0	0	0	1	0	0
82	1	1	2	0	0	0	0	0	0	1
83	1	1	2	0	0	0	0	0	0	0
84	1	1	1	0	0	0	0	1	0	0
85	1	1	2	0	0	0	1	0	0	0
86	0	1	1	0	0	0	0	0	0	1
87	0	1	1	0	0	0	0	0	0	0
88	1	1	3	0	0	0	0	1	0	0

89	0	1	1	0	0	0	0	1	0	0
90	0	1	3	0	0	0	0	0	1	0
91	0	1	3	0	0	0	0	1	0	0
92	0	1	1	0	0	0	0	0	0	0
93	0	1	2	0	1	0	0	0	0	0
94	1	1	3	0	0	0	0	1	0	0
95	0	1	1	0	1	0	0	0	0	0
96	1	1	2	0	0	0	0	1	0	0
97	0	1	1	0	0	1	0	0	0	0
98	0	1	1	0	0	0	0	1	0	0
99	0	1	2	1	0	0	0	0	0	0
100	0	1	3	0	0	0	0	1	0	0
101	0	1	2	0	1	0	0	0	0	0
102	0	1	1	1	0	0	0	0	0	0
103	0	1	3	0	0	0	0	1	0	0
104	1	1	3	0	0	0	1	0	0	0
105	0	1	1	0	1	0	0	0	0	0
106	0	1	1	0	0	0	0	1	0	0
107	0	1	1	0	0	0	0	0	0	1
108	1	1	3	0	0	0	0	0	1	0
109	1	1	2	0	0	0	0	1	0	0
110	0	1	1	0	0	0	0	1	0	0
111	1	1	3	0	0	0	0	0	0	1
112	0	1	1	0	0	0	0	0	0	1
113	0	1	2	0	0	1	0	0	0	0

114	0	1	1	1	0	0	0	0	0	0
115	1	1	3	0	0	0	0	0	0	1
116	0	1	1	0	0	0	0	1	0	0
117	0	1	3	1	0	0	0	0	0	0
118	1	1	2	0	0	0	0	1	0	0
119	0	1	3	0	0	0	0	1	0	0
120	0	1	2	0	1	0	0	0	0	0
121	0	1	3	0	0	0	0	1	0	0
122	0	1	3	0	0	0	0	0	0	0
123	0	1	2	0	0	0	0	0	1	0
124	0	0	0	0	0	0	0	0	0	0
125	0	1	1	0	0	0	0	0	0	0
126	1	1	2	0	0	0	0	0	0	1
127	0	1	1	0	0	0	0	1	0	0
128	1	1	3	0	0	0	0	0	1	0
129	0	0	0	0	0	0	0	0	0	0
130	1	1	1	0	0	0	0	0	1	0
131	0	0	0	0	1	0	0	0	0	0
132	0	0	0	0	0	0	0	1	0	0
133	0	1	2	0	0	0	0	1	0	0
134	0	1	2	0	0	0	0	0	0	0
135	0	0	0	0	0	0	0	0	0	1
136	0	1	1	0	0	0	0	1	0	0
137	0	1	1	0	0	0	0	1	0	0
138	1	1	1	0	0	0	0	0	0	0

139	0	1	1	1	0	0	0	0	0	0
140	0	1	2	0	0	0	0	0	0	0
141	0	0	0	0	0	0	0	0	0	0
142	0	0	0	0	0	0	0	0	0	0
143	1	1	3	0	0	0	0	0	0	1
144	0	1	2	0	0	1	0	0	0	0
145	0	1	2	0	0	0	0	1	0	0
146	0	1	1	1	0	0	0	0	0	0
147	0	1	2	1	0	0	0	0	0	0
148	0	1	2	0	0	0	0	1	0	0
149	0	0	0	0	0	0	1	0	0	0
150	0	0	0	0	0	0	0	0	0	0
151	0	0	0	0	0	0	0	1	0	0
152	0	0	0	0	0	0	1	0	0	0
153	0	1	2	0	0	0	0	1	0	0
154	1	1	1	0	0	0	0	1	0	0
155	0	1	1	0	0	0	0	0	0	0
156	0	1	1	0	0	0	0	0	0	0
157	0	0	0	0	0	0	0	1	0	0
158	0	0	0	0	0	0	0	0	0	0
159	0	1	2	0	0	0	0	0	0	1
160	1	1	2	0	0	0	0	0	0	0
161	0	0	0	0	0	0	1	0	0	0
162	0	1	2	0	0	0	0	0	0	0
163	0	0	0	0	0	0	0	0	0	0

164	0	1	1	1	0	0	0	0	0	0
165	0	1	1	0	1	0	0	0	0	0
166	0	0	0	0	1	0	0	0	0	0
167	1	1	3	0	0	0	0	0	0	1
168	0	1	1	0	0	0	0	1	0	0
169	0	1	1	0	0	0	0	0	0	0
170	0	0	0	0	0	0	0	0	0	0
171	0	1	3	0	0	0	0	0	0	1
172	0	0	0	1	0	0	0	0	0	0
173	0	1	1	0	0	0	0	0	0	0
174	0	0	0	0	0	0	0	0	1	0
175	0	1	3	0	0	0	0	0	1	0
176	1	1	2	0	0	0	0	1	0	0
177	0	0	0	0	0	0	0	0	0	0
178	0	1	2	0	0	0	0	0	0	0
179	0	1	1	0	0	0	0	0	0	0
180	0	0	0	1	0	0	0	0	0	0
181	0	1	2	0	0	0	0	0	0	0
182	0	0	0	0	0	0	0	1	0	0
183	0	1	1	0	0	0	0	0	0	0
184	0	0	0	0	0	0	0	0	0	0
185	0	0	0	0	0	0	0	0	1	0
186	0	0	0	0	0	0	0	0	0	0
187	0	0	0	0	0	0	0	0	0	0
188	1	1	3	0	0	0	0	1	0	0

189	0	0	0	0	0	0	0	1	0	0
190	0	1	2	0	0	0	0	0	0	0
191	0	1	1	0	0	0	0	1	0	0
192	1	1	2	0	0	0	0	0	0	1
193	0	1	1	0	0	0	0	0	1	0
194	0	1	3	0	0	0	0	0	0	0
195	0	1	1	0	0	0	0	1	0	0
196	0	1	1	0	1	0	0	0	0	0
197	1	1	2	1	0	0	0	0	0	0
198	0	1	1	0	0	0	0	0	0	1
199	1	1	1	0	0	0	1	0	0	0
200	0	1	1	0	0	0	0	1	0	0
201	0	1	1	0	0	0	0	0	0	1
202	0	1	3	0	0	0	0	1	0	0
203	0	0	0	0	0	0	0	1	0	0
204	0	1	3	0	0	0	0	1	0	0
205	0	0	0	0	0	0	0	0	0	0
206	0	0	0	0	0	0	0	1	0	0
207	0	0	0	0	0	0	0	0	0	1
208	0	0	0	0	0	0	0	1	0	0
209	0	0	0	0	0	0	0	1	0	0
210	0	0	0	0	0	0	0	0	0	1
211	1	1	3	0	0	0	0	1	0	0
212	0	1	2	0	0	0	0	1	0	0
213	0	0	0	0	1	0	0	0	0	0

214	0	0	0	0	0	0	0	1	0	0
215	0	0	0	0	0	0	0	0	0	1
216	0	1	1	0	0	0	0	0	0	0
217	0	0	0	0	0	0	0	0	0	1
218	0	1	1	0	0	0	0	1	0	0
219	1	1	2	0	0	0	0	1	0	0
220	0	0	0	0	0	0	0	0	0	0
221	0	1	2	0	0	0	1	0	0	0
222	0	1	3	0	0	0	0	1	0	0
223	0	1	1	0	0	0	1	0	0	0
224	0	1	2	1	0	0	0	0	0	0
225	1	1	3	0	0	0	0	1	0	0
226	0	0	0	0	0	0	0	1	0	0
227	0	0	0	0	0	0	0	0	1	0
228	0	1	2	0	0	0	0	1	0	0
229	0	0	0	0	0	0	0	0	0	0
230	1	1	2	0	0	0	0	0	0	0
231	0	1	1	0	0	0	0	0	0	0
232	1	1	3	0	0	0	0	1	0	0
233	1	1	1	0	0	0	0	0	0	1
234	0	1	2	0	0	0	0	0	0	0
235	0	1	1	0	0	0	0	0	0	0
236	0	1	1	0	0	0	0	0	0	0
237	1	1	3	0	0	0	0	0	0	0
238	1	1	2	0	0	1	0	0	0	0

239	0	1	2	0	0	0	0	1	0	0
240	0	0	0	0	0	0	0	0	0	0
241	1	1	3	0	0	0	0	1	0	0
242	1	1	3	0	0	0	0	0	0	0
243	0	0	0	0	0	0	0	0	0	0
244	0	0	0	0	0	0	0	0	0	1
245	0	0	0	0	1	0	0	0	0	0
246	0	0	0	0	0	0	0	1	0	0
247	0	0	0	1	0	0	0	0	0	0
248	0	0	0	0	0	0	0	1	0	0
249	0	0	0	1	0	0	0	0	0	0
250	0	1	1	0	0	0	0	0	0	1
251	0	1	1	0	0	0	0	1	0	0
252	1	1	2	0	0	0	0	1	0	0
253	1	1	3	0	0	0	1	0	0	0
254	0	1	2	0	0	0	0	0	0	0
255	0	0	0	0	0	0	0	1	0	0
256	1	1	3	0	0	0	0	0	0	0
257	0	1	1	0	0	0	0	1	0	0
258	0	1	1	0	0	0	0	0	0	0
259	0	1	1	0	0	0	0	0	0	0
260	0	1	1	0	1	0	0	0	0	0
261	0	0	0	0	0	0	0	0	0	0
262	1	1	2	0	0	0	0	0	0	1
263	0	0	0	0	1	0	0	0	0	0

264	1	1	3	0	0	0	0	1	0	0
265	0	1	2	0	0	0	0	0	0	0
266	0	1	2	0	0	0	0	1	0	0
267	1	1	1	0	1	0	0	0	0	0
268	0	0	0	0	0	0	0	1	0	0
269	0	0	0	0	0	0	0	1	0	0
270	0	1	1	0	0	0	0	1	0	0
271	0	1	1	1	0	0	0	0	0	0
272	1	1	3	0	0	0	0	0	0	0
273	1	1	1	0	0	0	0	0	1	0
274	0	1	1	0	0	0	0	0	0	1
275	0	1	2	0	0	0	0	1	0	0
276	0	1	2	1	0	0	0	0	0	0
277	1	1	1	0	0	0	0	0	0	0
278	0	1	1	0	0	0	0	0	0	0
279	0	0	0	0	0	0	0	0	0	1
280	0	0	0	0	0	0	0	1	0	0
281	0	0	0	0	1	0	0	0	0	0
282	1	1	1	0	1	0	0	0	0	0
283	1	1	2	0	0	0	1	0	0	0
284	0	0	0	0	0	1	0	0	0	0
285	1	1	2	0	0	0	0	0	0	0
286	0	0	0	0	0	0	0	0	0	0
287	0	1	2	0	0	0	0	1	0	0
288	1	1	2	0	1	0	0	0	0	0

289	0	1	3	0	0	0	0	1	0	0
290	0	1	1	0	1	0	0	0	0	0
291	0	1	1	0	0	0	0	0	0	1
292	0	1	2	0	1	0	0	0	0	0
293	0	1	1	0	0	0	0	1	0	0
294	0	1	1	0	0	0	0	0	1	0
295	0	0	0	0	0	0	0	0	0	0
296	1	1	3	0	0	0	0	1	0	0
297	0	1	2	0	0	0	0	0	0	1
298	1	1	3	0	0	0	0	0	0	0
299	0	1	2	0	0	0	0	1	0	0
300	0	0	0	0	0	0	0	1	0	0
301	0	0	0	0	0	0	0	0	0	0
302	0	1	3	0	0	0	0	0	0	0
303	0	0	0	0	0	0	0	1	0	0
304	1	1	2	0	0	0	0	0	0	0
305	0	0	0	0	0	0	0	0	0	0
306	0	0	0	0	1	0	0	0	0	0
307	0	0	0	0	0	0	0	1	0	0
308	0	0	0	0	0	1	0	0	0	0
309	0	0	0	0	0	0	0	1	0	0
310	0	0	0	0	0	0	0	0	0	1
311	0	1	1	0	0	0	0	1	0	0
312	0	1	3	0	0	0	0	1	0	0
313	1	1	3	0	0	0	0	0	1	0

314	0	0	0	1	0	0	0	0	0	0
315	0	1	2	1	0	0	0	0	0	0
316	0	1	3	0	0	0	0	0	0	0
317	0	1	2	0	0	0	0	1	0	0
318	0	1	3	0	0	0	0	0	1	0
319	0	0	0	1	0	0	0	0	0	0
320	0	0	0	0	0	0	0	1	0	0
321	0	0	0	0	0	0	0	0	0	0
322	0	0	0	0	0	0	0	0	0	1
323	0	0	0	0	0	0	0	0	0	1
324	0	1	3	0	0	0	0	0	0	0
325	0	0	0	0	0	0	0	1	0	0
326	0	1	1	0	0	0	0	1	0	0
327	0	0	0	0	0	0	0	0	0	1
328	0	1	1	0	1	0	0	0	0	0
329	0	0	0	0	1	0	0	0	0	0
330	0	0	0	0	0	1	0	0	0	0
331	1	1	3	0	0	0	0	1	0	0
332	1	1	2	0	0	0	1	0	0	0
333	0	0	0	1	0	0	0	0	0	0
334	0	0	0	0	0	0	1	0	0	0
335	0	0	0	0	0	0	0	1	0	0
336	0	0	0	0	0	0	0	1	0	0
337	0	0	0	0	0	0	0	1	0	0
338	0	0	0	0	0	0	0	0	0	0

339	0	0	0	0	0	1	0	0	0	0
340	0	1	1	0	0	0	0	0	0	0
341	0	1	2	0	0	0	0	0	0	1
342	0	0	0	0	0	0	0	1	0	0
343	0	0	0	0	1	0	0	0	0	0
344	0	1	1	0	0	0	0	1	0	0
345	0	0	0	0	0	0	0	0	0	0
346	0	0	0	0	0	0	0	0	0	0
347	0	0	0	1	0	0	0	0	0	0
348	0	0	0	0	0	0	0	0	0	0
349	0	0	0	0	0	0	0	0	0	0
350	0	0	0	0	0	0	0	0	1	0
351	1	1	3	0	0	0	0	0	0	0
352	0	0	0	0	0	0	0	0	0	0
353	0	0	0	0	0	0	0	0	0	1
354	0	1	1	0	0	0	0	1	0	0
355	0	0	0	0	0	0	0	0	0	0
356	0	0	0	0	0	0	0	0	0	1
357	0	0	0	0	0	0	0	0	0	0
358	0	0	0	0	0	0	1	0	0	0
359	0	0	0	0	0	0	0	0	0	0
360	0	0	0	0	0	0	0	0	0	0
361	0	0	0	0	0	0	0	0	1	0
362	0	0	0	0	0	0	1	0	0	0
363	0	0	0	0	0	0	0	1	0	0

364	0	0	0	0	0	0	0	1	0	0
365	0	0	0	0	0	0	0	0	0	0
366	0	0	0	0	0	0	0	0	0	0
367	0	0	0	0	0	0	0	0	0	0
368	0	0	0	0	0	0	0	1	0	0
369	0	1	1	0	0	0	0	0	0	1
370	0	1	2	0	0	0	0	1	0	0
371	0	0	0	0	0	0	0	0	1	0
372	0	0	0	0	0	0	0	0	0	0
373	0	0	0	0	0	0	0	0	0	1
374	0	0	0	0	0	0	0	0	0	0
375	0	1	1	0	0	1	0	0	0	0
376	0	0	0	1	0	0	0	0	0	0
377	0	1	2	0	0	0	0	0	0	0
378	0	1	1	0	0	0	0	1	0	0
379	0	0	0	0	0	0	0	0	0	1
380	0	0	0	0	0	0	0	1	0	0
381	0	1	2	0	0	0	0	0	0	0
382	0	1	1	0	0	0	0	1	0	0
383	0	1	2	0	0	0	0	1	0	0
384	0	1	1	0	0	0	0	0	0	0
385	0	1	2	0	0	0	0	0	0	0
386	0	0	0	0	0	1	0	0	0	0
387	0	0	0	0	0	0	0	0	0	1
388	0	0	0	0	0	0	0	0	0	0

389	0	0	0	0	0	0	0	0	0	0
390	0	1	1	0	0	0	0	0	0	0
391	0	0	0	0	0	0	0	1	0	0
392	0	0	0	1	0	0	0	0	0	0
393	0	0	0	0	0	0	0	1	0	0
394	0	0	0	0	0	0	0	1	0	0
395	0	1	2	0	0	0	0	0	0	0
396	0	1	1	0	0	0	0	0	0	0
397	0	0	0	0	0	0	0	0	0	0
398	1	1	2	0	0	0	0	1	0	0
399	0	0	0	1	0	0	0	0	0	0
400	1	1	3	0	0	0	0	1	0	0
401	0	0	0	1	0	0	0	0	0	0
402	0	1	1	0	0	0	0	0	1	0
403	0	0	0	0	0	0	0	1	0	0
404	0	0	0	1	0	0	0	0	0	0
405	0	0	0	1	0	0	0	0	0	0
406	0	0	0	0	0	0	0	1	0	0
407	0	0	0	0	0	0	0	0	0	0
408	0	0	0	0	0	0	0	1	0	0
409	0	0	0	0	0	0	0	0	0	0
410	0	1	2	0	0	0	0	1	0	0
411	0	0	0	0	0	0	0	1	0	0
412	0	0	0	0	0	0	0	0	0	0
413	0	0	0	0	0	0	0	1	0	0

414	0	0	0	0	0	0	0	0	0	1
415	0	0	0	0	1	0	0	0	0	0
416	0	0	0	0	1	0	0	0	0	0
417	0	0	0	1	0	0	0	0	0	0
418	0	0	0	0	0	0	0	0	0	1
419	0	0	0	0	0	1	0	0	0	0
420	0	1	2	0	0	0	0	0	1	0
421	0	1	1	0	0	1	0	0	0	0
422	0	1	1	0	0	0	0	0	0	0
423	0	0	0	0	0	0	0	0	0	0
424	0	0	0	0	0	0	0	1	0	0
425	0	0	0	0	0	0	0	0	0	0
426	0	0	0	1	0	0	0	0	0	0
427	0	0	0	0	0	0	0	0	0	0
428	0	0	0	0	0	0	0	1	0	0
429	0	0	0	0	1	0	0	0	0	0
430	0	0	0	0	0	1	0	0	0	0
431	0	0	0	0	0	0	0	0	0	0
432	0	0	0	0	0	0	0	0	0	0
433	0	1	1	0	0	0	0	0	0	0
434	0	0	0	0	0	0	0	1	0	0
435	0	0	0	1	0	0	0	0	0	0
436	0	0	0	0	0	0	0	1	0	0
437	0	0	0	0	0	0	0	1	0	0
438	0	0	0	0	1	0	0	0	0	0

439	0	0	0	0	0	0	0	1	0	0
440	0	0	0	0	0	0	0	0	0	0
441	0	0	0	0	0	0	0	1	0	0
442	0	0	0	0	0	0	0	1	0	0
443	0	0	0	0	0	0	0	0	0	1
444	1	1	3	0	0	0	0	1	0	0
445	0	0	0	0	0	0	0	0	0	0
446	0	0	0	0	0	0	0	0	0	0
447	0	0	0	0	0	0	1	0	0	0
448	0	0	0	0	1	0	0	0	0	0
449	0	1	2	0	0	0	0	1	0	0
450	0	0	0	0	0	0	0	0	0	1
451	0	0	0	0	0	0	0	0	0	1
452	1	1	1	0	0	0	0	0	0	1
453	0	0	0	0	0	0	0	0	0	0
454	0	0	0	0	0	0	1	0	0	0
455	0	1	2	0	0	0	0	0	0	0
456	0	1	1	0	0	1	0	0	0	0
457	0	0	0	0	0	0	0	0	1	0
458	0	0	0	0	0	0	1	0	0	0
459	0	1	1	0	0	0	0	1	0	0
460	1	1	3	0	0	0	0	0	1	0
461	0	0	0	0	0	0	0	1	0	0
462	0	0	0	1	0	0	0	0	0	0
463	0	1	2	0	0	0	0	1	0	0

464	0	0	0	0	0	0	0	0	0	0
465	0	0	0	0	1	0	0	0	0	0
466	0	0	0	0	1	0	0	0	0	0
467	0	1	1	0	0	0	0	0	0	0
468	0	0	0	0	0	0	0	0	0	0
469	1	1	3	1	0	0	0	0	0	0
470	0	0	0	0	0	0	0	0	0	0
471	1	1	3	0	0	0	0	1	0	0
472	0	0	0	0	0	0	0	0	0	0
473	0	1	2	0	0	0	0	0	0	0
474	0	0	0	0	0	0	0	1	0	0
475	0	1	1	0	0	0	0	1	0	0
476	0	1	1	0	0	1	0	0	0	0
477	0	1	1	0	0	0	0	0	1	0
478	1	1	1	0	1	0	0	0	0	0
479	0	1	1	1	0	0	0	0	0	0
480	0	1	3	0	1	0	0	0	0	0
481	0	1	1	0	0	0	0	1	0	0
482	0	1	1	0	0	0	0	0	0	1
483	0	1	2	0	0	0	0	0	0	0
484	0	1	2	0	0	0	0	0	0	1
485	0	1	1	0	0	0	0	0	0	0
486	1	1	1	0	0	0	0	0	0	0
487	0	0	0	0	0	0	0	0	0	0
488	1	1	2	0	0	0	0	0	0	1

489	0	1	1	0	1	0	0	0	0	0
490	0	1	3	0	1	0	0	0	0	0
491	0	1	3	0	0	0	0	1	0	0
492	0	1	1	0	0	0	0	1	0	0
493	0	0	0	0	0	0	0	1	0	0
494	0	1	2	0	0	0	0	1	0	0
495	0	1	1	0	0	0	0	1	0	0
496	0	0	0	0	0	0	0	0	0	0
497	0	1	2	0	0	0	0	1	0	0
498	0	0	0	0	0	0	0	0	0	0
499	0	0	0	0	0	0	0	0	0	0
500	0	0	0	0	0	0	0	0	0	1
501	0	1	1	1	0	0	0	0	0	0
502	0	1	1	0	0	0	0	0	0	1
503	0	0	0	0	0	0	0	0	0	0
504	0	0	0	0	0	0	0	0	0	1
505	0	0	0	0	0	0	0	0	0	1
506	0	1	1	0	0	0	0	1	0	0
507	0	1	1	0	0	0	0	0	1	0
508	0	1	1	0	0	0	0	1	0	0
509	0	1	1	0	0	0	0	0	0	0
510	1	1	1	0	0	0	1	0	0	0
511	0	0	0	0	0	0	0	1	0	0
512	0	0	0	1	0	0	0	0	0	0
513	0	0	0	0	0	0	0	0	0	1

514	0	0	0	0	0	0	0	0	0	1
515	0	1	2	0	0	0	0	1	0	0
516	0	0	0	0	0	0	0	1	0	0
517	0	0	0	0	0	0	0	0	0	0
518	0	0	0	0	0	0	0	1	0	0
519	0	0	0	0	0	0	0	0	0	0
520	0	0	0	0	0	0	0	0	0	0
521	0	0	0	0	0	0	0	0	0	1
522	0	1	1	0	0	0	0	1	0	0
523	0	0	0	0	0	0	1	0	0	0
524	0	1	2	0	0	0	0	1	0	0
525	0	0	0	0	0	0	0	0	0	0
526	1	1	3	0	0	0	0	0	0	0
527	0	0	0	0	0	0	0	0	0	0
528	0	0	0	0	0	1	0	0	0	0
529	0	0	0	0	0	1	0	0	0	0
530	0	0	0	0	1	0	0	0	0	0
531	0	1	1	0	0	0	0	0	0	0
532	0	0	0	1	0	0	0	0	0	0
533	0	0	0	0	0	0	0	1	0	0
534	0	1	1	0	0	0	0	1	0	0
535	0	0	0	0	0	0	0	0	0	0
536	0	0	0	0	0	0	0	0	1	0
537	0	0	0	0	0	0	0	0	0	0
538	0	1	3	0	0	0	0	0	1	0

539	1	1	2	1	0	0	0	0	0	0
540	0	0	0	0	0	0	0	0	1	0
541	0	1	2	0	0	0	0	1	0	0
542	0	0	0	0	0	0	0	0	0	0
543	0	0	0	0	0	0	0	1	0	0
544	0	0	0	0	0	0	0	0	0	0
545	0	0	0	0	0	0	0	1	0	0
546	0	0	0	0	1	0	0	0	0	0
547	0	1	3	0	0	0	0	0	0	0
548	0	0	0	1	0	0	0	0	0	0
549	0	1	1	0	0	0	0	1	0	0
550	0	1	1	0	0	0	0	0	1	0
551	0	0	0	0	0	0	0	1	0	0
552	0	1	3	0	1	0	0	0	0	0
553	0	1	2	0	0	0	0	1	0	0
554	0	0	0	0	0	0	0	1	0	0
555	0	1	2	1	0	0	0	0	0	0
556	0	0	0	0	0	0	0	1	0	0
557	1	1	1	0	0	1	0	0	0	0
558	1	1	2	0	0	0	0	0	0	1
559	0	1	2	1	0	0	0	0	0	0
560	0	1	2	0	0	0	0	0	1	0
561	0	1	1	0	0	0	0	0	0	1
562	0	0	0	0	0	0	0	1	0	0
563	1	1	1	0	1	0	0	0	0	0

564	0	0	0	1	0	0	0	0	0	0
565	0	1	1	0	0	1	0	0	0	0
566	0	1	1	0	1	0	0	0	0	0
567	0	1	2	0	0	0	0	0	1	0
568	0	1	1	0	0	0	0	0	1	0
569	0	1	2	0	0	0	0	0	0	0
570	0	0	0	1	0	0	0	0	0	0
571	0	0	0	0	0	0	0	0	0	0
572	1	1	3	0	0	0	0	1	0	0
573	0	1	1	0	1	0	0	0	0	0
574	0	1	1	0	0	0	0	1	0	0
575	0	1	2	0	0	0	0	0	0	0
576	0	1	1	0	0	0	0	0	0	1
577	0	0	0	0	0	0	0	1	0	0
578	0	1	1	0	0	0	1	0	0	0
579	0	1	1	0	0	0	0	1	0	0
580	0	1	2	0	0	0	0	0	0	1
581	0	1	2	0	0	0	0	0	0	0
582	0	1	1	0	0	0	0	1	0	0
583	0	1	1	0	0	0	0	1	0	0
584	0	1	2	0	0	0	0	1	0	0
585	0	1	1	0	0	0	0	1	0	0
586	0	0	0	1	0	0	0	0	0	0
587	0	1	1	0	0	0	0	0	0	0
588	0	0	0	0	1	0	0	0	0	0

589	0	1	1	0	0	0	0	0	0	0
590	0	0	0	0	0	0	0	0	0	0
591	0	0	0	0	0	0	0	0	0	0
592	0	0	0	0	0	0	0	0	0	1
593	1	1	2	0	0	0	0	0	0	1
594	1	1	2	0	1	0	0	0	0	0
595	0	1	2	0	0	0	0	0	0	0
596	0	1	1	0	0	0	0	1	0	0
597	0	1	1	0	0	0	0	0	0	0
598	1	1	1	0	0	0	0	0	0	1
599	0	0	0	0	0	0	0	0	0	0
600	0	0	0	0	0	0	0	0	0	0
601	0	0	0	0	0	0	0	1	0	0
602	0	0	0	0	0	0	0	0	1	0
603	0	0	0	0	0	0	0	1	0	0
604	0	0	0	0	0	0	0	0	0	0
605	0	0	0	0	0	0	0	0	0	0
606	0	1	2	0	1	0	0	0	0	0
607	0	1	2	0	0	0	0	1	0	0
608	0	1	2	0	0	1	0	0	0	0
609	0	0	0	0	0	0	0	0	0	0
610	0	1	1	0	0	0	0	0	0	0
611	0	1	1	0	0	0	0	1	0	0
612	0	1	1	0	0	0	0	1	0	0
613	0	1	1	0	0	0	0	0	0	0

614	0	1	1	0	0	0	0	0	0	0
615	0	0	0	0	0	0	0	0	0	1
616	0	1	1	0	0	0	0	1	0	0
617	0	0	0	0	0	0	0	0	0	0
618	0	1	1	0	0	0	0	1	0	0
619	0	1	1	0	0	0	0	1	0	0
620	0	0	0	0	0	0	0	0	0	0
621	0	0	0	0	0	0	0	1	0	0
622	0	1	1	0	0	0	0	0	0	0
623	0	1	1	0	0	0	0	0	0	1
624	0	1	1	0	0	0	0	0	0	1
625	0	1	1	0	0	0	0	1	0	0
626	0	0	0	0	0	0	0	1	0	0
627	0	0	0	0	0	0	0	1	0	0
628	0	0	0	0	0	0	0	0	1	0
629	0	0	0	1	0	0	0	0	0	0
630	0	0	0	1	0	0	0	0	0	0
631	0	0	0	0	0	0	0	0	0	1
632	0	0	0	0	0	0	0	0	0	0
633	0	0	0	0	0	0	0	1	0	0
634	0	0	0	1	0	0	0	0	0	0
635	1	1	2	0	0	0	0	0	0	0
636	0	1	2	0	0	0	0	0	0	0
637	0	1	2	0	0	0	0	1	0	0
638	0	1	2	0	0	0	0	0	0	1

639	0	1	1	0	0	0	0	0	0	0
640	0	0	0	1	0	0	0	0	0	0
641	0	1	2	0	0	0	0	0	0	0
642	1	1	1	0	0	0	0	1	0	0
643	0	1	1	0	0	0	0	0	1	0
644	0	0	0	0	0	0	0	0	0	0
645	0	1	2	0	0	0	0	1	0	0
646	0	1	1	0	0	1	0	0	0	0
647	0	1	2	0	0	0	0	0	0	0
648	0	1	2	0	0	0	0	0	0	0
649	0	1	3	0	0	0	0	0	0	0
650	0	0	0	0	0	0	0	1	0	0
651	0	0	0	0	0	0	0	1	0	0
652	0	0	0	0	0	0	0	1	0	0
653	0	1	1	0	0	0	0	1	0	0
654	0	0	0	0	0	0	0	0	0	1
655	0	0	0	0	0	0	0	0	0	0
656	0	0	0	0	0	0	0	0	0	0
657	0	0	0	0	0	0	0	0	0	0
658	0	1	2	0	0	0	0	0	0	0
659	0	0	0	0	1	0	0	0	0	0
660	0	1	1	0	0	0	0	0	0	0
661	0	1	2	1	0	0	0	0	0	0
662	0	0	0	0	0	0	0	1	0	0
663	0	0	0	0	0	0	0	0	0	1

664	0	1	1	0	0	0	0	0	0	0
665	0	0	0	0	0	0	1	0	0	0
666	0	0	0	0	0	0	0	0	0	0
667	0	1	2	0	0	0	0	0	0	0
668	0	1	3	0	0	0	0	1	0	0
669	0	0	0	0	0	0	0	1	0	0
670	1	1	2	0	0	0	0	0	0	1
671	0	0	0	1	0	0	0	0	0	0
672	0	0	0	0	0	0	0	0	0	0
673	0	0	0	1	0	0	0	0	0	0
674	0	0	0	0	0	0	0	0	0	0
675	1	1	1	0	0	0	0	0	0	1
676	0	0	0	0	0	0	0	0	0	0
677	1	1	2	0	0	0	0	0	0	1
678	0	0	0	0	0	0	0	1	0	0
679	0	1	1	0	0	0	0	0	0	1
680	0	0	0	0	0	0	0	0	0	0
681	0	0	0	0	0	0	0	1	0	0
682	1	1	1	0	0	0	0	0	0	1
683	0	0	0	0	0	0	0	1	0	0
684	0	1	1	0	0	0	0	1	0	0
685	0	0	0	0	0	0	0	1	0	0
686	0	0	0	0	0	0	0	1	0	0
687	0	0	0	0	0	0	0	0	0	0
688	0	1	2	0	0	0	0	0	1	0

689	0	0	0	0	0	0	0	1	0	0
690	0	0	0	0	0	0	0	0	0	0
691	1	1	3	0	0	0	0	0	0	1
692	0	0	0	0	0	0	0	0	0	0
693	0	0	0	0	0	0	0	0	0	0
694	0	0	0	0	1	0	0	0	0	0
695	0	1	3	0	0	0	0	1	0	0
696	0	0	0	0	0	0	0	0	0	0
697	0	0	0	0	0	0	0	1	0	0
698	0	1	2	0	0	0	0	1	0	0
699	1	1	3	0	0	0	0	0	0	0
700	0	1	3	1	0	0	0	0	0	0
701	0	0	0	0	0	0	0	1	0	0
702	0	0	0	0	0	0	0	0	0	0
703	1	1	2	0	0	0	0	0	0	1
704	0	0	0	0	0	0	0	0	0	0
705	0	0	0	1	0	0	0	0	0	0
706	0	0	0	1	0	0	0	0	0	0
707	0	0	0	0	0	1	0	0	0	0
708	0	0	0	0	0	0	0	1	0	0
709	0	0	0	0	0	1	0	0	0	0
710	0	0	0	0	0	0	0	1	0	0
711	0	0	0	0	0	0	0	1	0	0
712	0	1	3	0	0	0	0	1	0	0
713	1	1	2	0	0	0	0	1	0	0

714	0	0	0	0	0	0	0	0	0	0
715	0	0	0	0	0	0	0	0	0	0
716	0	1	1	0	0	0	1	0	0	0
717	0	0	0	0	0	0	0	1	0	0
718	0	1	2	0	0	0	0	1	0	0
719	0	0	0	0	0	0	0	0	0	0
720	1	1	1	0	0	0	0	1	0	0
721	0	0	0	0	0	0	0	0	0	0
722	1	1	3	0	0	1	0	0	0	0
723	0	0	0	0	0	0	1	0	0	0
724	0	0	0	0	0	0	0	1	0	0
725	0	0	0	0	0	0	0	0	0	1
726	0	1	2	0	0	0	1	0	0	0
727	0	0	0	0	0	1	0	0	0	0
728	0	0	0	0	0	0	0	1	0	0
729	0	0	0	0	0	0	0	0	0	0
730	0	1	2	0	0	0	0	0	0	0
731	0	1	3	0	0	0	0	1	0	0
732	1	1	3	0	0	0	0	1	0	0
733	0	0	0	0	0	0	0	0	0	0
734	0	1	1	0	0	0	0	1	0	0
735	0	1	1	0	0	0	1	0	0	0
736	0	0	0	0	0	0	0	1	0	0
737	0	0	0	0	0	0	0	0	0	0
738	0	0	0	0	0	0	1	0	0	0

739	0	0	0	1	0	0	0	0	0	0
740	0	1	2	0	1	0	0	0	0	0
741	0	1	3	0	0	0	0	0	0	1
742	0	1	2	0	0	0	0	1	0	0
743	0	1	1	1	0	0	0	0	0	0
744	0	0	0	0	0	1	0	0	0	0
745	0	0	0	0	0	0	0	0	1	0
746	1	1	3	0	0	0	0	0	0	1
747	0	0	0	0	0	0	0	1	0	0
748	0	0	0	0	0	0	1	0	0	0
749	0	0	0	1	0	0	0	0	0	0
750	0	1	1	0	0	0	0	0	0	0
751	0	0	0	0	0	0	0	0	0	0
752	0	0	0	0	1	0	0	0	0	0
753	0	1	2	0	0	0	0	0	0	1
754	0	1	1	0	0	0	0	1	0	0
755	0	1	3	0	0	0	0	0	0	0
756	1	1	2	1	0	0	0	0	0	0
757	0	0	0	0	0	0	0	0	0	0
758	0	1	1	0	0	0	0	0	1	0
759	0	1	1	0	0	0	0	0	1	0
760	1	1	3	1	0	0	0	0	0	0
761	0	0	0	0	0	1	0	0	0	0
762	1	1	3	0	0	0	1	0	0	0
763	0	1	2	0	0	0	0	0	0	0

764	1	1	2	0	0	1	0	0	0	0
765	0	1	1	1	0	0	0	0	0	0
766	0	1	1	0	0	0	0	0	0	0
767	0	0	0	0	0	0	0	0	0	0
768	0	1	1	0	0	0	0	0	0	0
769	0	1	2	0	0	0	0	1	0	0
770	1	1	3	0	0	0	0	0	0	0
771	1	1	3	0	0	1	0	0	0	0
772	0	0	0	1	0	0	0	0	0	0
773	0	0	0	0	0	0	0	0	1	0
774	0	0	0	0	0	0	0	0	0	0
775	0	1	2	0	0	1	0	0	0	0
776	0	0	0	0	0	0	0	0	1	0
777	1	1	3	0	0	0	0	0	0	0
778	0	0	0	1	0	0	0	0	0	0
779	0	1	1	0	0	0	0	1	0	0
780	0	1	3	0	0	0	0	0	0	0
781	0	1	1	0	0	0	0	1	0	0
782	0	1	2	0	0	0	0	1	0	0
783	1	1	2	1	0	0	0	0	0	0
784	0	0	0	0	0	0	0	0	0	0
785	0	0	0	1	0	0	0	0	0	0
786	0	1	1	0	0	0	1	0	0	0
787	0	1	1	0	0	0	0	1	0	0
788	0	1	2	0	0	0	0	0	1	0

789	0	1	1	0	0	0	0	1	0	0
790	0	1	2	0	0	0	0	0	0	1
791	0	0	0	0	0	0	0	0	0	0
792	0	1	1	0	0	0	0	0	0	0
793	0	0	0	0	0	0	0	0	0	0
794	0	1	2	0	0	0	0	1	0	0
795	0	1	1	0	0	0	0	1	0	0
796	0	0	0	1	0	0	0	0	0	0
797	0	0	0	1	0	0	0	0	0	0
798	0	0	0	1	0	0	0	0	0	0

number	cervical cancer	PS(0-2 good; 3-5 poor)	Average background pain score	Pain score at discharge	Distress score	Barthel index	pain	dyspnea	cough	nausea and vomiting
1	0	1	4	2	3	85	1	0	1	1
2	0	2	4	2	2	0	1	0	1	1
3	0	3	3	2	2	5	1	1	0	0
4	0	3	4	2	2	10	1	1	0	1
5	0	3	6	1	2	15	1	0	0	1
6	0	1	1	2	1	20	1	1	1	0
7	0	1	3	2	3	25	1	1	1	0
8	0	3	5	2	2	30	1	1	0	1
9	0	4	4	1	2	35	1	1	1	0
10	0	2	6	1	2	40	1	0	1	1

11	0	3	3	2	3	45	1	1	0	0
12	0	3	3	2	3	55	1	0	0	1
13	0	3	3	2	2	50	1	1	1	0
14	0	1	6	2	3	55	1	1	1	0
15	0	2	3	3	3	60	1	0	0	0
16	0	3	7	2	3	65	1	0	1	1
17	0	2	5	1	2	70	1	1	1	1
18	0	3	5	2	2	70	1	0	0	1
19	0	0	4	2	2	60	1	1	0	1
20	0	1	4	2	2	50	1	1	0	0
21	0	2	5	2	2	70	1	1	1	0
22	0	3	8	2	3	75	1	0	1	1
23	0	4	4	2	3	70	1	0	0	0
24	0	0	5	2	2	80	1	1	0	0
25	0	1	6	2	3	50	1	1	1	0
26	0	2	4	2	1	70	1	1	1	0
27	0	3	5	2	2	70	1	0	0	0
28	0	4	4	2	2	70	1	1	0	1
29	0	0	4	2	1	70	1	0	1	1
30	0	1	6	2	2	50	1	1	0	0
31	1	2	3	2	2	60	1	0	0	0
32	0	3	3	2	2	75	1	0	0	1
33	0	4	2	2	2	80	1	1	0	0
34	0	0	0	2	2	90	1	1	0	1
35	0	1	2	2	1	60	1	1	0	0

36	0	2	2	2	1	50	1	1	1	0
37	0	3	2	2	2	70	1	1	1	0
38	0	4	3	2	2	100	1	1	1	1
39	0	0	2	2	2	85	1	0	0	0
40	0	1	2	2	2	90	1	0	1	1
41	1	2	5	5	2	20	1	1	0	0
42	0	3	2	2	2	50	1	1	1	0
43	0	4	5	2	2	5	1	0	0	0
44	0	0	6	2	3	70	1	1	0	1
45	0	1	4	2	3	40	1	0	0	0
46	0	2	4	2	1	50	1	0	1	1
47	0	3	5	1	3	50	1	1	0	1
48	0	4	3	3	2	50	1	1	1	0
49	0	0	4	1	2	50	1	1	0	0
50	0	1	6	2	2	30	1	0	1	1
51	0	2	3	2	2	50	1	0	1	1
52	0	3	3	2	2	10	1	1	0	0
53	0	4	4	2	3	95	1	1	1	0
54	0	0	7	2	2	50	1	0	0	0
55	0	1	3	2	2	50	1	1	1	1
56	0	2	2	2	2	100	1	1	1	1
57	0	3	1	0	2	100	1	0	0	0
58	0	4	5	2	2	40	1	1	0	1
59	0	0	2	2	3	0	1	0	0	0
60	0	1	3	2	2	50	1	0	1	0

61	1	2	2	2	3	50	1	0	0	0
62	0	3	6	2	3	50	1	1	0	0
63	0	4	6	2	1	5	1	0	0	0
64	0	4	3	2	2	10	1	1	0	0
65	0	2	7	2	1	75	1	1	0	0
66	0	0	5	2	1	90	1	0	1	1
67	0	2	4	3	3	65	1	0	1	0
68	1	1	5	2	1	35	1	0	0	0
69	0	3	5	2	5	50	1	0	1	1
70	0	4	5	2	1	20	1	1	1	0
71	0	1	6	2	2	65	1	0	1	0
72	1	2	4	1	1	45	0	0	0	0
73	0	3	3	3	2	25	1	1	0	0
74	0	1	4	2	1	60	1	0	0	1
75	0	3	5	1	3	45	1	0	0	1
76	0	3	4	3	3	35	1	0	0	1
77	0	3	4	2	1	45	1	0	0	1
78	1	4	7	3	1	55	1	1	1	1
79	0	3	3	3	1	70	1	1	1	1
80	0	3	4	2	2	50	1	1	1	1
81	0	1	5	2	2	100	1	0	0	1
82	0	3	3	2	2	50	1	1	0	1
83	0	3	3	2	3	80	1	0	0	1
84	0	2	4	2	1	85	1	0	0	1
85	0	3	4	2	2	35	1	1	1	1

86	0	2	5	2	2	65	1	1	1	0
87	0	3	7	2	2	100	1	0	1	1
88	0	3	5	1	2	75	1	0	0	0
89	0	2	4	1	3	80	1	0	0	1
90	0	4	5	2	1	40	1	0	0	1
91	0	3	4	2	2	100	1	1	0	1
92	0	1	5	3	1	95	1	0	0	0
93	0	4	4	1	2	60	1	1	1	0
94	0	3	3	1	1	80	1	0	0	0
95	0	2	3	1	2	70	1	0	0	0
96	0	1	2	2	3	80	0	1	1	0
97	0	3	5	1	2	90	1	0	0	1
98	0	2	2	1	2	60	1	1	1	0
99	0	3	4	3	4	90	1	0	1	0
100	0	3	2	2	2	65	1	1	0	0
101	0	2	3	3	5	10	1	0	0	1
102	0	2	5	1	1	100	1	0	0	0
103	0	4	2	2	3	30	1	1	0	1
104	0	4	6	2	3	45	1	1	1	1
105	0	3	6	2	2	70	1	0	0	0
106	0	3	4	2	2	90	1	0	1	1
107	0	2	4	4	3	90	1	0	0	0
108	0	4	1	1	3	25	0	1	1	1
109	0	4	0	0	2	75	0	1	1	1
110	0	3	3	2	1	40	1	0	0	0

111	0	3	3	2	1	70	1	0	0	0
112	0	2	4	2	3	60	1	0	0	0
113	0	1	5	2	3	10	1	0	1	1
114	0	3	4	2	1	100	1	0	0	1
115	0	1	3	2	3	75	1	0	1	0
116	0	4	6	2	2	5	1	0	1	1
117	0	2	4	2	2	35	1	0	0	0
118	0	3	5	2	3	50	1	0	1	0
119	0	2	5	2	2	75	1	0	1	0
120	0	2	4	2	3	50	1	0	0	1
121	0	2	4	2	1	45	1	0	0	0
122	0	2	7	2	1	55	1	0	1	1
123	0	1	5	2	1	10	1	0	0	0
124	0	1	4	2	2	40	1	1	0	1
125	1	2	4	2	1	85	1	0	0	0
126	0	3	3	2	1	100	0	0	0	1
127	0	4	6	2	3	60	1	1	1	0
128	0	0	6	2	2	75	1	0	0	1
129	1	1	5	2	1	50	1	0	0	0
130	0	2	5	3	3	43	1	0	0	0
131	0	3	4	2	1	15	1	0	0	0
132	0	4	4	1	3	50	1	1	1	0
133	0	0	6	2	3	30	1	0	0	0
134	1	1	4	2	1	100	1	0	1	1
135	0	2	6	1	2	85	1	0	0	0

136	0	3	4	2	1	65	1	1	1	0
137	0	4	6	2	1	40	1	0	1	0
138	0	0	3	2	2	55	1	0	1	0
139	0	1	4	2	3	15	1	0	1	1
140	0	3	5	2	3	30	1	0	0	0
141	0	3	4	2	4	75	0	1	0	0
142	1	1	5	2	2	80	1	0	0	0
143	0	4	7	2	2	20	1	0	0	1
144	0	3	5	2	3	50	1	0	0	0
145	0	2	5	1	2	70	1	0	0	1
146	0	1	4	3	4	80	0	0	0	0
147	0	1	7	2	4	80	1	1	1	1
148	0	1	3	2	2	100	1	0	0	0
149	0	1	6	2	3	50	1	1	0	1
150	1	2	4	2	2	90	1	0	0	0
151	0	1	4	3	2	70	1	0	1	1
152	0	2	7	2	2	50	1	0	0	1
153	0	2	5	1	2	70	0	0	1	1
154	0	2	6	2	2	75	0	1	1	0
155	0	1	2	2	3	90	1	0	0	1
156	0	1	8	2	1	100	1	1	0	0
157	0	3	4	2	2	70	1	1	1	0
158	1	2	4	3	2	60	1	0	0	0
159	0	2	6	2	2	50	1	0	0	0
160	0	2	6	2	2	70	1	0	1	1

161	0	1	7	1	2	70	1	0	0	0
162	0	2	4	2	3	50	1	0	1	0
163	0	2	4	2	2	100	1	0	0	0
164	0	1	0	2	1	50	1	0	1	1
165	0	2	5	2	3	100	1	0	0	0
166	0	1	8	2	2	70	1	0	0	1
167	0	1	8	2	2	70	1	0	0	0
168	0	2	4	2	3	50	1	0	0	0
169	0	3	4	2	3	70	1	0	0	0
170	0	2	4	3	3	50	1	0	0	0
171	0	3	8	2	1	70	1	0	0	0
172	0	3	4	2	1	70	1	0	0	1
173	0	3	5	2	2	70	1	0	1	1
174	0	1	4	2	2	50	1	0	0	1
175	0	1	6	2	3	70	1	0	0	0
176	0	2	4	2	1	70	1	1	0	0
177	0	0	3	2	2	50	1	0	0	0
178	0	2	7	2	2	70	1	1	1	1
179	0	2	3	2	1	50	1	0	0	0
180	0	0	3	1	1	70	1	0	0	0
181	0	0	2	1	2	90	1	0	0	0
182	0	1	4	2	2	50	1	0	0	0
183	0	3	4	2	2	50	1	0	0	1
184	1	2	7	2	2	70	1	0	0	0
185	0	1	5	2	1	80	1	0	0	0

186	0	4	6	3	2	70	1	0	0	0
187	1	1	4	2	1	20	1	0	1	0
188	0	3	4	1	3	50	1	0	0	0
189	0	1	4	2	2	70	1	0	0	0
190	0	4	4	2	3	30	1	0	0	1
191	0	3	4	2	3	40	1	1	1	0
192	0	1	4	1	3	40	1	0	0	0
193	0	1	4	2	3	50	1	0	0	0
194	1	1	4	1	3	70	1	0	0	1
195	0	2	4	2	3	30	1	0	0	0
196	0	2	4	1	3	60	1	0	0	0
197	0	1	4	2	1	90	1	0	0	0
198	0	1	1	2	1	70	1	0	0	0
199	0	1	5	2	1	100	1	1	0	1
200	0	1	4	2	3	90	1	0	0	0
201	0	3	4	1	2	40	1	0	0	0
202	0	2	5	2	2	20	1	1	1	0
203	0	2	4	1	2	60	1	0	0	0
204	0	3	5	1	2	90	1	0	0	0
205	0	0	6	0	1	100	1	0	0	1
206	0	1	2	2	2	70	1	0	1	0
207	0	3	4	2	3	25	1	0	0	0
208	0	1	4	2	2	60	1	1	0	0
209	0	3	4	1	2	75	1	0	0	0
210	0	4	5	2	2	40	1	0	0	0

211	0	3	4	1	6	40	1	0	0	0
212	0	2	4	2	2	75	1	0	0	0
213	0	1	3	2	2	100	1	1	0	0
214	0	0	4	2	2	80	1	0	0	0
215	0	1	4	2	2	70	1	0	0	1
216	0	2	5	2	2	75	1	0	1	0
217	0	2	6	2	3	50	1	0	0	0
218	0	2	7	2	2	70	1	0	0	0
219	0	3	5	3	2	70	1	0	1	0
220	0	2	6	2	2	75	1	1	0	0
221	0	2	4	2	2	90	1	0	0	1
222	0	2	4	1	1	25	1	0	0	0
223	0	1	5	2	2	70	1	0	0	1
224	0	1	4	2	2	90	1	0	0	1
225	0	0	2	2	1	95	1	0	0	0
226	0	2	4	2	3	70	1	1	0	0
227	0	1	5	1	2	50	1	0	0	1
228	0	0	4	2	2	70	1	0	0	0
229	0	1	4	2	3	70	1	0	0	1
230	0	2	4	1	2	50	1	0	0	1
231	1	2	5	2	1	30	1	0	0	0
232	0	1	4	3	2	70	1	0	0	0
233	0	2	4	1	2	50	1	0	0	0
234	1	2	6	2	3	50	1	0	1	1
235	1	1	2	1	2	70	0	0	0	0

236	0	3	6	2	2	35	1	0	0	1
237	0	1	4	2	2	40	1	1	0	0
238	0	2	4	2	3	45	1	0	0	1
239	0	3	4	2	2	50	0	0	0	0
240	0	1	6	2	2	50	1	0	0	0
241	0	1	4	1	2	50	1	1	0	1
242	0	2	3	2	2	55	1	0	0	1
243	0	1	4	2	2	30	1	0	0	0
244	0	1	6	2	2	35	1	1	0	1
245	0	1	4	2	2	35	1	0	0	0
246	0	2	6	2	3	50	1	0	1	1
247	0	1	5	2	2	40	1	0	0	0
248	0	2	4	1	3	50	1	1	0	0
249	0	1	5	2	3	80	1	0	0	1
250	0	1	5	2	2	60	1	0	0	0
251	0	2	5	2	1	65	1	0	1	0
252	0	1	4	2	2	70	1	0	0	1
253	0	1	1	2	2	100	1	0	0	1
254	1	0	0	2	2	100	1	0	0	1
255	0	2	7	2	1	100	1	0	0	1
256	1	3	5	1	2	70	1	0	0	0
257	0	2	5	4	2	50	1	0	0	0
258	0	1	4	2	2	75	1	0	0	0
259	0	1	5	2	2	70	1	0	0	0
260	0	1	4	2	2	85	1	0	0	1

261	0	4	5	2	2	50	1	0	0	0
262	0	2	6	2	2	60	1	1	0	0
263	0	3	6	2	2	50	1	0	0	0
264	0	1	5	2	2	50	1	0	0	1
265	0	3	3	2	2	75	0	0	0	0
266	0	3	4	2	2	70	0	1	1	1
267	0	0	3	1	2	100	1	0	0	0
268	0	1	0	1	3	50	0	0	1	0
269	0	2	5	1	1	70	1	0	0	0
270	0	1	4	1	2	70	1	0	0	0
271	0	1	6	1	2	50	1	0	0	0
272	0	2	6	2	1	70	1	0	0	1
273	0	1	5	2	2	70	1	0	0	0
274	0	1	5	3	1	70	1	0	0	0
275	0	1	5	2	1	85	1	1	1	0
276	0	3	7	2	1	70	1	0	0	0
277	0	2	4	2	1	100	1	0	0	0
278	0	2	4	2	2	50	1	0	0	0
279	0	3	6	2	2	45	1	0	0	1
280	0	3	4	2	1	70	1	0	0	0
281	0	4	4	2	1	50	0	1	1	0
282	0	2	8	2	2	50	1	0	0	0
283	0	2	4	2	2	70	1	0	0	0
284	0	1	4	2	2	70	1	0	0	0
285	0	2	6	3	2	50	1	0	0	0

286	0	1	2	2	2	20	1	0	0	0
287	0	2	4	2	1	50	1	0	0	0
288	0	3	4	2	2	80	1	0	0	0
289	0	2	7	2	1	70	1	0	0	0
290	0	3	5	1	2	70	1	0	0	0
291	0	0	0	1	2	80	1	0	0	1
292	0	1	6	2	3	50	1	0	0	0
293	0	1	6	1	2	80	1	0	0	0
294	0	1	4	2	3	70	1	0	0	0
295	0	1	6	1	2	60	1	0	1	1
296	0	1	4	2	2	30	1	0	0	0
297	0	3	6	2	3	70	1	0	0	0
298	0	1	9	2	2	70	1	0	0	0
299	0	2	5	2	2	70	1	0	0	0
300	0	1	4	2	2	80	1	1	1	0
301	0	2	6	2	3	50	1	0	0	0
302	0	2	6	2	1	70	1	0	0	0
303	0	0	2	2	2	70	1	0	0	1
304	0	0	4	2	2	50	1	0	0	0
305	0	2	5	2	3	10	1	0	0	0
306	0	2	8	2	3	70	1	0	0	0
307	0	2	4	2	3	40	1	0	0	1
308	0	1	2	2	3	70	1	1	1	0
309	0	3	5	2	3	50	1	1	0	0
310	0	1	5	2	2	50	1	0	0	0

311	0	1	3	2	1	95	1	0	1	0
312	0	2	2	2	2	70	1	0	0	1
313	0	4	4	2	3	50	1	0	0	0
314	0	3	3	2	2	50	1	0	0	0
315	0	3	3	2	2	70	1	0	0	0
316	0	2	3	2	2	70	1	0	1	0
317	0	3	5	3	2	20	1	0	1	0
318	0	3	3	2	2	70	1	0	0	0
319	0	2	4	2	2	70	1	0	0	0
320	0	1	4	2	2	55	1	0	0	0
321	1	3	2	2	2	60	1	0	0	0
322	0	2	3	2	2	50	1	0	0	1
323	0	1	3	2	2	50	1	0	0	0
324	0	3	5	2	2	65	1	0	0	0
325	0	2	6	2	2	70	1	1	1	0
326	0	3	4	2	3	60	1	0	1	0
327	0	1	4	2	2	70	1	0	0	0
328	0	1	5	2	2	75	1	0	0	0
329	0	1	5	2	1	75	1	0	0	0
330	0	2	5	2	2	80	1	0	0	0
331	0	2	5	2	2	60	1	0	0	0
332	0	2	3	2	2	50	1	0	0	1
333	0	1	4	2	2	85	1	0	0	0
334	0	2	3	2	1	60	1	0	0	0
335	0	2	4	2	2	50	1	0	0	0

336	0	1	5	2	2	70	1	0	1	0
337	0	3	3	2	1	50	1	0	0	1
338	0	3	3	2	2	70	1	0	0	0
339	0	3	3	2	1	60	1	0	1	0
340	0	3	6	2	2	90	1	0	0	0
341	0	4	7	2	1	95	1	0	0	0
342	0	0	2	2	1	70	1	1	1	0
343	0	1	2	2	2	20	1	0	0	0
344	0	0	2	2	1	100	1	1	1	0
345	0	0	2	2	2	5	1	0	0	1
346	0	0	5	2	1	70	1	0	0	0
347	0	2	3	2	2	50	1	0	0	0
348	0	1	2	2	1	10	1	0	1	1
349	0	2	2	2	1	70	1	0	0	0
350	0	1	2	2	2	60	1	0	0	1
351	0	0	5	2	1	70	1	0	1	1
352	0	3	4	2	3	50	1	0	0	0
353	0	0	2	2	1	70	1	0	0	1
354	0	4	3	2	2	15	1	1	1	0
355	0	2	3	2	2	50	1	0	0	0
356	0	2	4	2	1	70	1	0	0	1
357	1	2	6	2	2	70	1	0	0	0
358	0	1	2	2	2	50	1	0	0	1
359	0	2	6	2	2	40	1	0	0	0
360	0	4	3	2	2	60	1	0	0	0

361	0	3	3	2	2	60	1	1	0	0
362	0	1	2	2	2	80	1	0	0	0
363	0	2	2	2	2	70	1	0	0	0
364	0	1	3	3	2	20	1	1	0	0
365	1	1	7	2	3	50	1	0	0	1
366	0	3	3	2	2	25	1	0	0	0
367	1	1	3	2	3	60	1	0	0	0
368	0	3	1	2	2	30	1	1	0	0
369	0	1	4	2	2	85	1	0	0	1
370	0	1	3	2	3	35	1	0	0	0
371	0	1	3	2	2	70	1	0	0	0
372	1	1	2	2	1	70	1	0	0	0
373	0	0	2	2	2	90	1	0	0	0
374	0	4	2	2	2	40	1	1	1	0
375	0	3	3	2	2	50	1	0	0	1
376	0	1	1	2	1	70	1	0	0	0
377	1	2	2	2	2	60	1	0	0	0
378	0	1	3	2	2	100	1	0	0	0
379	0	0	3	2	3	45	1	0	0	1
380	0	3	4	2	2	60	1	0	0	0
381	1	2	3	2	2	50	1	0	0	0
382	0	2	4	2	2	100	1	0	0	1
383	0	4	4	2	1	50	1	1	1	0
384	0	3	4	2	2	50	1	0	0	0
385	0	1	6	2	2	55	1	0	0	1

386	0	1	4	2	2	70	1	1	0	0
387	0	2	3	2	2	80	1	0	0	0
388	0	1	6	2	2	60	1	0	0	0
389	0	2	6	2	2	50	1	0	0	1
390	0	2	3	2	3	65	1	0	0	0
391	0	2	5	2	2	50	1	0	1	0
392	0	3	3	2	2	40	1	1	1	1
393	0	4	3	2	2	50	1	1	1	0
394	0	1	3	2	2	90	1	0	0	0
395	0	1	4	2	2	80	1	0	0	0
396	0	2	3	2	2	80	1	0	0	0
397	0	2	3	2	3	70	1	0	1	0
398	0	3	4	3	2	70	1	0	0	0
399	0	3	4	2	2	50	1	1	1	0
400	0	4	3	2	2	50	1	0	1	0
401	0	1	3	2	3	50	1	0	0	0
402	0	2	5	3	2	35	1	0	0	0
403	0	3	5	2	2	80	1	1	1	0
404	0	2	5	2	2	65	1	0	0	0
405	0	2	2	2	2	70	1	0	0	1
406	0	1	2	2	2	80	1	0	1	0
407	0	1	4	2	2	90	1	0	0	0
408	0	1	3	2	2	75	1	0	0	1
409	0	2	5	2	3	80	1	0	0	0
410	0	2	6	2	2	70	1	1	1	0

411	0	2	4	2	3	30	1	0	0	0
412	1	2	3	2	2	50	1	0	0	1
413	0	2	3	2	2	85	1	0	0	1
414	0	2	3	2	2	90	1	0	0	1
415	0	1	2	2	2	90	1	0	0	0
416	0	3	3	2	2	50	1	0	0	0
417	0	2	3	2	2	95	1	0	0	1
418	0	2	4	2	2	90	1	0	0	0
419	0	1	1	2	2	90	1	0	0	1
420	0	0	4	2	2	100	1	0	0	0
421	0	3	2	2	2	100	1	0	0	0
422	0	3	3	2	2	75	1	0	0	1
423	0	1	6	2	1	60	1	0	0	1
424	0	3	2	2	2	50	1	0	0	0
425	0	2	3	2	2	50	1	0	0	0
426	0	2	2	2	1	5	1	0	0	0
427	0	2	3	2	2	50	1	0	1	0
428	0	2	2	2	2	10	1	1	0	1
429	0	2	2	2	1	15	1	1	0	0
430	0	1	2	2	1	100	1	0	0	0
431	0	0	3	2	2	50	1	1	0	0
432	1	0	3	2	3	50	1	0	0	0
433	0	2	5	2	1	20	1	0	0	0
434	0	3	2	2	1	70	1	0	1	0
435	0	2	6	2	2	90	1	0	0	0

436	0	1	3	2	2	60	1	0	0	0
437	0	1	2	2	2	25	1	0	0	1
438	0	2	6	2	2	30	1	0	0	1
439	0	1	2	2	1	70	0	0	1	0
440	1	2	2	2	2	80	1	0	0	0
441	0	2	2	2	2	35	1	0	1	0
442	0	4	2	2	1	90	1	0	1	0
443	0	1	3	2	2	55	1	0	1	1
444	0	1	6	2	2	100	1	0	0	0
445	0	0	2	2	2	40	1	0	0	0
446	0	2	3	2	2	50	1	0	0	0
447	0	1	2	2	1	45	1	0	0	0
448	0	1	3	2	2	70	1	0	0	0
449	0	1	4	2	3	95	1	0	0	0
450	0	3	3	2	2	75	1	0	0	1
451	0	1	1	2	2	50	1	0	0	0
452	0	3	3	2	2	70	1	0	0	1
453	0	3	6	2	2	50	1	0	0	1
454	0	1	5	2	1	55	1	0	0	1
455	0	2	4	2	2	100	1	0	0	0
456	0	0	3	1	2	100	1	0	0	1
457	0	1	0	2	2	60	1	0	0	0
458	0	2	4	2	2	65	1	0	0	0
459	0	2	6	2	2	50	1	0	0	0
460	0	3	3	2	2	50	1	0	0	0

461	0	4	4	2	3	50	1	0	0	0
462	0	2	4	2	3	70	1	0	0	0
463	0	0	3	1	1	80	1	0	0	1
464	0	0	0	1	1	70	0	0	0	1
465	0	2	6	2	3	50	1	0	0	0
466	0	1	4	3	1	50	0	0	0	0
467	0	2	5	3	3	85	0	0	0	0
468	0	1	1	2	2	70	1	0	0	0
469	0	2	3	2	2	50	1	0	0	0
470	1	2	3	2	2	50	1	0	0	0
471	0	1	3	2	1	90	1	0	0	0
472	0	3	2	1	1	70	1	1	0	0
473	0	3	5	2	2	70	1	1	0	0
474	0	0	3	2	2	50	1	0	1	0
475	0	3	2	2	2	50	1	0	0	0
476	0	3	2	2	2	70	1	0	0	0
477	0	1	2	2	2	50	0	0	0	0
478	0	1	3	1	2	50	1	0	0	0
479	0	1	4	2	1	95	1	0	0	0
480	0	1	3	2	2	50	1	0	0	0
481	0	0	3	1	3	70	1	0	1	0
482	0	2	6	3	3	50	1	0	0	0
483	0	4	6	2	3	100	1	1	0	1
484	0	3	6	2	3	70	1	1	0	0
485	0	3	1	2	1	5	1	0	0	0

486	0	3	4	2	2	65	1	0	0	0
487	0	2	3	2	1	70	1	0	0	0
488	0	1	3	2	3	50	1	0	0	0
489	0	3	4	2	3	70	1	0	0	1
490	0	1	3	3	3	10	0	0	0	0
491	0	2	6	2	2	15	0	0	1	0
492	0	2	7	2	2	50	1	0	0	0
493	0	1	3	2	2	50	1	0	0	0
494	0	1	6	2	3	50	1	0	0	0
495	0	2	3	2	2	50	1	0	1	0
496	0	3	3	2	2	70	1	0	0	0
497	0	2	3	2	3	50	1	0	0	0
498	0	1	6	2	1	50	1	0	0	0
499	1	2	3	2	2	75	1	0	0	0
500	0	3	3	2	2	80	1	0	0	0
501	0	0	2	2	2	80	1	1	0	0
502	0	1	8	5	1	100	1	0	0	0
503	0	0	3	2	2	80	1	0	0	0
504	0	3	4	3	3	85	1	0	0	0
505	0	3	3	6	2	80	1	0	0	0
506	0	3	3	2	3	20	1	0	1	1
507	0	1	6	2	2	25	1	0	0	1
508	0	3	3	2	2	30	1	0	0	1
509	0	4	4	2	2	35	1	0	0	0
510	0	2	4	2	2	100	1	0	0	0

511	0	3	3	2	3	55	0	0	0	0
512	0	2	3	2	2	50	0	0	0	0
513	0	2	2	2	2	90	1	0	0	0
514	0	2	2	2	2	95	1	0	0	0
515	0	2	6	2	3	50	0	0	0	1
516	0	1	6	3	2	100	0	0	0	0
517	0	2	3	2	3	50	0	0	0	0
518	0	3	2	2	1	50	1	0	0	0
519	1	2	2	2	1	65	1	0	0	0
520	1	2	3	2	2	50	1	0	0	1
521	0	2	3	2	2	50	1	0	0	0
522	0	2	6	2	3	70	1	1	1	0
523	0	2	3	2	2	5	1	0	0	0
524	0	2	3	2	3	65	1	0	1	0
525	0	2	6	2	2	90	1	0	0	0
526	0	3	3	2	2	70	1	0	0	0
527	0	4	5	2	2	10	1	0	1	0
528	0	2	3	2	2	60	1	0	0	0
529	0	2	4	2	2	50	1	0	0	0
530	0	0	2	3	2	60	1	0	0	0
531	0	1	3	2	2	100	1	0	0	1
532	0	1	3	2	2	40	1	0	0	1
533	0	2	3	2	2	60	0	0	0	0
534	0	2	3	2	2	50	1	0	0	0
535	0	0	4	2	2	50	1	0	1	0

536	0	0	0	2	2	65	1	0	0	0
537	1	2	3	2	3	50	1	0	0	0
538	0	1	1	2	2	50	1	0	0	0
539	0	0	2	2	2	70	1	0	0	0
540	0	3	3	2	3	40	1	0	0	0
541	0	0	5	2	2	100	1	1	1	0
542	0	1	4	2	3	50	1	0	0	0
543	0	1	5	1	3	70	1	1	0	0
544	0	3	0	0	2	40	0	1	1	0
545	0	2	3	2	2	15	1	0	0	1
546	0	2	2	2	3	50	1	1	0	0
547	0	1	3	2	3	50	1	0	0	1
548	0	0	3	2	3	50	1	0	0	0
549	0	3	3	1	1	85	1	1	0	0
550	0	1	3	2	3	50	1	0	0	0
551	0	1	2	2	3	60	1	0	0	0
552	0	1	5	3	3	60	1	0	0	1
553	0	1	2	2	3	50	0	1	0	0
554	0	2	7	2	3	20	1	0	0	1
555	0	1	8	2	2	70	1	0	0	1
556	0	3	3	0	2	50	1	0	0	0
557	0	1	5	2	3	50	1	0	0	0
558	0	1	3	2	3	25	1	0	0	0
559	0	2	6	2	3	70	1	0	0	0
560	0	2	3	2	3	50	1	0	0	0

561	0	2	4	2	3	90	1	0	0	0
562	0	3	3	2	3	35	0	1	0	0
563	0	3	3	2	3	45	1	1	0	1
564	0	1	6	2	2	70	1	0	0	0
565	0	3	3	2	2	75	1	0	0	0
566	0	1	1	1	3	50	1	0	0	0
567	0	2	3	3	3	70	1	0	0	0
568	0	2	3	3	3	70	1	1	0	0
569	0	2	4	2	2	80	1	0	1	0
570	0	2	5	2	2	50	1	0	0	0
571	1	2	3	2	4	70	1	0	1	0
572	0	1	4	2	1	85	1	0	0	0
573	0	3	3	2	3	65	0	0	0	1
574	0	3	2	1	2	90	0	0	0	1
575	0	2	3	2	3	50	0	0	0	0
576	0	1	3	2	1	90	1	0	0	0
577	0	1	2	1	1	100	1	0	0	0
578	0	3	4	2	3	30	1	0	0	0
579	0	2	3	1	2	50	1	0	1	0
580	0	0	3	2	3	55	1	0	0	0
581	0	1	3	2	3	55	1	0	0	0
582	0	3	3	2	2	50	0	0	0	0
583	0	2	3	2	2	70	0	0	0	0
584	0	4	3	1	2	50	1	0	0	0
585	0	2	3	2	3	50	1	1	0	0

586	0	1	4	2	2	70	1	0	0	0
587	0	0	7	2	3	30	1	0	0	0
588	0	1	3	1	3	50	1	0	0	0
589	0	3	6	2	3	50	1	0	0	1
590	0	2	3	2	2	75	0	0	0	0
591	1	3	1	2	4	50	0	0	1	0
592	0	0	0	2	3	100	0	0	0	0
593	0	1	1	2	2	50	1	0	0	1
594	0	1	5	2	2	90	1	0	0	1
595	0	3	5	2	2	50	1	0	0	0
596	0	3	5	2	2	60	1	0	1	0
597	1	3	2	2	3	55	1	0	0	0
598	0	2	2	2	3	70	1	0	0	0
599	0	0	0	2	2	70	1	0	0	0
600	1	2	0	2	2	90	1	0	0	0
601	0	1	1	2	2	50	1	0	1	0
602	0	3	0	1	3	25	1	0	0	1
603	0	1	0	2	2	30	1	0	1	1
604	0	0	1	1	2	50	1	0	0	0
605	1	0	0	2	2	70	1	0	0	0
606	0	3	3	2	2	70	1	0	0	0
607	0	2	3	2	2	50	1	0	0	0
608	0	0	6	0	1	100	0	0	0	1
609	1	2	3	2	3	50	1	0	0	0
610	1	3	1	1	3	35	1	0	0	0

611	0	1	5	2	2	50	1	0	1	0
612	0	4	3	2	5	90	1	0	0	0
613	0	2	3	2	2	75	1	0	0	1
614	1	2	2	1	3	50	1	0	0	0
615	0	1	4	2	3	80	1	0	0	0
616	0	2	3	2	3	40	1	1	1	0
617	0	1	4	2	1	70	1	0	1	0
618	0	1	2	2	2	90	1	0	0	0
619	0	1	5	2	2	55	1	0	0	0
620	0	4	2	2	3	15	1	1	1	1
621	0	1	3	2	3	70	1	0	1	0
622	0	2	7	2	2	80	1	0	0	0
623	0	2	5	2	2	5	1	0	0	0
624	0	1	2	2	2	60	1	0	0	0
625	0	4	2	2	2	40	1	1	1	1
626	0	3	6	2	2	50	1	0	0	0
627	0	1	3	2	3	30	1	0	0	0
628	0	3	3	2	3	30	0	1	1	0
629	0	2	2	1	2	85	1	0	0	0
630	0	1	4	1	2	80	1	0	0	0
631	0	3	3	2	2	90	1	0	0	1
632	0	4	4	2	1	50	1	0	0	0
633	0	1	3	2	2	70	1	0	1	1
634	0	1	2	2	2	50	1	0	0	0
635	0	1	5	2	1	100	1	0	0	0

636	0	3	2	2	3	50	1	0	0	0
637	0	3	2	3	1	70	1	0	1	1
638	0	2	6	2	4	80	1	0	0	0
639	0	2	3	2	2	60	1	0	0	0
640	0	0	0	3	1	65	0	0	0	0
641	0	2	8	2	1	95	1	0	0	0
642	0	1	1	2	1	80	1	0	0	0
643	0	2	2	2	1	55	1	0	0	0
644	0	0	0	2	3	100	1	0	0	0
645	0	1	2	2	1	95	1	1	0	0
646	0	3	6	2	2	50	1	0	1	1
647	1	3	5	2	2	70	1	0	1	0
648	0	3	5	2	2	40	1	1	1	0
649	0	2	3	2	2	30	1	0	0	0
650	0	1	3	2	2	100	1	0	0	0
651	0	1	7	2	2	70	1	0	1	0
652	0	1	3	2	3	80	1	0	0	0
653	0	3	3	2	3	30	1	1	0	0
654	0	2	5	2	2	35	1	0	0	0
655	0	0	2	2	3	80	1	0	0	0
656	0	2	4	2	2	75	1	0	0	0
657	0	0	3	2	2	95	1	0	0	0
658	0	1	5	2	2	90	1	0	0	0
659	0	2	6	2	2	65	1	0	0	0
660	1	3	5	2	3	55	1	0	0	0

661	0	2	2	2	2	85	1	0	0	0
662	0	1	3	2	2	100	1	0	0	0
663	0	2	5	2	3	20	1	0	0	0
664	0	2	3	2	2	40	1	0	0	0
665	0	0	3	2	2	50	1	0	0	0
666	0	3	3	2	3	60	1	0	0	0
667	0	2	4	2	2	85	1	0	0	0
668	0	1	3	2	2	45	1	0	0	0
669	0	1	6	2	2	65	1	1	0	0
670	0	3	3	2	2	60	1	0	0	1
671	0	1	1	2	2	60	1	1	0	0
672	1	0	2	2	2	90	1	1	0	0
673	0	3	3	2	2	80	1	0	0	0
674	1	0	2	2	2	70	1	0	0	0
675	0	1	4	4	2	100	1	0	0	1
676	1	0	3	2	2	75	1	0	0	0
677	0	2	3	2	2	80	1	0	0	0
678	0	3	3	2	3	70	1	0	0	0
679	0	1	1	2	2	100	1	0	0	0
680	0	1	3	2	2	85	1	0	1	0
681	0	1	6	2	2	50	1	0	0	0
682	0	3	2	2	1	35	1	0	0	0
683	0	2	2	2	2	60	1	0	0	1
684	0	1	5	2	4	50	1	0	0	0
685	0	1	6	2	1	70	1	0	0	0

686	0	1	5	2	2	70	1	0	0	0
687	0	2	5	2	3	50	1	0	0	0
688	0	1	5	2	2	80	1	0	0	0
689	0	1	3	2	2	85	1	0	1	1
690	1	3	2	3	2	50	1	0	0	0
691	0	3	7	2	2	100	1	0	0	0
692	1	2	6	2	3	45	1	0	0	0
693	0	2	2	2	2	75	1	0	1	0
694	0	3	6	3	4	30	1	0	0	0
695	0	3	2	3	3	65	1	0	0	0
696	0	2	6	2	2	100	1	0	1	0
697	0	1	4	4	1	95	0	0	0	0
698	0	1	3	2	2	100	1	0	1	1
699	0	3	3	2	2	45	1	0	0	0
700	0	3	4	3	1	70	1	0	0	1
701	0	2	3	2	2	65	1	0	0	1
702	1	4	4	3	2	70	1	0	0	1
703	0	2	5	2	2	70	1	0	0	0
704	0	2	3	2	2	85	1	0	0	0
705	0	0	3	2	2	75	1	0	0	0
706	0	2	2	2	2	40	1	0	1	1
707	0	2	6	2	1	85	1	0	0	0
708	0	3	3	2	1	55	1	0	0	0
709	0	3	4	2	1	90	1	1	0	1
710	0	3	6	2	3	95	1	1	1	0

711	0	0	2	1	2	55	1	1	0	0
712	0	3	5	2	3	65	1	1	1	1
713	0	1	3	2	1	55	1	1	0	0
714	1	3	3	2	3	35	1	0	0	1
715	0	1	3	2	1	75	1	0	0	0
716	0	3	4	3	3	50	1	0	0	0
717	0	2	3	2	1	90	1	0	0	0
718	0	0	7	2	1	100	1	1	1	0
719	0	3	4	2	1	65	1	0	0	0
720	0	1	4	2	1	60	1	0	0	1
721	0	2	3	2	1	50	1	0	0	1
722	0	3	3	2	3	70	1	1	0	0
723	0	3	3	2	1	90	1	0	0	0
724	0	1	3	2	1	90	1	0	0	1
725	0	1	2	3	2	85	1	0	0	0
726	0	2	4	2	2	100	1	0	1	0
727	0	2	4	2	3	50	1	0	0	1
728	0	2	5	2	1	90	1	0	0	0
729	0	3	4	2	2	50	1	0	0	1
730	0	1	3	2	2	85	1	0	0	0
731	0	3	6	5	1	35	0	0	0	1
732	0	2	6	2	5	80	1	0	0	0
733	1	2	3	2	2	80	1	0	0	1
734	0	3	4	2	1	60	1	0	1	0
735	0	2	6	2	2	70	1	0	0	1

736	0	2	5	2	2	100	0	0	1	0
737	0	2	4	1	2	75	1	0	1	0
738	0	2	5	3	1	80	1	0	0	1
739	0	1	2	2	1	75	1	0	1	0
740	0	1	2	2	1	50	1	1	1	0
741	0	2	4	2	1	100	1	0	0	0
742	0	3	5	2	1	80	1	1	0	0
743	0	2	3	2	1	60	1	1	0	0
744	0	3	0	1	1	25	0	0	1	1
745	0	1	0	2	1	100	0	0	0	0
746	0	4	4	2	1	40	1	1	0	0
747	0	1	3	2	2	100	1	0	1	0
748	0	1	6	2	1	85	1	0	0	1
749	0	2	2	2	1	30	1	0	1	0
750	0	1	5	2	2	85	1	0	0	0
751	0	1	4	2	2	100	1	0	0	0
752	0	3	1	2	2	5	1	0	0	0
753	0	2	5	2	2	85	1	0	1	0
754	0	1	4	2	1	100	1	1	1	0
755	1	0	4	2	3	85	1	0	0	0
756	0	2	1	2	2	70	1	0	0	0
757	1	0	0	1	2	55	1	0	0	0
758	0	0	0	0	2	100	0	0	0	0
759	0	3	4	2	3	60	1	0	0	1
760	0	4	4	2	3	20	1	1	0	0

761	0	2	0	0	1	95	0	0	0	0
762	0	1	3	2	2	90	1	0	0	1
763	0	3	0	0	2	0	0	0	1	0
764	0	2	4	2	2	70	1	0	0	1
765	0	3	5	1	1	100	1	0	0	0
766	0	2	1	1	3	70	0	1	0	0
767	0	0	0	0	2	100	0	0	1	0
768	0	3	4	2	2	40	1	0	0	1
769	0	1	1	1	2	50	1	0	0	0
770	0	1	5	2	1	85	1	0	0	0
771	0	1	2	2	1	70	1	0	1	1
772	0	1	3	2	2	90	1	0	0	0
773	0	1	0	0	1	80	0	0	0	0
774	0	2	4	2	2	65	1	0	0	0
775	0	1	4	2	3	85	1	0	0	0
776	0	1	0	0	1	100	0	0	0	0
777	0	3	3	2	2	95	1	1	0	1
778	0	1	4	2	1	90	1	0	0	0
779	0	2	4	2	1	55	1	1	1	0
780	0	2	4	2	2	75	1	0	0	0
781	0	3	4	2	1	95	1	1	0	1
782	0	1	4	2	1	85	1	1	0	0
783	0	1	4	3	1	60	1	0	1	0
784	1	2	4	3	1	90	1	0	0	0
785	0	3	6	2	1	50	1	0	0	0

786	0	3	6	0	2	45	1	0	0	1
787	0	3	4	2	1	60	1	0	1	0
788	0	1	0	0	1	100	1	0	1	0
789	0	3	4	2	1	80	1	0	0	1
790	0	4	4	2	4	60	0	0	0	0
791	0	2	0	0	2	45	0	0	0	0
792	0	3	6	2	2	45	1	0	0	0
793	1	2	3	2	3	95	1	0	0	0
794	0	4	5	2	2	55	1	1	1	0
795	0	2	5	2	3	60	1	0	0	1
796	0	3	4	2	3	85	1	0	1	0
797	0	1	4	2	1	75	1	0	1	0
798	0	2	4	2	1	75	1	0	0	0

number	Bloating	edema	Anorexia	Sleep disorder	Constipation	Fatigue	total symptoms number
1	0	0	0	0	0	0	4
2	1	0	0	0	0	1	8
3	0	0	0	0	1	1	6
4	0	0	1	1	1	0	8
5	0	0	0	1	0	1	6
6	0	0	0	0	0	1	8
7	0	0	1	1	1	0	11
8	1	0	1	1	1	1	9

9	0	0	0	0	0	0	8
10	0	0	0	0	0	1	6
11	0	0	0	0	1	0	9
12	0	0	1	1	0	0	5
13	0	0	0	0	0	0	5
14	0	0	0	0	0	1	4
15	0	0	0	1	1	0	4
16	0	0	0	0	0	0	6
17	0	0	1	0	1	0	9
18	1	0	1	1	0	1	11
19	0	0	1	1	0	0	10
20	0	0	0	0	0	0	5
21	0	1	1	1	1	0	11
22	1	1	1	1	1	0	10
23	0	0	1	1	0	0	6
24	0	0	1	1	0	0	4
25	0	1	1	0	1	1	11
26	0	0	0	1	0	0	5
27	0	0	1	0	1	1	9
28	0	0	1	1	0	1	13
29	1	0	1	0	1	0	8
30	0	0	1	1	0	1	9
31	0	0	1	1	0	0	4
32	0	0	1	1	0	1	8
33	0	0	1	0	0	0	5

34	0	0	1	0	1	1	7
35	0	0	1	0	0	0	5
36	0	0	0	0	1	0	5
37	1	0	1	1	0	1	11
38	0	1	1	1	1	1	12
39	0	1	1	1	0	0	7
40	0	0	0	0	0	0	5
41	1	0	0	0	0	0	3
42	1	1	1	1	0	1	9
43	1	0	0	0	1	1	6
44	1	0	1	1	0	1	9
45	0	0	1	1	0	1	10
46	0	0	1	0	0	0	6
47	1	1	0	0	1	0	8
48	0	0	0	0	0	0	5
49	0	0	1	1	1	0	5
50	0	1	1	1	0	0	6
51	0	0	1	0	1	0	13
52	0	0	1	0	0	0	4
53	0	0	1	1	1	1	8
54	0	0	1	1	0	0	5
55	0	0	1	1	1	1	11
56	0	0	0	0	1	1	8
57	1	0	1	1	1	1	9
58	0	1	0	0	0	1	8

59	0	1	0	0	0	0	3
60	0	0	0	0	0	0	3
61	1	0	0	1	0	1	6
62	0	0	0	0	0	1	9
63	1	0	1	0	0	1	10
64	0	0	1	1	0	1	9
65	0	0	1	1	0	1	9
66	0	0	0	0	1	0	5
67	1	0	0	0	0	0	9
68	0	0	0	0	1	0	2
69	0	0	1	1	0	1	10
70	1	1	0	1	0	1	14
71	0	0	0	0	0	0	2
72	0	1	0	0	0	0	1
73	0	0	0	0	0	0	2
74	0	0	0	0	1	0	4
75	1	1	1	0	1	1	11
76	1	0	1	0	1	1	9
77	0	1	0	0	0	0	5
78	0	1	1	1	0	1	13
79	0	0	0	1	0	1	9
80	0	0	1	0	1	0	7
81	0	0	0	1	1	0	4
82	1	0	0	1	0	1	12
83	1	0	1	0	0	1	7

84	0	0	0	0	1	0	3
85	1	0	1	1	0	1	12
86	0	0	1	0	0	1	6
87	0	0	0	0	1	1	5
88	0	0	0	1	0	1	4
89	0	0	1	0	1	0	7
90	0	0	0	1	1	1	10
91	1	0	0	1	1	1	9
92	1	0	0	0	0	0	2
93	1	0	1	1	1	0	7
94	0	0	0	0	0	0	2
95	0	0	0	1	1	0	3
96	0	0	0	1	1	1	8
97	0	0	1	1	0	1	9
98	0	1	0	0	1	0	6
99	0	1	1	1	1	1	9
100	0	0	1	1	0	1	6
101	1	1	0	0	1	0	6
102	0	0	0	1	0	0	2
103	0	1	1	1	1	0	7
104	1	0	0	1	1	1	12
105	0	0	1	0	1	1	4
106	0	0	1	0	1	1	8
107	0	0	1	1	1	1	7
108	0	0	1	1	0	1	9

109	0	1	1	1	1	1	10
110	1	0	0	0	1	1	6
111	0	1	0	1	0	0	3
112	1	0	0	0	0	0	3
113	0	0	0	1	1	1	7
114	0	0	1	1	0	1	9
115	1	0	1	1	1	1	9
116	0	0	0	1	1	1	7
117	0	0	1	1	0	0	3
118	1	0	0	1	1	0	7
119	0	0	0	0	0	0	3
120	0	0	0	0	0	0	2
121	0	0	0	0	0	0	1
122	0	0	0	0	0	1	7
123	0	0	0	0	0	0	1
124	0	1	1	0	0	0	10
125	0	0	0	0	0	0	1
126	1	1	1	0	0	1	8
127	0	0	0	0	0	0	5
128	0	0	0	0	1	1	4
129	0	0	0	0	0	1	4
130	0	0	0	0	0	0	2
131	0	0	1	0	0	1	5
132	0	0	0	0	1	0	6
133	0	0	0	0	1	1	4

134	1	0	1	0	0	1	7
135	1	0	0	0	0	0	2
136	0	0	0	1	1	0	7
137	0	0	0	0	0	0	4
138	0	0	1	0	0	0	6
139	0	0	1	1	0	0	6
140	0	0	0	1	1	1	7
141	0	0	1	1	0	1	6
142	0	0	1	1	0	0	3
143	0	0	0	0	0	0	7
144	1	0	1	1	0	0	5
145	0	0	0	0	0	0	2
146	0	0	0	1	0	0	2
147	0	0	0	0	1	0	5
148	0	0	0	0	0	0	2
149	0	0	1	0	1	1	10
150	0	0	1	1	0	1	9
151	0	0	0	0	0	0	3
152	0	0	1	0	0	0	3
153	0	0	0	0	0	0	3
154	0	0	1	0	1	1	7
155	0	0	0	0	0	0	2
156	0	0	0	0	0	0	2
157	0	0	1	0	0	0	4
158	0	0	1	0	0	0	2

159	0	0	0	0	0	0	1
160	0	0	1	1	0	1	7
161	0	0	0	0	0	0	2
162	0	0	1	1	1	0	9
163	1	0	0	0	1	0	3
164	0	0	1	0	0	1	7
165	0	0	1	1	1	1	6
166	1	0	0	1	0	1	9
167	1	0	1	1	1	1	7
168	0	0	1	1	0	0	3
169	0	0	0	0	0	1	3
170	0	0	0	1	1	0	4
171	0	0	0	0	0	0	1
172	0	0	1	1	0	0	4
173	0	0	1	1	0	0	7
174	0	0	1	1	0	0	5
175	0	0	1	1	0	0	3
176	0	0	1	0	0	0	3
177	0	0	0	1	1	0	3
178	0	0	1	0	0	1	7
179	0	1	1	0	1	0	8
180	0	0	0	0	0	0	1
181	0	0	0	0	0	0	1
182	0	0	0	0	1	0	2
183	0	0	0	0	1	0	4

184	0	1	0	0	0	0	2
185	0	0	0	0	1	0	2
186	1	0	1	1	0	1	7
187	0	0	0	0	0	0	3
188	0	0	0	0	0	0	3
189	0	0	0	0	1	0	3
190	0	0	0	0	0	0	3
191	0	0	1	1	1	0	7
192	0	0	0	0	0	0	2
193	0	0	1	0	0	0	3
194	0	0	0	0	0	0	3
195	0	0	0	1	0	0	3
196	0	0	0	0	0	0	2
197	0	0	0	0	0	0	2
198	0	0	0	0	0	0	1
199	1	0	1	1	1	1	10
200	0	0	0	0	0	0	1
201	0	0	0	0	0	0	2
202	0	0	0	0	0	0	5
203	0	0	1	1	1	1	6
204	0	0	1	1	0	1	6
205	0	0	1	0	0	0	3
206	1	0	0	0	0	0	4
207	0	0	0	1	0	1	6
208	0	0	0	0	0	1	3

209	0	0	1	1	0	1	5
210	0	0	0	0	0	0	1
211	0	0	1	1	0	1	6
212	0	0	1	1	0	1	5
213	1	0	0	1	0	0	5
214	0	0	1	1	0	1	5
215	0	0	1	1	1	1	7
216	0	0	1	1	1	1	7
217	0	0	1	1	0	1	5
218	0	0	1	1	1	0	6
219	0	0	0	0	1	0	3
220	0	0	1	1	1	1	6
221	1	0	0	0	0	0	3
222	0	0	0	0	0	0	1
223	1	0	0	1	1	1	6
224	0	0	1	1	1	1	9
225	0	0	0	1	0	0	2
226	0	0	1	1	1	0	6
227	0	0	0	0	1	0	3
228	0	0	1	0	0	0	3
229	0	0	0	0	0	0	2
230	0	0	0	0	1	0	3
231	0	1	0	1	0	0	3
232	0	0	0	0	0	0	1
233	0	0	0	0	0	0	1

234	0	0	0	1	1	1	7
235	0	0	0	0	0	0	0
236	0	1	0	1	1	1	7
237	1	0	0	0	0	0	5
238	1	0	0	1	0	0	6
239	0	0	0	0	0	1	3
240	0	0	0	0	0	0	1
241	0	0	1	0	0	0	4
242	0	1	0	0	0	1	6
243	0	0	0	0	0	0	1
244	1	0	1	1	0	0	7
245	0	0	0	0	0	0	1
246	0	0	0	0	0	0	5
247	0	0	1	1	0	1	6
248	0	0	0	0	0	0	2
249	0	0	0	0	0	0	2
250	0	0	0	0	0	0	1
251	0	0	0	0	0	0	3
252	0	0	0	0	0	1	4
253	1	1	1	1	0	1	9
254	0	0	0	0	0	0	5
255	0	0	0	0	0	0	3
256	1	1	1	0	1	0	5
257	0	0	1	0	1	1	6
258	0	0	1	1	0	0	3

259	0	0	1	1	0	0	3
260	0	1	1	0	0	1	6
261	0	0	0	0	0	0	2
262	0	1	0	0	0	0	3
263	0	0	1	1	1	1	6
264	0	0	1	0	0	1	9
265	0	0	0	0	0	1	2
266	0	0	1	0	0	1	6
267	0	0	0	0	0	0	1
268	0	0	0	0	0	0	4
269	0	0	0	1	0	0	2
270	0	0	0	0	1	0	2
271	0	0	0	1	0	1	4
272	0	1	1	1	1	1	8
273	0	0	0	1	0	1	6
274	0	0	1	1	0	0	4
275	0	1	1	1	1	1	9
276	0	0	1	0	0	1	4
277	0	0	0	0	0	1	3
278	0	0	1	1	1	1	6
279	0	0	1	1	0	1	6
280	0	0	1	1	0	0	4
281	0	0	0	1	0	0	4
282	0	0	1	1	0	1	5
283	0	0	1	1	0	1	5

284	0	0	1	1	0	1	5
285	0	0	0	0	0	0	1
286	0	0	1	1	0	1	7
287	0	0	0	0	1	0	2
288	0	0	0	0	1	0	3
289	0	0	0	0	0	0	2
290	0	0	0	0	1	0	2
291	0	0	1	0	1	0	4
292	0	0	1	1	1	1	9
293	0	0	0	1	1	0	3
294	0	0	0	1	0	1	3
295	0	0	0	1	0	1	7
296	0	0	1	0	1	0	3
297	0	0	0	0	0	0	3
298	0	0	0	1	0	0	2
299	0	0	1	0	0	0	4
300	0	1	1	0	0	0	5
301	0	0	0	0	0	0	1
302	0	0	1	1	1	1	6
303	0	0	0	0	0	0	2
304	0	0	1	1	0	1	4
305	0	0	0	0	1	0	2
306	0	0	1	1	1	1	5
307	0	0	1	1	0	0	5
308	0	0	0	0	0	1	4

309	0	0	1	1	0	0	6
310	0	0	0	1	0	0	3
311	0	0	1	1	0	0	4
312	0	0	1	1	0	0	4
313	0	0	1	1	0	0	3
314	0	0	1	0	0	0	2
315	0	0	0	0	0	0	2
316	1	0	1	1	1	1	8
317	0	0	0	1	0	1	5
318	0	0	1	1	0	1	5
319	0	0	0	0	0	0	1
320	0	0	0	0	0	0	1
321	0	0	1	1	0	0	3
322	0	0	1	1	1	0	5
323	0	0	0	0	0	0	1
324	0	0	1	1	0	1	6
325	0	0	0	0	0	0	3
326	0	0	1	1	0	0	6
327	0	0	0	0	0	0	1
328	0	0	0	1	0	0	2
329	0	0	0	0	0	0	2
330	0	0	0	0	0	0	1
331	0	0	1	0	0	1	5
332	0	0	0	0	0	0	3
333	0	0	1	1	1	0	4

334	0	0	0	0	1	0	2
335	0	0	1	0	0	0	2
336	0	1	0	0	1	0	6
337	0	0	0	0	0	0	2
338	0	0	1	1	0	0	3
339	0	0	1	1	1	1	10
340	0	0	1	0	0	1	5
341	1	0	1	1	1	1	7
342	0	0	0	0	0	0	3
343	0	1	1	0	0	0	3
344	0	0	0	0	0	0	4
345	0	0	1	0	0	1	5
346	0	0	1	0	0	0	2
347	0	0	1	1	0	1	5
348	0	0	0	0	0	0	3
349	0	0	0	0	0	0	3
350	0	0	1	1	0	0	6
351	0	0	1	1	0	1	7
352	0	0	0	0	0	1	2
353	1	0	1	1	0	0	5
354	1	1	1	1	1	1	10
355	0	1	1	1	1	1	7
356	0	0	1	1	0	1	6
357	0	0	0	0	0	0	2
358	0	0	1	1	0	1	6

359	0	0	1	1	1	1	6
360	1	0	1	1	1	1	9
361	0	1	1	1	0	1	7
362	0	0	1	1	0	1	6
363	0	0	1	1	1	1	7
364	0	0	1	1	0	0	6
365	0	0	1	1	1	0	5
366	0	0	1	1	1	1	7
367	0	1	0	0	0	0	2
368	0	0	1	1	1	1	8
369	0	0	1	1	0	0	4
370	0	0	0	0	0	0	1
371	0	0	0	0	0	0	2
372	0	0	1	1	1	1	6
373	0	0	0	0	0	0	1
374	0	0	1	1	0	1	7
375	1	0	1	1	1	1	10
376	0	0	0	0	0	0	1
377	0	0	1	1	1	1	6
378	0	0	0	0	0	0	1
379	1	0	1	0	0	1	7
380	0	0	0	1	0	1	4
381	0	0	0	0	1	0	2
382	0	0	1	1	1	0	7
383	0	1	1	1	0	0	6

384	0	0	0	0	1	0	2
385	0	0	1	1	0	1	7
386	0	0	1	1	0	1	6
387	0	0	0	0	0	0	1
388	0	0	1	1	0	1	5
389	0	0	1	0	0	1	5
390	0	0	0	0	0	0	1
391	0	0	0	0	0	0	3
392	0	0	0	0	0	0	4
393	0	0	1	1	0	0	5
394	0	0	1	0	0	1	5
395	0	0	0	0	0	0	2
396	0	0	0	0	1	0	4
397	0	0	0	0	0	0	2
398	0	1	0	0	0	0	2
399	1	0	1	0	0	1	9
400	0	0	1	0	0	0	6
401	0	0	1	1	0	0	3
402	0	0	0	0	0	0	1
403	0	0	1	1	0	0	8
404	0	0	0	1	0	0	2
405	0	0	0	0	0	0	2
406	0	0	1	1	0	0	4
407	0	0	1	1	0	0	5
408	0	0	0	0	0	0	2

409	0	1	0	0	0	0	3
410	0	0	1	1	0	0	6
411	0	0	0	0	0	0	2
412	0	0	0	0	0	0	2
413	0	0	0	0	0	0	2
414	0	0	0	0	0	0	2
415	0	0	1	1	0	0	3
416	0	0	1	0	1	0	4
417	0	0	0	0	0	0	2
418	0	0	0	0	1	0	2
419	0	0	0	0	0	0	2
420	0	0	1	1	0	0	3
421	1	0	1	1	0	0	5
422	0	0	1	0	0	0	3
423	0	0	1	1	0	1	6
424	0	0	0	0	1	0	2
425	0	0	1	0	1	0	3
426	0	0	0	1	0	0	2
427	0	0	0	0	0	0	3
428	0	0	0	0	0	0	4
429	1	0	0	0	0	0	3
430	1	0	1	1	1	1	7
431	0	1	1	0	0	0	6
432	1	0	1	1	0	1	6
433	0	0	1	0	1	0	4

434	0	0	1	1	0	1	6
435	0	0	0	0	1	0	2
436	0	0	0	0	1	0	2
437	0	0	0	0	0	0	3
438	0	0	0	1	1	0	5
439	0	0	0	0	0	0	1
440	0	0	0	1	1	0	4
441	0	0	1	0	1	0	4
442	0	0	1	1	1	1	9
443	0	0	0	0	0	1	6
444	0	0	1	0	0	1	4
445	0	0	0	0	0	0	1
446	0	0	0	0	0	0	1
447	0	0	0	0	0	0	1
448	0	0	1	1	0	1	5
449	0	0	1	1	0	1	5
450	0	0	0	0	1	0	3
451	1	0	0	0	0	0	2
452	1	0	0	0	0	0	4
453	0	0	1	1	0	0	7
454	0	0	1	1	1	0	5
455	0	0	0	0	0	0	1
456	1	0	0	0	1	0	4
457	0	0	0	0	0	0	1
458	0	0	1	1	0	1	5

459	0	0	0	0	1	0	2
460	0	0	0	0	1	0	2
461	0	0	0	0	1	0	2
462	0	0	0	1	0	1	5
463	0	0	0	0	1	0	3
464	0	0	0	0	1	0	2
465	0	0	0	1	1	0	4
466	0	0	0	0	0	0	0
467	0	0	0	0	0	0	0
468	0	0	0	0	0	0	1
469	0	0	0	1	1	0	5
470	0	0	1	0	1	0	3
471	0	0	0	0	0	0	2
472	0	0	1	1	1	0	5
473	0	1	0	0	0	0	5
474	0	0	0	0	0	0	3
475	0	0	0	0	0	0	1
476	0	0	0	0	0	0	1
477	0	0	0	0	1	0	1
478	0	0	0	0	0	0	1
479	0	0	1	1	0	0	4
480	0	0	0	0	0	0	1
481	1	0	1	0	0	1	7
482	1	1	0	1	0	0	4
483	0	0	0	1	0	1	8

484	0	0	0	0	0	0	2
485	0	0	0	0	0	0	1
486	0	1	0	0	0	0	3
487	0	0	1	0	0	0	2
488	0	0	0	0	0	0	1
489	0	0	0	0	0	0	4
490	0	0	0	0	0	0	0
491	0	0	0	0	0	1	5
492	0	0	0	0	1	0	4
493	0	0	1	0	1	0	3
494	0	1	0	0	0	0	4
495	0	0	0	0	0	0	2
496	0	0	0	0	1	0	2
497	0	0	0	0	0	0	1
498	0	0	0	0	0	0	1
499	0	0	0	0	0	0	1
500	0	0	0	0	1	0	2
501	0	1	0	0	0	0	3
502	0	0	0	0	0	0	1
503	0	0	0	1	0	0	4
504	0	0	0	0	1	0	2
505	0	0	1	1	0	0	4
506	0	0	0	0	0	1	4
507	0	0	0	0	0	0	2
508	0	0	0	0	0	0	2

509	0	0	0	0	0	1	2
510	0	0	0	0	0	0	1
511	0	0	0	0	0	0	0
512	0	0	0	0	0	0	0
513	0	0	0	0	1	0	3
514	0	0	1	0	0	0	4
515	0	0	1	0	1	0	3
516	0	0	0	0	0	0	1
517	0	0	1	1	0	0	2
518	0	0	0	0	0	1	4
519	0	0	1	0	0	1	4
520	0	0	0	0	0	0	2
521	0	0	1	1	1	1	7
522	0	0	1	1	1	1	8
523	1	1	1	1	1	1	9
524	0	0	0	0	0	0	4
525	0	0	0	0	0	0	1
526	0	0	1	1	1	1	6
527	0	0	1	1	1	0	8
528	0	0	1	0	0	0	2
529	0	0	0	0	0	0	1
530	0	0	0	0	0	0	1
531	0	0	0	0	0	0	3
532	0	0	0	1	1	0	5
533	0	0	0	0	0	0	0

534	0	0	0	0	1	0	3
535	0	0	0	0	0	0	4
536	0	0	1	1	0	0	4
537	0	0	1	1	0	0	3
538	0	0	0	0	0	0	1
539	0	0	0	0	1	0	3
540	0	1	0	0	0	0	3
541	0	0	0	0	0	0	3
542	0	0	0	0	0	0	1
543	0	0	1	0	0	0	3
544	0	0	0	0	1	0	6
545	0	0	1	0	1	0	5
546	0	0	0	0	0	0	4
547	0	0	1	1	1	0	5
548	0	0	1	0	0	1	3
549	0	0	1	1	0	0	4
550	0	0	0	0	0	1	2
551	0	0	0	0	0	0	1
552	1	0	1	0	1	1	7
553	0	0	0	0	0	0	1
554	0	0	0	0	0	0	2
555	0	0	0	0	1	0	3
556	0	0	1	0	0	0	3
557	0	1	1	0	0	1	7
558	0	0	1	1	0	0	3

559	0	0	1	1	0	1	8
560	0	0	0	1	0	0	4
561	0	0	0	0	0	0	1
562	0	1	0	0	0	0	4
563	1	1	0	0	0	1	9
564	0	0	0	0	0	0	3
565	0	0	0	0	1	0	4
566	1	0	0	0	0	0	2
567	0	0	0	0	0	0	1
568	0	1	0	0	1	0	4
569	0	0	0	0	0	1	8
570	0	0	1	1	0	1	5
571	0	0	1	1	1	1	9
572	0	0	0	0	0	0	1
573	0	0	0	0	0	0	5
574	0	0	0	0	0	0	2
575	0	0	0	0	0	0	0
576	0	0	0	0	0	0	1
577	0	0	0	0	0	0	1
578	1	0	0	0	0	0	2
579	0	0	0	0	1	0	7
580	0	0	0	0	0	1	5
581	0	0	0	0	0	0	1
582	0	0	0	0	0	0	1
583	0	0	0	0	0	0	0

584	0	0	0	0	0	1	2
585	0	0	0	0	1	0	3
586	0	0	0	0	0	0	2
587	0	0	0	0	1	0	3
588	0	0	0	0	0	0	1
589	0	0	1	0	0	1	6
590	0	0	1	1	0	0	2
591	0	1	0	0	0	0	4
592	0	0	0	0	0	0	0
593	0	0	1	1	0	0	4
594	0	0	1	1	0	0	5
595	1	1	0	0	0	0	5
596	0	0	0	0	0	0	3
597	0	0	0	0	0	0	4
598	0	0	1	1	0	0	4
599	1	0	1	1	0	0	4
600	0	1	0	0	0	1	5
601	0	0	0	0	0	1	3
602	0	0	1	0	0	1	6
603	0	0	1	1	1	0	6
604	0	0	0	0	0	0	1
605	1	0	0	0	0	0	2
606	0	0	1	1	0	0	3
607	0	0	1	1	0	0	3
608	0	0	0	0	0	0	1

609	1	0	0	0	1	1	9
610	0	1	0	0	0	0	2
611	0	0	0	0	0	1	5
612	0	0	1	1	1	0	4
613	1	0	1	0	1	0	6
614	0	0	0	0	0	0	1
615	1	0	1	0	0	1	4
616	0	0	1	1	1	1	9
617	1	0	0	0	0	0	4
618	0	0	0	0	1	1	3
619	0	0	0	0	1	0	3
620	1	0	1	0	0	0	9
621	0	0	0	0	0	0	2
622	0	0	0	0	1	0	2
623	1	0	0	1	0	1	6
624	0	0	0	0	0	1	3
625	1	0	1	0	0	1	9
626	0	0	1	0	0	0	4
627	0	0	0	0	1	0	4
628	0	1	1	0	1	0	5
629	0	1	0	1	0	0	6
630	0	0	0	0	0	0	1
631	0	1	1	0	0	1	6
632	0	0	1	0	0	1	3
633	1	0	1	0	1	0	8

634	0	0	0	0	0	0	1
635	0	0	0	0	0	0	3
636	1	0	0	1	1	0	6
637	0	0	0	0	0	0	5
638	0	1	0	1	0	0	4
639	0	0	0	0	0	0	1
640	0	0	0	0	0	0	0
641	0	0	0	0	1	0	2
642	0	0	0	0	0	0	1
643	1	0	0	0	0	0	4
644	0	1	0	1	1	0	11
645	0	0	1	1	0	0	4
646	0	0	1	0	0	1	8
647	0	0	1	1	1	1	6
648	0	0	0	0	0	0	5
649	0	0	1	1	0	0	4
650	0	0	0	0	0	0	1
651	0	0	0	0	0	0	2
652	0	0	0	0	0	0	1
653	0	1	0	0	0	0	3
654	0	0	0	0	0	0	1
655	0	1	1	0	0	0	4
656	0	0	0	0	0	0	2
657	0	0	0	0	0	0	1
658	1	0	0	0	0	0	2

659	0	0	0	0	0	0	2
660	0	0	0	0	0	0	3
661	0	0	1	1	0	0	4
662	0	0	0	0	0	0	1
663	0	0	0	0	0	0	1
664	0	0	0	0	0	0	1
665	0	0	0	0	0	0	1
666	0	0	0	0	0	0	3
667	0	0	0	0	0	0	1
668	0	0	0	0	0	0	1
669	0	0	1	0	0	1	6
670	0	0	0	0	0	0	3
671	0	0	0	1	0	0	4
672	0	0	0	0	0	0	2
673	0	0	0	0	0	0	2
674	0	0	0	0	1	0	2
675	1	0	0	0	0	0	3
676	0	0	0	0	0	0	1
677	0	0	0	0	0	0	1
678	0	0	0	0	0	0	1
679	0	0	0	0	0	0	2
680	0	0	0	0	0	0	2
681	0	0	0	0	0	0	1
682	0	0	0	1	0	0	3
683	0	0	0	0	0	0	4

684	0	0	0	0	0	0	1
685	0	0	0	0	0	0	1
686	0	0	0	0	0	0	2
687	0	0	0	0	0	0	1
688	0	0	0	0	0	0	1
689	1	0	0	0	0	0	4
690	1	0	0	0	0	0	2
691	0	0	0	0	0	0	1
692	0	0	0	0	0	0	3
693	0	0	0	0	0	0	3
694	1	0	0	0	0	0	3
695	0	0	0	1	0	1	6
696	0	0	0	0	0	0	2
697	0	0	0	0	0	0	0
698	0	0	1	0	0	0	6
699	0	0	0	0	0	1	2
700	0	0	0	0	0	0	4
701	0	0	0	0	0	0	2
702	1	0	0	0	0	0	3
703	0	0	0	0	0	0	1
704	0	0	1	0	0	1	4
705	0	0	0	0	0	0	1
706	0	0	0	0	0	0	5
707	0	0	0	0	0	0	1
708	0	0	0	0	0	0	2

709	1	0	0	0	1	0	5
710	0	0	0	1	1	0	5
711	0	0	0	0	0	0	2
712	0	0	0	0	1	1	7
713	0	0	0	0	0	0	3
714	0	0	0	0	0	1	3
715	0	0	0	0	0	0	1
716	0	0	1	1	0	0	3
717	0	0	1	1	0	0	4
718	0	0	0	1	0	0	4
719	0	0	0	0	0	0	1
720	0	0	1	1	0	0	4
721	0	0	0	1	0	1	4
722	1	0	0	1	0	0	5
723	0	0	0	0	0	0	2
724	0	0	0	0	0	0	2
725	0	0	0	0	0	0	1
726	1	0	1	1	0	1	7
727	1	0	1	1	0	1	6
728	0	0	0	0	0	0	1
729	0	0	1	1	0	0	4
730	0	0	0	0	0	0	1
731	1	0	0	0	0	1	3
732	1	0	1	1	0	0	6
733	1	0	1	0	0	0	5

734	0	0	1	0	0	1	7
735	1	0	0	0	0	0	3
736	0	0	0	1	0	0	2
737	0	0	0	0	0	0	2
738	0	0	0	0	0	0	3
739	0	0	0	0	0	0	2
740	0	0	0	0	0	0	3
741	0	0	0	1	0	0	2
742	0	0	0	0	0	0	4
743	0	0	0	0	0	0	4
744	0	0	0	0	0	1	4
745	0	0	0	0	0	0	0
746	1	1	0	1	1	0	6
747	0	0	0	0	1	1	4
748	0	0	0	1	1	0	5
749	0	0	0	1	0	0	5
750	0	0	0	0	1	0	2
751	1	0	0	0	0	1	3
752	0	0	0	0	0	1	3
753	0	0	1	0	0	1	6
754	0	0	0	0	0	0	4
755	0	0	0	0	1	0	3
756	0	0	0	0	0	0	2
757	0	0	0	0	0	0	1
758	0	0	0	0	0	0	0

759	0	0	0	0	0	0	4
760	0	0	0	0	0	1	4
761	1	0	0	0	0	1	3
762	0	0	0	0	0	0	2
763	0	0	0	0	0	0	3
764	0	0	0	0	0	0	3
765	0	0	0	0	0	0	3
766	0	0	0	0	0	0	1
767	0	0	0	0	0	0	1
768	1	0	0	0	0	1	4
769	0	0	0	1	0	0	2
770	0	0	0	0	1	0	3
771	0	0	1	0	0	1	8
772	0	0	0	0	0	1	3
773	0	0	0	0	0	0	0
774	0	1	0	0	1	1	4
775	1	0	0	0	1	0	4
776	0	0	0	0	0	0	1
777	1	1	0	0	1	1	11
778	0	0	0	0	0	0	2
779	0	0	0	1	0	0	5
780	0	0	0	0	0	0	1
781	1	0	0	0	0	0	6
782	0	0	0	0	1	0	3
783	0	0	0	0	0	0	3

784	0	0	0	0	1	0	2
785	0	0	0	0	0	0	4
786	0	0	0	0	1	1	5
787	0	0	0	0	0	1	6
788	0	0	0	0	0	0	4
789	0	0	0	0	0	1	3
790	1	0	0	0	1	0	5
791	0	0	0	1	0	1	3
792	1	0	0	0	0	1	9
793	0	0	0	1	0	1	5
794	0	0	0	1	0	0	5
795	0	0	0	0	1	1	5
796	0	0	0	1	0	1	8
797	0	0	0	0	0	1	8
798	0	0	0	1	1	1	5

number	Medications for BTP onset（1intramuscular injection of hydromorphone hydrochloride；2subcutaneous morphine；3intramuscular injection of ketorolac tromethamine;4oral morphine ;5intravenous analgesia;6PCIA;7other）	intensity	breakthrough pain（0 none；1 BTP occure）	episodes	pain at discharge	average stay
1	3	6	1	4	2	8
2	0	0	0	0	0	16
3	0	0	0	0	0	4
4	2	7	1	2	2	7
5	2	8	1	3	2	12
6	3	6	1	10	2	15
7	3	6	1	6	2	33
8	2	7	1	1	1	21
9	0	0	0	0	0	4
10	0	0	0	0	0	7
11	3	6	1	2	1	7
12	0	0	0	0	0	9
13	0	0	0	0	0	8
14	3	6	1	10	2	13
15	3	6	1	2	2	6
16	2	7	1	2	2	31

17	7	6	1	4	2	12
18	0	0	0	0	0	7
19	7	6	1	2	2	2
20	0	0	0	0	0	6
21	5	6	1	1	2	6
22	2	8	1	2	2	24
23	2	5	1	3	2	8
24	6	6	1	4	3	7
25	2	5	1	5	2	14
26	0	0	0	0	0	19
27	2	7	1	6	3	18
28	2	7	1	7	2	45
29	2	7	1	8	2	12
30	2	7	1	9	3	14
31	0	0	0	0	0	14
32	0	0	0	0	0	33
33	3	6	1	10	2	4
34	3	6	1	1	1	26
35	2	5	1	2	2	7
36	2	5	1	14	2	16.5
37	0	0	0	0	0	47
38	2	7	1	15	2	11
39	0	0	0	0	0	5
40	0	0	0	0	0	26
41	0	0	0	0	0	1

42	3	6	1	3	2	14
43	3	6	1	5	2	4
44	1	7	1	6	2	9
45	0	0	0	0	0	2
46	0	0	0	0	0	4
47	4	6	1	7	2	9
48	0	0	0	0	0	10
49	3	6	1	8	2	5
50	1	7	1	8	2	18
51	0	0	0	0	0	9
52	3	6	1	9	2	8
53	0	0	0	0	0	3
54	0	0	0	0	0	6
55	2	6	1	10	2	7
56	2	5	1	2	2	11
57	5	5	1	3	2	16
58	1	7	1	4	3	8
59	1	7	1	5	3	28
60	1	7	1	5	3	12
61	2	8	1	10	3	39
62	4	6	1	6	2	6
63	2	10	1	7	3	50
64	0	0	0	0	0	8
65	1	7	1	8	2	18
66	0	0	0	0	0	20

67	3	6	1	12	3	3
68	2	5	1	11	3	7
69	2	5	1	10	2	22
70	3	6	1	2	2	16
71	7	6	1	3	2	7
72	0	0	0	0	0	7
73	2	5	1	4	3	8
74	2	5	1	6	3	8
75	0	0	0	0	0	8
76	0	0	0	0	0	1
77	2	5	1	7	3	4
78	1	7	1	8	3	9
79	0	0	0	0	0	14
80	0	0	0	0	0	12
81	2	6	1	8	3	7
82	2	6	1	5	3	7
83	0	0	0	0	0	12
84	2	6	1	4	2	8
85	1	7	1	3	2	12
86	3	6	1	6	2	15
87	1	7	1	7	3	15
88	2	5	1	1	2	8
89	7	6	1	2	2	12
90	2	6	1	3	2	26
91	0	0	0	0	0	11

92	2	6	1	4	3	12
93	3	6	1	5	2	16
94	0	0	0	0	0	4
95	3	6	1	6	2	12
96	0	0	0	0	0	11
97	2	6	1	7	3	14
98	0	0	0	0	0	30
99	3	6	1	8	2	14
100	2	6	1	9	1	15
101	2	6	1	10	2	11
102	0	0	0	0	0	15
103	2	8	1	1	4	24
104	2	6	1	2	2	14
105	2	6	1	3	3	13
106	3	6	1	4	3	18
107	3	6	1	5	2	17
108	0	0	0	0	0	21
109	0	0	0	0	0	15
110	0	0	0	0	0	12
111	2	6	1	6	3	7
112	0	0	0	0	0	11
113	2	5	1	7	2	13
114	2	6	1	7	2	22
115	2	6	1	8	3	10
116	0	0	0	0	0	12

117	2	5	1	9	2	31
118	2	6	1	10	2	10
119	0	0	0	0	0	8
120	0	0	0	0	0	19
121	0	0	0	0	0	5
122	2	6	1	10	2	30
123	2	6	1	11	2	12
124	3	6	1	2	3	10
125	0	0	0	0	0	7
126	0	0	0	0	0	14
127	0	0	0	0	0	6
128	2	5	1	5	3	39
129	0	0	0	0	0	4
130	3	6	1	6	2	18
131	0	0	0	0	0	6
132	0	0	0	0	0	9
133	0	0	0	0	0	5
134	0	0	0	0	0	12
135	4	6	1	7	2	8
136	0	0	0	0	0	9
137	2	8	1	8	2	13
138	2	5	1	9	3	32
139	1	7	1	10	2	33
140	5	5	1	1	2	6
141	0	0	0	0	0	8

142	0	0	0	0	0	9
143	1	7	1	2	2	26
144	1	7	1	1	2	4
145	2	5	1	2	2	18
146	0	0	0	0	0	7
147	0	0	0	0	0	8
148	0	0	0	0	0	6
149	3	6	1	3	2	20
150	0	0	0	0	0	18
151	0	0	0	0	0	14
152	5	5	1	2	2	11
153	0	0	0	0	0	7
154	1	7	1	3	2	28
155	0	0	0	0	0	18
156	0	0	0	0	0	18
157	0	0	0	0	0	8
158	2	5	1	4	2	16
159	4	6	1	4	2	3
160	4	6	1	3	2	5
161	0	0	0	0	0	17
162	2	6	1	2	2	3
163	0	0	0	0	0	12
164	0	0	0	0	0	14
165	2	5	1	4	2	40
166	4	6	1	5	2	31

167	3	6	1	4	3	45
168	0	0	0	0	0	10
169	2	5	1	2	2	9
170	0	0	0	0	0	15
171	0	0	0	0	0	11
172	3	6	1	4	3	14
173	2	5	1	5	2	14
174	2	5	1	5	2	6
175	2	5	1	6	2	11
176	2	5	1	1	2	1
177	2	5	1	1	2	7
178	0	0	0	0	0	9
179	5	5	1	1	2	10
180	0	0	0	0	0	6
181	0	0	0	0	0	6
182	0	0	0	0	0	4
183	4	6	1	3	2	4
184	1	7	1	3	2	10
185	0	0	0	0	0	19
186	1	7	1	7	3	32
187	0	0	0	0	0	2
188	0	0	0	0	0	2
189	6	5	1	1	2	1
190	0	0	0	0	0	4
191	0	0	0	0	0	6

192	0	0	0	0	0	7
193	0	0	0	0	0	3
194	0	0	0	0	0	3
195	2	6	1	1	2	7
196	0	0	0	0	0	14
197	0	0	0	0	0	6
198	0	0	0	0	0	6
199	2	7	1	12	3	26
200	0	0	0	0	0	8
201	0	0	0	0	0	10
202	2	6	1	2	2	6
203	3	6	1	5	1	11
204	3	6	1	5	2	13
205	3	6	1	1	2	9
206	0	0	0	0	0	12
207	2	6	1	17	2	17
208	7	6	1	3	2	10
209	0	0	0	0	0	15
210	2	5	1	1	2	7
211	0	0	0	0	0	35
212	3	6	1	3	2	22
213	2	6	1	11	2	39
214	0	0	0	0	0	7
215	0	0	0	0	0	5
216	0	0	0	0	0	16

217	2	6	1	18	2	23
218	0	0	0	0	0	6
219	0	0	0	0	0	13
220	7	6	1	21	2	35
221	0	0	0	0	0	10
222	0	0	0	0	0	11
223	3	6	1	42	2	22
224	2	9	1	21	2	64
225	0	0	0	0	0	23
226	0	0	0	0	0	3
227	0	0	0	0	0	20
228	0	0	0	0	0	20
229	0	0	0	0	0	3
230	0	0	0	0	0	22
231	0	0	0	0	0	7
232	2	7	1	11	2	12
233	2	7	1	10	2	5
234	2	8	1	4	2	6
235	0	0	0	0	0	5
236	0	0	0	0	0	8
237	0	0	0	0	0	8
238	0	0	0	0	0	6
239	0	0	0	0	0	9
240	0	0	0	0	0	8
241	0	0	0	0	0	7

242	0	0	0	0	0	25
243	0	0	0	0	0	5
244	0	0	0	0	0	13
245	0	0	0	0	0	22
246	7	6	1	5	3	15
247	2	7	1	4	2	12
248	0	0	0	0	0	7
249	2	7	1	3	2	6
250	0	0	0	0	0	3
251	1	7	1	2	2	16
252	0	0	0	0	0	2
253	2	8	1	5	2	50
254	1	7	1	2	2	25
255	1	7	1	6	2	11
256	1	7	1	11	2	21
257	0	0	0	0	0	8
258	2	5	1	1	2	7
259	4	6	1	3	3	10
260	1	7	1	1	2	24
261	0	0	0	0	0	25
262	5	6	1	6	2	22
263	1	7	1	5	2	11
264	0	0	0	0	0	7
265	0	0	0	0	0	8
266	3	6	1	1	2	3

267	3	6	1	5	2	17
268	0	0	0	0	0	18
269	5	5	1	1	2	11
270	0	0	0	0	0	5
271	3	6	1	2	2	3
272	1	7	1	3	2	7
273	4	6	1	1	2	6
274	0	0	0	0	0	4
275	2	8	1	4	3	5
276	1	7	1	8	2	11
277	0	0	0	0	0	19
278	5	6	1	5	2	22
279	0	0	0	0	0	7
280	3	6	1	7	2	11
281	0	0	0	0	0	2
282	3	6	1	9	2	19
283	0	0	0	0	0	12
284	3	6	1	5	2	14
285	7	6	1	17	3	17
286	2	10	1	9	3	17
287	3	6	1	3	2	3
288	0	0	0	0	0	8
289	3	6	1	2	2	13
290	0	0	0	0	0	8
291	6	5	1	1	2	7

292	3	6	1	3	2	13
293	0	0	0	0	0	20
294	7	6	1	2	2	14
295	6	6	1	2	2	11
296	3	5	1	1	2	6
297	2	7	1	11	2	15
298	4	6	1	1	2	9
299	2	7	1	11	2	30
300	5	6	1	1	2	6
301	3	6	1	7	2	6
302	3	6	1	14	2	19
303	0	0	0	0	0	9
304	0	0	0	0	0	13
305	5	6	1	4	3	3
306	2	7	1	30	3	19
307	3	6	1	1	2	20
308	0	0	0	0	0	15
309	4	6	1	1	3	13
310	2	7	1	2	2	5
311	0	0	0	0	0	13
312	0	0	0	0	0	16
313	2	5	1	1	3	17
314	0	0	0	0	0	4
315	0	0	0	0	0	18
316	1	7	1	7	2	20

317	3	6	1	1	3	16
318	0	0	0	0	0	8
319	1	7	1	8	2	33
320	0	0	0	0	0	8
321	1	7	1	23	2	13
322	1	7	1	9	2	10
323	0	0	0	0	0	8
324	1	7	1	13	2	15
325	0	0	0	0	0	14
326	6	6	1	4	2	8
327	0	0	0	0	0	5
328	0	0	0	0	0	6
329	1	7	1	9	2	22
330	1	7	1	3	2	10
331	3	6	1	3	2	8
332	3	6	1	2	2	10
333	0	0	0	0	0	15
334	0	0	0	0	0	6
335	1	7	1	13	2	20
336	6	6	1	4	2	11
337	0	0	0	0	0	9
338	3	6	1	1	2	16
339	6	5	1	1	2	3
340	1	7	1	3	2	11
341	1	7	1	21	2	17

342	0	0	0	0	0	4
343	0	0	0	0	0	9
344	3	6	1	3	2	4
345	0	0	0	0	0	6
346	1	7	1	2	2	4
347	4	6	1	9	2	20
348	0	0	0	0	0	24
349	3	6	1	1	2	3
350	0	0	0	0	0	3
351	3	6	1	1	2	11
352	0	0	0	0	0	9
353	4	6	1	2	2	14
354	0	0	0	0	0	4
355	3	6	1	3	2	4
356	3	6	1	12	2	17
357	0	0	0	0	0	1
358	0	0	0	0	0	6
359	3	6	1	3	2	7
360	4	5	1	1	2	6
361	4	5	1	1	2	9
362	0	0	0	0	0	20
363	1	7	1	11	2	32
364	1	7	1	27	2	15
365	1	7	1	5	2	15
366	0	0	0	0	0	3

367	0	0	0	0	0	12
368	0	0	0	0	0	10
369	3	6	1	2	2	5
370	0	0	0	0	0	13
371	0	0	0	0	0	5
372	0	0	0	0	0	17
373	0	0	0	0	0	5
374	0	0	0	0	0	5
375	0	0	0	0	0	7
376	0	0	0	0	0	7
377	4	6	1	7	2	19
378	3	6	1	5	2	4
379	1	7	1	3	2	8
380	1	7	1	5	2	36
381	3	5	1	1	2	10
382	1	7	1	30	2	31
383	2	7	1	6	2	16
384	0	0	0	0	0	8
385	2	7	1	1	2	14
386	2	7	1	4	2	4
387	0	0	0	0	0	8
388	6	6	1	2	2	7
389	2	7	1	2	2	7
390	2	7	1	2	2	10
391	4	6	1	2	2	10

392	0	0	0	0	0	2
393	0	0	0	0	0	1
394	0	0	0	0	0	3
395	3	6	1	4	2	7
396	2	5	1	2	2	8
397	0	0	0	0	0	5
398	0	0	0	0	0	9
399	3	6	1	1	2	9
400	2	5	1	2	2	10
401	0	0	0	0	0	7
402	2	7	1	7	3	20
403	1	7	1	2	2	12
404	3	6	1	3	1	7
405	0	0	0	0	0	7
406	0	0	0	0	0	16
407	3	6	1	1	3	9
408	4	6	1	2	2	8
409	0	0	0	0	0	8
410	3	6	1	5	2	10
411	0	0	0	0	0	6
412	0	0	0	0	0	11
413	0	0	0	0	0	5
414	0	0	0	0	0	7
415	4	6	1	2	2	7
416	1	7	1	3	2	8

417	0	0	0	0	0	10
418	0	0	0	0	0	10
419	0	0	0	0	0	22
420	1	7	1	2	2	15
421	1	7	1	4	3	6
422	4	6	1	5	1	21
423	3	6	1	4	2	11
424	0	0	0	0	0	8
425	1	7	1	21	2	8
426	0	0	0	0	0	1
427	0	0	0	0	0	9
428	1	7	1	35	2	22
429	1	7	1	2	2	4
430	0	0	0	0	0	9
431	3	6	1	2	2	24
432	0	0	0	0	0	27
433	2	5	1	1	2	9
434	4	5	1	1	2	15
435	1	7	1	3	2	3
436	4	6	1	4	2	9
437	0	0	0	0	0	3
438	1	7	1	5	2	7
439	0	0	0	0	0	24
440	1	7	1	1	2	13
441	3	6	1	3	2	13

442	0	0	0	0	0	16
443	0	0	0	0	0	8
444	7	6	1	8	2	14
445	0	0	0	0	0	6
446	3	6	1	1	2	7
447	3	6	1	1	2	7
448	5	5	1	1	2	12
449	3	6	1	2	2	23
450	0	0	0	0	0	10
451	0	0	0	0	0	4
452	7	5	1	3	2	10
453	3	6	1	1	2	4
454	1	7	1	6	2	10
455	0	0	0	0	0	13
456	0	0	0	0	0	32
457	0	0	0	0	0	7
458	0	0	0	0	0	11
459	3	6	1	1	1	7
460	0	0	0	0	0	5
461	1	7	1	4	2	7
462	3	6	1	1	2	12
463	0	0	0	0	0	15
464	0	0	0	0	0	4
465	7	6	1	1	2	8
466	1	7	1	4	2	9

467	0	0	0	0	0	3
468	0	0	0	0	0	7
469	1	7	1	3	2	21
470	3	6	1	2	2	12
471	0	0	0	0	0	19
472	2	8	1	3	2	14
473	3	6	1	4	2	8
474	0	0	0	0	0	2
475	7	6	1	1	2	3
476	0	0	0	0	0	2
477	0	0	0	0	0	3
478	0	0	0	0	0	11
479	0	0	0	0	0	10
480	1	7	1	2	1	6
481	1	7	1	10	3	9
482	3	6	1	4	3	9
483	0	0	0	0	0	9
484	1	7	1	8	2	9
485	0	0	0	0	0	5
486	2	8	1	23	2	4
487	1	7	1	2	2	2
488	0	0	0	0	0	1
489	1	7	1	1	3	8
490	0	0	0	0	0	9
491	2	8	1	3	2	28

492	1	7	1	1	2	6
493	5	6	1	2	2	12
494	1	7	1	2	2	23
495	0	0	0	0	0	14
496	1	7	1	1	2	14
497	2	8	1	12	2	24
498	1	7	1	10	2	14
499	5	6	1	3	2	18
500	0	0	0	0	0	10
501	2	8	1	1	2	11
502	7	6	1	6	2	12
503	1	7	1	5	2	11
504	2	8	1	13	1	33
505	2	8	1	4	2	9
506	0	0	0	0	0	6
507	7	6	1	1	2	7
508	0	0	0	0	0	15
509	3	6	1	2	2	8
510	3	6	1	1	2	8
511	0	0	0	0	0	9
512	0	0	0	0	0	2
513	4	5	1	1	2	19
514	0	0	0	0	0	10
515	0	0	0	0	0	2
516	0	0	0	0	0	3

517	0	0	0	0	0	6
518	3	6	1	2	2	3
519	0	0	0	0	0	5
520	0	0	0	0	0	8
521	0	0	0	0	0	9
522	0	0	0	0	0	15
523	4	6	1	6	2	24
524	0	0	0	0	0	8
525	3	6	1	5	2	7
526	4	6	1	4	2	19
527	0	0	0	0	0	27
528	0	0	0	0	0	2
529	0	0	0	0	0	3
530	0	0	0	0	0	4
531	0	0	0	0	0	12
532	1	7	1	10	2	21
533	0	0	0	0	0	6
534	0	0	0	0	0	10
535	1	7	1	1	1	11
536	1	7	1	5	2	48
537	3	6	1	4	2	12
538	0	0	0	0	0	14
539	1	7	1	4	3	51
540	0	0	0	0	0	10
541	0	0	0	0	0	11

542	0	0	0	0	0	8
543	0	0	0	0	0	9
544	0	0	0	0	0	16
545	1	7	1	10	3	14
546	3	6	1	2	2	8
547	3	6	1	9	2	9
548	0	0	0	0	0	15
549	7	5	1	1	2	10
550	1	7	1	5	2	13
551	0	0	0	0	0	5
552	1	7	1	24	2	34
553	3	6	1	2	2	19
554	1	7	1	5	2	7
555	2	8	1	1	3	8
556	1	7	1	2	2	7
557	1	7	1	6	2	15
558	7	6	1	3	2	7
559	1	7	1	8	2	11
560	2	8	1	3	2	18
561	0	0	0	0	0	3
562	0	0	0	0	0	49
563	0	0	0	0	0	19
564	0	0	0	0	0	10
565	1	7	1	3	2	5
566	0	0	0	0	0	14

567	0	0	0	0	0	9
568	1	7	1	9	2	14
569	0	0	0	0	0	9
570	1	7	1	3	2	16
571	1	7	1	9	2	15
572	5	5	1	1	2	7
573	0	0	0	0	0	6
574	0	0	0	0	0	3
575	0	0	0	0	0	3
576	2	8	1	1	3	8
577	0	0	0	0	0	12
578	1	7	1	3	2	1
579	5	6	1	1	2	11
580	3	6	1	1	2	3
581	0	0	0	0	0	16
582	0	0	0	0	0	4
583	0	0	0	0	0	5
584	0	0	0	0	0	7
585	0	0	0	0	0	7
586	4	6	1	1	2	4
587	4	5	1	2	2	20
588	0	0	0	0	0	5
589	4	5	1	1	1	22
590	4	6	1	1	2	2
591	1	7	1	4	2	23

592	0	0	0	0	0	3
593	1	7	1	4	2	15
594	1	7	1	8	2	20
595	7	6	1	2	2	18
596	0	0	0	0	0	7
597	0	0	0	0	0	17
598	0	0	0	0	0	9
599	0	0	0	0	0	10
600	0	0	0	0	0	8
601	0	0	0	0	0	8
602	0	0	0	0	0	8
603	1	7	1	2	2	31
604	0	0	0	0	0	3
605	0	0	0	0	0	14
606	0	0	0	0	0	7
607	0	0	0	0	0	11
608	0	0	0	0	0	51
609	0	0	0	0	0	15
610	3	6	1	2	2	6
611	1	7	1	7	2	20
612	0	0	0	0	0	23
613	5	6	1	5	2	25
614	0	0	0	0	0	10
615	0	0	0	0	0	3
616	0	0	0	0	0	3

617	0	0	0	0	0	8
618	4	6	1	1	2	31
619	3	6	1	6	2	18
620	4	5	1	2	2	28
621	0	0	0	0	0	4
622	1	7	1	1	3	9
623	0	0	0	0	0	25
624	1	7	1	4	2	13
625	1	7	1	11	2	17
626	1	7	1	7	3	6
627	1	7	1	4	2	11
628	0	0	0	0	0	2
629	0	0	0	0	0	7
630	0	0	0	0	0	6
631	1	7	1	6	2	11
632	0	0	0	0	0	9
633	0	0	0	0	0	6
634	0	0	0	0	0	9
635	0	0	0	0	0	8
636	1	7	1	3	2	14
637	0	0	0	0	0	1
638	1	7	1	4	2	12
639	0	0	0	0	0	17
640	0	0	0	0	0	23
641	0	0	0	0	0	7

642	3	6	1	2	2	7
643	0	0	0	0	0	7
644	1	7	1	13	2	30
645	1	7	1	11	2	21
646	1	7	1	14	3	23
647	1	7	1	9	2	6
648	0	0	0	0	0	11
649	0	0	0	0	0	3
650	0	0	0	0	0	8
651	4	6	1	2	3	8
652	0	0	0	0	0	4
653	1	7	1	3	3	5
654	4	6	1	6	2	13
655	3	6	1	1	2	22
656	0	0	0	0	0	6
657	7	5	1	1	3	1
658	0	0	0	0	0	5
659	6	5	1	1	2	7
660	6	5	1	1	2	23
661	1	7	1	10	2	19
662	6	6	1	1	2	10
663	1	7	1	3	3	5
664	4	6	1	2	2	11
665	0	0	0	0	0	5
666	1	7	1	5	2	26

667	0	0	0	0	0	11
668	4	5	1	1	2	10
669	4	6	1	6	3	9
670	6	6	1	2	2	8
671	0	0	0	0	0	12
672	0	0	0	0	0	8
673	0	0	0	0	0	13
674	0	0	0	0	0	4
675	1	7	1	4	5	7
676	0	0	0	0	0	7
677	0	0	0	0	0	7
678	0	0	0	0	0	5
679	0	0	0	0	0	21
680	0	0	0	0	0	10
681	5	6	1	2	2	9
682	0	0	0	0	0	2
683	0	0	0	0	0	7
684	0	0	0	0	0	11
685	1	7	1	7	3	22
686	1	7	1	2	3	9
687	1	7	1	4	3	20
688	5	5	1	2	2	9
689	0	0	0	0	0	24
690	0	0	0	0	0	1
691	1	7	1	8	3	11

692	1	7	1	12	3	40
693	0	0	0	0	0	5
694	2	8	1	8	4	10
695	3	6	1	4	2	29
696	2	9	1	7	3	7
697	0	0	0	0	0	7
698	5	5	1	2	2	11
699	0	0	0	0	0	12
700	5	6	1	1	2	18
701	6	5	1	1	2	17
702	0	0	0	0	0	14
703	3	6	1	3	2	14
704	0	0	0	0	0	7
705	0	0	0	0	0	6
706	6	6	1	4	2	17
707	0	0	0	0	0	7
708	1	7	1	15	3	8
709	6	6	1	3	2	10
710	0	0	0	0	0	8
711	0	0	0	0	0	1
712	1	7	1	20	2	30
713	7	6	1	2	3	4
714	7	5	1	1	2	8
715	7	5	1	1	3	2
716	0	0	0	0	0	1

717	1	7	1	1	2	8
718	0	0	0	0	0	8
719	1	7	1	1	2	8
720	1	7	1	6	3	10
721	7	5	1	1	3	7
722	0	0	0	0	0	24
723	7	5	1	2	3	13
724	0	0	0	0	0	13
725	0	0	0	0	0	7
726	1	7	1	10	2	16
727	6	6	1	4	2	8
728	0	0	0	0	0	5
729	1	7	1	9	2	22
730	6	5	1	1	2	10
731	0	0	0	0	0	12
732	1	7	1	8	3	12
733	0	0	0	0	0	5
734	0	0	0	0	0	6
735	6	6	1	4	2	7
736	0	0	0	0	0	4
737	0	0	0	0	0	13
738	0	0	0	0	0	4
739	0	0	0	0	0	14
740	1	7	1	3	2	17
741	0	0	0	0	0	7

742	3	5	1	1	3	6
743	5	6	1	16	2	12
744	0	0	0	0	0	31
745	0	0	0	0	0	3
746	0	0	0	0	0	12
747	3	7	1	4	3	48
748	2	7	1	3	3	8
749	0	0	0	0	0	8
750	2	7	1	3	2	6
751	0	0	0	0	0	4
752	0	0	0	0	0	5
753	2	5	1	1	2	8
754	0	0	0	0	0	9
755	0	0	0	0	0	2
756	0	0	0	0	0	5
757	5	5	1	2	2	7
758	0	0	0	0	0	1
759	3	6	1	5	2	7
760	0	0	0	0	0	1
761	0	0	0	0	0	6
762	0	0	0	0	0	2
763	0	0	0	0	0	19
764	0	0	0	0	0	8
765	5	5	1	1	2	14
766	0	0	0	0	0	7

767	0	0	0	0	0	18
768	0	0	0	0	0	15
769	0	0	0	0	0	14
770	2	7	1	5	2	11
771	0	0	0	0	0	13
772	0	0	0	0	0	20
773	0	0	0	0	0	6
774	0	0	0	0	0	13
775	3	7	1	3	2	15
776	0	0	0	0	0	4
777	5	7	1	16	2	38
778	0	0	0	0	0	8
779	2	7	1	12	2	22
780	3	6	1	4	2	13
781	6	6	1	3	2	9
782	2	7	1	5	2	8
783	2	7	1	2	3	2
784	0	0	0	0	0	11
785	3	6	1	2	3	1
786	2	7	1	2	2	9
787	0	0	0	0	0	7
788	0	0	0	0	0	6
789	2	7	1	1	2	7
790	2	7	1	2	2	6
791	0	0	0	0	0	24

792	3	7	1	6	2	18
793	0	0	0	0	0	6
794	6	7	1	3	2	14
795	2	6	1	1	2	10
796	2	5	1	2	2	11
797	2	7	1	5	2	19
798	3	7	1	6	2	8
